# A Targeted Multi-omic Analysis Approach Measures Protein Expression and Low-Abundance Transcripts on the Single-Cell Level

**DOI:** 10.1016/j.celrep.2020.03.063

**Published:** 2020-04-07

**Authors:** Florian Mair, Jami R. Erickson, Valentin Voillet, Yannick Simoni, Timothy Bi, Aaron J. Tyznik, Jody Martin, Raphael Gottardo, Evan W. Newell, Martin Prlic

**Affiliations:** 1 Fred Hutchinson Cancer Research Center, Vaccine and Infectious Disease Division, Seattle, WA 98109, USA; 2 BD Biosciences, La Jolla, CA 92037, USA; 3 Fred Hutchinson Cancer Research Center, Public Health Sciences Division, Seattle, WA 98109, USA; 4 Department of Statistics, University of Washington, Seattle, WA 98195, USA; 5 Department of Global Health and Department of Immunology, University of Washington, Seattle, WA 98195, USA; 6 These authors contributed equally; 7 Lead Contact

## Abstract

High-throughput single-cell RNA sequencing (scRNA-seq) has become a frequently used tool to assess immune cell heterogeneity. Recently, the combined measurement of RNA and protein expression was developed, commonly known as cellular indexing of transcriptomes and epitopes by sequencing (CITE-seq). Acquisition of protein expression data along with transcriptome data resolves some of the limitations inherent to only assessing transcripts but also nearly doubles the sequencing read depth required per single cell. Furthermore, there is still a paucity of analysis tools tovisualize combined transcript-protein datasets. Here, we describe a targeted transcriptomics approach that combines an analysis of over 400 genes with simultaneous measurement of over 40 proteins on 2 × 10^4^ cells in a single experiment. This targeted approach requires only about one-tenth of the read depth compared to a whole-transcriptome approach while retaining high sensitivity for low abundance transcripts. To analyze these multi-omic datasets, we adapted one-dimensional soli expression by nonlinear stochastic embedding (One-SENSE) for intuitive visualization of protein-transcript relationships on a single-cell level.

## In Brief

Mair et al. describe a targeted transcriptomics approach combined with surface protein measurement to capture immune cell heterogeneity at a low sequencing depth. One-SENSE is used as a visualization tool to intuitively explore the relationship of protein and transcript expression on the single-cell level.

## Graphical Abstract

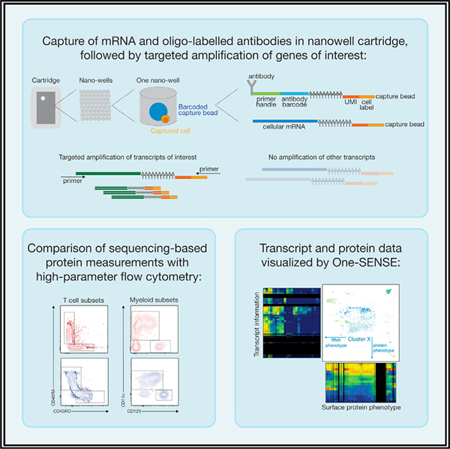

## INTRODUCTION

Pioneering work almost 20 years ago illustrated the ability to study transcript expression at the single-cell level (Chiang and Melton, 2003; Phillips and Eberwine, 1996), but recent advances in microfluidics and reagents allow the high-throughput analysis of transcripts of 10^4^ single cells in one experiment (Jaitin et al., 2014; Klein et al., 2015; Macosko et al., 2015). Several methods have been developed for this purpose, and currently the most widely adopted platform is a droplet-based microfluidics system commercialized by 10x Genomics (Zheng et al., 2017).

Although analysis of transcript expression on the single-cell level is a powerful tool to characterize the phenotypic and functional properties of cells, it is imperative to consider the relationship between transcripts and proteins when trying to extrapolate biology. Typically, transcripts are expressed at a much lower level than proteins—for example, murine liver cells have a median copy number of 43,100 proteins but only 3.7 mRNA molecules per gene (Azimifar et al., 2014). Similarly, the dynamic range of expression is much greater for proteins, with copy numbers spanning about 6–7 orders of magnitude, whereas transcript copy numbers span about 2 orders of magnitude (Schwanhäusser et al., 2011). Finally, the correlation of gene expression and protein expression has been estimated to have a Pearson correlation coefficient between 0.4 (Schwanhäusser et al., 2011) and 0.6 (Azimifar et al., 2014). These discrepancies in transcript and protein expression patterns are relevant for the biological interpretation of single-cell transcriptome data but also pose analytical challenges. Suitable approaches are required to visualize the data despite the pronounced differences in abundance and dynamic range of expression.

The parallel measurement of transcript and protein phenotype has been recently reported as cellular indexing of transcriptomes and epitopes by sequencing (CITE-seq) (Stoeckius et al., 2017) or RNA expression and protein sequencing (REAP-seq) (Peterson et al., 2017). These technologies leverage existing single-cell RNA sequencing (scRNA-seq) platforms that use an unbiased whole-transcriptome analysis (WTA) approach that captures cellular mRNA by its poly-A tail and use oligonucleotide-labeled antibodies (carrying unique barcodes) to interrogate surface protein abundance. Typically, current droplet-based WTA approaches result in the detection of ~1,000 unique transcripts per single cell for the transcriptome (with a substantial fraction of these transcripts encoding ribosomal proteins), and antibody panels of up to 80 targets have been reported (Peterson et al., 2017).

Although proof-of-principle experiments for this sequencing-based technology have been established, it remains unclear how the antibody detection compares to established flow-cytometry-based assays in different experimental settings with regard to capturing the dynamic range of protein expression and identifying low abundance protein expression. In addition, the combined WTA plus protein approach can quickly become resource intensive given the high number of reads per cell required to achieve library saturation. Finally, droplet-based WTA pipelines may still miss specific transcripts of interest if they are below the limit of detection, with current high-throughput chemistries capturing an estimated 10% of the total cellular mRNA (Zheng et al., 2017).

Here, we report using a high-throughput (>10^4^ single cells) targeted transcriptomics approach using nanowells to capture single cells (Rhapsody platform, commercialized by BD Biosciences) (Fan et al., 2015) in combination with oligonucleotide-barcoded antibodies (termed AbSeq). Specifically, we simultaneously interrogated 492 immune-related genes and 41 surface proteins that are commonly used for immunophenotyping. We found that this targeted approach was efficient at detecting low-abundance transcripts while only requiring about one-tenth of the sequencing read depth needed for WTA, indicating that targeted transcriptomics is a sensitive and cost-efficient alternative when the focus is on interrogating defined transcripts. Notably, this approach clearly separated different memory T cell subsets as well as regulatory T cells (Tregs) solely based on transcript information, which is often difficult due to the low amount of mRNA recovered from T lymphocytes (Zheng et al., 2017). Furthermore, we used 30-parameter fluorescent-based flow cytometry to simultaneously measure the same protein targets as in the multi-omic assay. Our data indicate that the validation of oligonucleotide-barcoded antibody panels is necessary for meaningful interpretation of the multi-omic data.

To demonstrate the sensitivity and robustness of the system, we analyzed T cells before and after 1 h of stimulation. We found distinct chemokine expression patterns within the CD8^+^ T cell population 1 h after stimulation, indicating significant heterogeneity in the response of this compartment.

Finally, to intuitively visualize protein and transcriptome data in a single plot, we adapted one-dimensional soli expression by nonlinear stochastic embedding (One-SENSE), which was originally developed for visualization of mass cytometry data (Cheng et al., 2016). This adaptation allows effective visualization and identification of cellular phenotypes that differ either by transcripts or by proteins. Overall, we provide a methodological toolset for generating high-throughput multi-omic single-cell data with a focus on selected targets at minimal read depth and an analytical tool to visualize these protein and transcript datasets.

## RESULTS

### Comparison of Oligonucleotide-Labeled Antibody Probes to High-Dimensional Flow Cytometry

**Figure 1.**
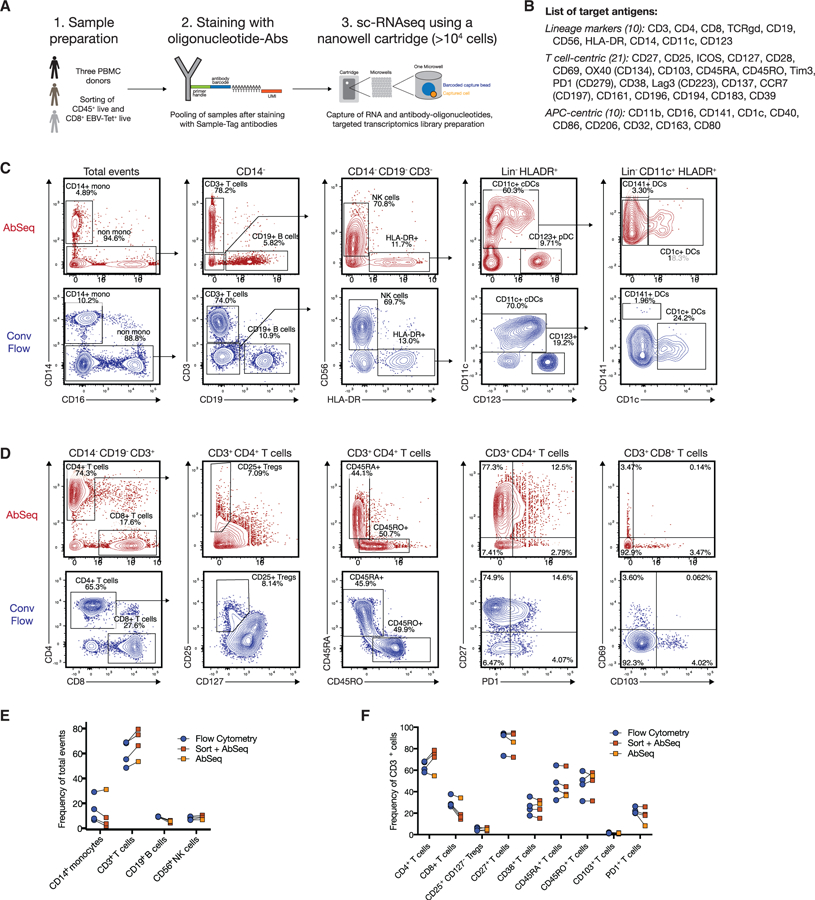
Comparison of Oligonucleotide-Labeled Antibody Probes to High-Dimensional Flow Cytometry (A) Schematic graph describing the workflow of the experiment. PBMC samples from three donors were split in half, with one aliquot used for the multi-omic workflow and one aliquot used for flow cytometry phenotyping using two 30-parameter panels. (B) Overview of antibody targets used in both the multi-omic and conventional flow cytometry experiment. (C) Manual gating of main immune subsets by using the combined AbSeq dataset (top panel, red) and concatenated and down-sampled events (27,000 cells, from three donors) from the conventional (conv) flow cytometry dataset (bottom panel, blue). (D) Manual gating of various T cell markers by using the combined AbSeq dataset (top panel, red) and concatenated, down-sampled events from the cytometry dataset (bottom panel, blue). (E) Quantification of main immune subsets by using AbSeq and flow cytometry either with prior cell sorting (red squares) or using AbSeq without prior cell sorting (orange squares). (F) Quantification of main T cell populations and selected phenotyping markers from two independent experiments using AbSeq and flow cytometry either with prior cell sorting (red squares) or using AbSeq without prior cell sorting (orange squares). See also Figure S1 and Table S1 for full list of genes.

For our reference dataset, we used peripheral blood mononuclear cells (PBMCs) from three healthy control subjects carrying the HLA-A*02:01 allele, which allowed isolation of Epstein-Barr Virus (EBV)-specific CD8^+^ T cells by using an EBV-tetramer reagent (EBV epitope YVLDHLIVV) (Dunne et al., 2002). To ensure sufficient cell numbers of these rare, antigen-specific T cells, we enriched tetramer-positive T cells by fluorescence-activated cell sorting (FACS). In parallel, we sorted CD45^+^ live leukocytes from PBMCs (Figure 1A). Moreover, to minimize batch effects during subsequent staining with 41 oligo-nucleotide-labeled antibodies (Figure 1B), we used a multiplexing protocol with barcoded cell-hashing antibodies (Stoeckius et al., 2018). All samples were processed simultaneously using the Rhapsody platform, a nanowell-based cartridge system (Fan et al., 2015) for scRNA-seq with a targeted approach focusing on 492 immune-relevant transcripts (397 pre-defined targets complemented by 95 experiment-specific targets, full list provided in Table S1). Following quality control and removal of multiplets, we recovered 27,258 cells from the sequencing data, which were evenly distributed across the three different donors (donor 1: 8,984 cells, donor 2: 8,956 cells, donor 3: 9,318 cells).

First, we wanted to assess whether the surface protein phenotypes as defined by sequencing match known biology. For this, we designed two optimized 30-parameter immunophenotyping panels (adapted from Mair and Prlic, 2018) covering the same 41 protein targets in an overlapping fashion. We used these panels to stain whole unsorted PBMC samples from the same 3 donors, down-sampled the cytometry data to 27,000 cells, and used biaxial gating to identify the main immune lineages of the myeloid compartment (Figure 1C) as well as the lymphoid compartment (Figure 1D). All populations were present at comparable frequencies in the two different datasets, with myeloid cells showing slightly lower abundance in the experiment that included cell sorting (Figures 1E and 1F). Notably, even low-abundance cell populations, such as CD1c^+^ conventional dendritic cells (cDCs) and cross-presenting CD141^+^ cDCs, were clearly identified by their surface protein phenotype (Figure 1C). Furthermore, the oligonucleotide-labeled antibodies allow discrimination of the CD45 splice variants CD45RO and CD45RA, which are used to subset T cells and cannot be distinguished by 3ʹtranscriptomic analysis alone.

However, for the anti-T cell receptor ɣδ (TCR ɣδ) reagent we used, discordant patterns were observed when comparing the expression to conventional flow cytometry, despite using the same antibody clone (Figure S1A). This was not immediately evident when visualizing the data on a heatmap (Figure S1B), emphasizing the need for careful reagent validation for sequencing-based protein measurements. Thus, we did not analyze ɣδ T cells separately for the rest of our study. Furthermore, the anti-CCR7 antibody clone used in the AbSeq experiment delivered sub-optimal resolution (data not shown). A detailed list of antibody clones and used concentrations can be found in the STAR Methods (Table S4).

### Targeted Transcriptomics Captures the Major PBMC Lineages Similar to Whole-Transcriptome Approaches

**Figure 2.**
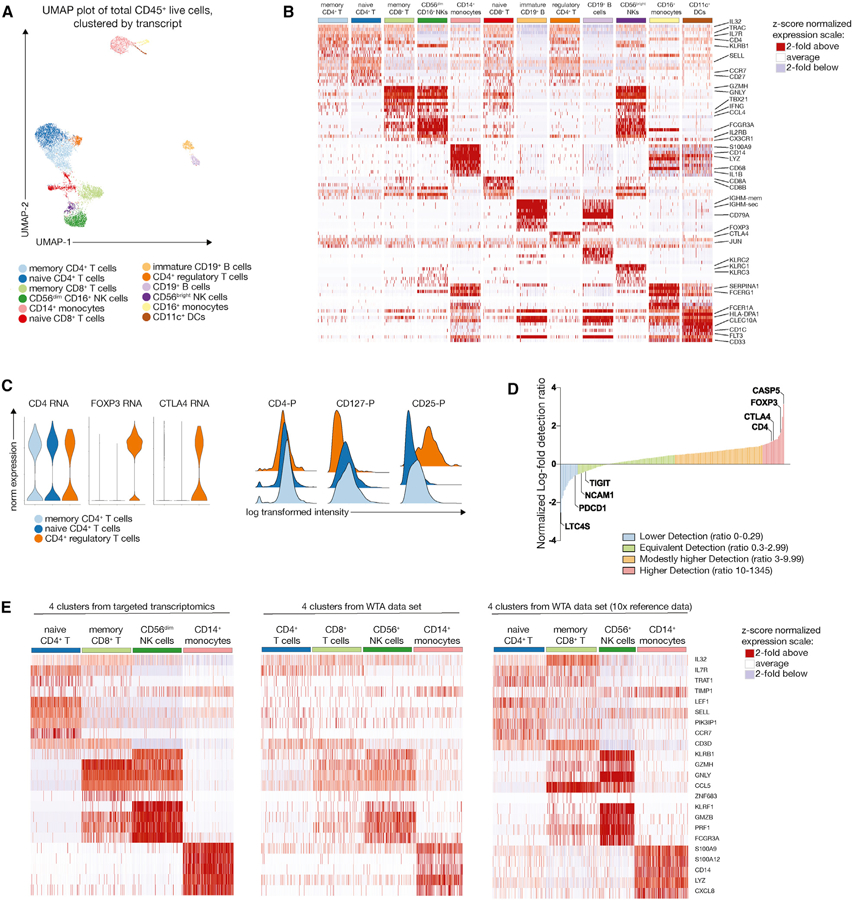
Targeted Transcriptomics Captures the Major PBMC Lineages Similar to Whole-Transcriptome Approaches (A) Graph-based clustering of the transcript data from one representative donor (8,843 cells) is shown on a UMAP (uniform manifold approximation projection) plot. Clusters have been annotated by expression of key lineage genes. (B) The top 10 differentially expressed genes for each cluster were identified using the Seurat implementation of MAST (model-based analysis of single-cell transcriptomes) and visualized on a heatmap after *Z* score normalization. Cluster names are shown in the same color scheme as in (A). (C) Expression of the indicated transcripts and proteins on the three different CD4+ T cell clusters, highlighting the CD25^+^ CD127^low^ Treg cluster (orange). (D) Relative detection ratio of all detected transcripts relative to a whole-transcriptome dataset from the same donor. Genes are manually assigned into four different groups according to their relative detection ratio. (E) Expression pattern of the top 5 differentially expressed genes for each cluster (as identified by MAST on the targeted transcriptomics dataset) for 4 representative main immune populations on the targeted data (left), whole-transcriptome data from the same donor (middle), and a publicly available whole-transcriptome reference dataset (right). See also Figures S2 and S3.

Next, we wanted to assess how well a targeted transcriptomics approach can identify immune cell heterogeneity compared to a commonly used WTA pipeline (Zheng et al., 2017). For this, we used a single donor and compared the resulting populations after graph-based clustering of the transcript data by using the R package Seurat implementation of PhenoGraph at standard resolution settings (Butler et al., 2018; Levine et al., 2015; Figures 2A and S2A). For visualization, we used uniform manifold approximation and projection (UMAP), a dimensionality reduction approach that has recently been adopted for single-cell data (Becht et al., 2019; McInnes et al., 2018). Overall, the targeted transcriptomic approach using 492 genes revealed a similar resolution of known immune subsets in the peripheral blood relative to WTA (Figures 2B and S2B). In particular, CD4^+^ T cells and CD8^+^ T cells separated well, and we observed Tregs expressing FOXP3 and CTLA4 as a separate cluster (Figure 2B). For verification of this Treg cluster, we used the corresponding protein signature, which showed high expression of CD25 and low expression of CD127 (Figure 2C). To determine if we could extract these same clusters from a WTA set, we performed clustering of a publicly available WTA reference dataset at different resolution parameters (Figures S2B and S2C).

Next, to obtain a relative measure of detection efficiency, we calculated the average number of transcripts per cell both for the targeted transcriptomics as well as the WTA dataset from the same donor. Around 75% of the assayed genes showed equal or slightly superior detection efficiencies (Figure 2D). However, although there was a small subset of transcript targets that showed considerably higher detection efficiency (including FOXP3 and CTLA4, identifying the Treg cluster), around 34 targets (including PDCD1) were detected at less than 30% of their level in the WTA dataset (full list and calculation provided in Table S2). These differences are likely due to the different amplification efficiencies using multiplexed targeted primers (Rhapsody) versus primers designed to recognize the template switch-oligo (10x Genomics).

Next, we compared the gene expression pattern for four phenotypically similar clusters (as defined by their transcript profile) in our WTA and the targeted transcriptomics dataset from the same donor, and we included the same clusters from a publicly available WTA reference dataset containing more than 8,000 cells (Figure 2E). Visualizing the top differentially expressed genes (as identified by model-based analysis of single-cell transcriptomes [MAST] in the targeted dataset, see STAR Methods) of these four clusters on a heatmap yielded very similar patterns, suggesting that targeted transcriptomics with an immune-focused primer panel faithfully recapitulates cellular heterogeneity of PBMCs at the single-cell level.

To further compare our donor-matched datasets, we used MAST to identify all differentially expressed genes for each cluster with a log-fold change of >0.25 in both datasets. For three clusters (CD56^+^ natural killer [NK] cells, CD4^+^ T cells, and CD14^+^ monocytes) we compiled a list of genes that were shared between the two datasets or that were only present in the WTA data and only present in the targeted transcriptomics data (full list of genes available in Table S3). Visualizing all overlapping genes for a representative cluster (the NK cell cluster) in both datasets again displayed very similar patterns (Figure S2D). In the case of the NK cell cluster, there were 73 additional genes identified only in the WTA data (heatmap representation of the top 23 of these is shown in Figure S2E). To gain insight into how well biological processes are captured with a targeted set of genes, we performed Gene Ontology (GO) analysis, showing that the majority of GO terms were detected in either dataset, with some GO terms (e.g., GO-0051179, ‘‘localization’’) only being found in the WTA dataset (Figure S2F).

Finally, to directly assess the effect of different read depths on the resolution of protein and transcript signals in the AbSeq dataset, we analyzed PBMCs of a different donor with a total of approximately 27,000 reads/cell (approximately 18,000 reads/cell for the antibody library, 9,000 reads/cell for the transcript library; see STAR Methods for list of all read depths) and subsampled the number of reads during processing of the raw data to 20% (approximately 4,000 reads/cell for the antibody library, 2,000 reads/cell for the transcript library) and 10%. Visualization of the resulting clusters on a UMAP plot as well as the top differentially expressed genes on a heatmap revealed no major differences between using 100% or 20% of the reads (Figure S3A). For the protein signal, differences only became apparent when using 10% of the reads, which resulted in a noticeable loss of signal intensities (Figure S3B). For protein targets that exhibited bimodal distributions, we calculated the absolute number of molecules detected, revealing a noticeable drop in signal intensity when down-sampling to 10% of the reads (Figure S3C).

Overall, we conclude that the panels used in our assay targeting at least 2,000–4,000 reads/cell for the transcript portion of the library (which is approximately one-tenth of the typical read depths used for WTA approaches) (Zheng et al., 2017) and at least 200–400 reads/antibody/cell for the antibody portion of the library deliver sufficient resolution.

### Multi-omic Analysis Identifies Canonical Memory T Cell Populations and Allows the Study of Rare-Antigen-Specific CD8^+^ T Cells

**Figure 3.**
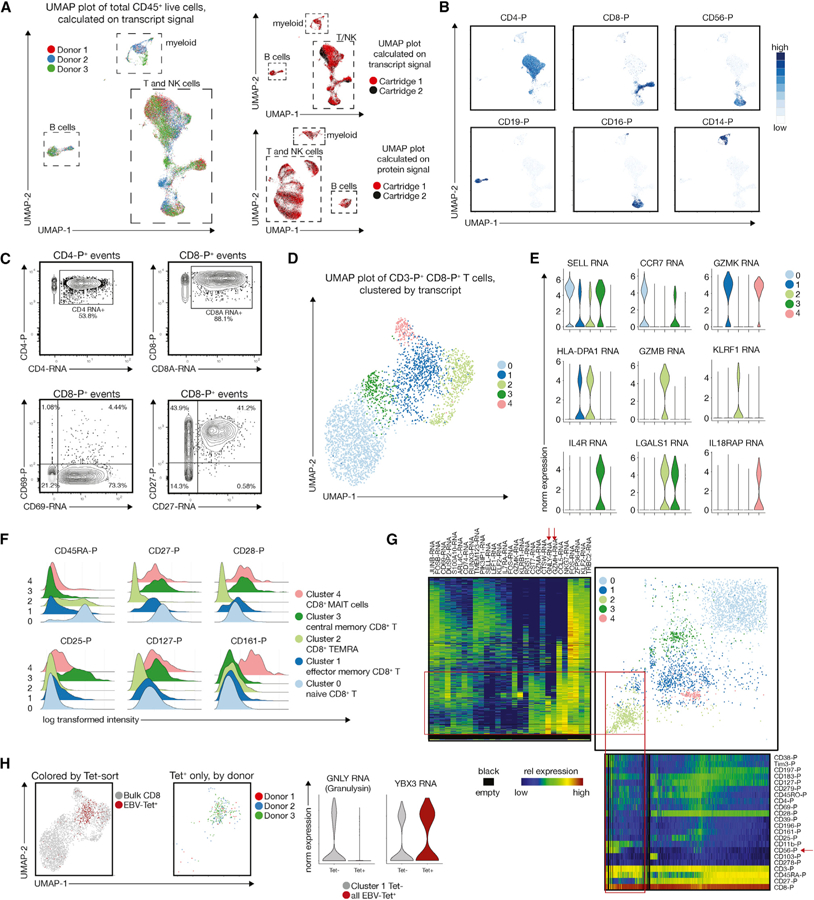
Multi-omic Targeted Transcriptomics Identifies Canonical Memory T Cell Populations and Allows the Study of Rare-Antigen-Specific CD8^+^ T Cells (A) UMAP plots calculated as indicated and colored by donor (left) or by cartridge run (right) show that there are no major clusters driven from the different experimental runs or individual donors. (B) Example UMAP plots (calculated on transcript) representing the expression of the main immune lineage protein markers, which allow the unequivocal identification of CD4^+^ and CD8^+^ T cells, CD19^+^ B cells, and CD14^+^, as well as CD16^+^ myeloid cells. (C) Example bivariate plots showing the poor correlation of transcript and protein levels for CD4 and CD69 and good correlation for CD8 and CD27. Protein signal is plotted on the y-axis, and transcript signal on the x-axis. (D) UMAP plot and graph-based clustering of the CD3^+^ CD8^+^ CD4− T cell compartment, revealing 5 distinct populations. (E) Violin plots showing some of the top differentially expressed genes identified by MAST for each of the 5 clusters in (D). (F) Protein signatures of the 5 clusters identified canonical naive and memory CD8^+^ T cell subsets, including CD8^+^ mucosal-associated invariant T cells (MAIT cells). (G) One-SENSE plot depicting protein expression heatmap along the x-axis, and transcript expression heatmap of the top differentially expressed genes along the y-axis. (H) Identification of EBV-specific CD8^+^ T cells relative to all CD8^+^ T cells, and expression pattern of two differentially expressed genes between tetramer-positive cells and tetramer-negative cells in the effector memory cluster 1.

We next performed an in-depth analysis of the CD8^+^ T cell compartment. First, we visualized protein and RNA data collected from total CD45^+^ live cells from PBMCs from three patents on separate UMAP plots. We found that cells from different donors comingled and separated by cell type rather than by donor, suggesting that there was no cluster driven by a single donor (Figure 3A, left panel). Also, the two experimental batches (cartridges) did not show significant separation on a UMAP plot calculated using transcripts or proteins (Figure 3A, right panel).

Notably, protein information overlaid on the transcript-generated UMAP plot allowed accurate identification of all main immune clusters (Figure 3B), which is not necessarily the case when using transcript information for the corresponding lineage markers. This is exemplified by biaxial plots showing protein signal on the y-axis and transcript signal on the x-axis (Figure 3C). Although CD8A, transcript, and protein are co-expressed in most cells, only half of the CD4-protein^+^ (throughout the manuscript abbreviated as CD4-P) cells contained detectable CD4-transcript. On the contrary, CD69-RNA (plotted on the x-axis) was detected across a large number of CD8^+^ T cells, but as expected only a few CD8^+^ T cells in the peripheral blood express the CD69 protein (CD69-P, plotted on y-axis) on their surface. For CD27, we observed a higher correlation between transcripts and proteins (Figure 3C). Overall, these observations emphasize the importance of the parallel measurement of proteins and transcripts to faithfully study T cell biology.

Next, we continued our analysis of CD8^+^ T cells (defined by surface protein expression) using SCAMP (selected clustering annotated using modes of projections) (Greene et al., 2018). Unbiased graph-based clustering using transcript information suggested the presence of 5 distinct cellular clusters (Figure 3D). Visualization of the top differentially expressed genes, such as SELL (encoding CD62L), CCR7, and GZMB, suggested that these 5 clusters reflect canonical naive and memory T cell populations (Sallusto et al., 1999; Figure 3E). Additionally, our data allowed identification of CD8^+^ mucosal-associated invariant T (MAIT) cells, which express high levels of Interleukin-18 Receptor Accessory Protein (IL18RAP) and Tumor Necrosis Factor (TNF) (Slichter et al., 2016; Mori et al., 2016). We confirmed the resemblance of these populations by surface protein expression (Figure 3F), with central memory CD8^+^ T cells expressing low levels of CD45RA protein and high levels of both CD27 and CD28 protein (Sallusto et al., 2004; Hamann et al., 1997). Importantly, the splice variants CD45RO and CD45RA cannot be distinguished by analyzing transcripts alone, highlighting the added value of combined protein and transcript analysis.

To visualize the correspondence between transcript and protein expression in the multi-omic dataset, we adopted One-SENSE, which has originally been developed for visualizing mass cytometry data (Cheng et al., 2016). We mapped cells by separately plotting proteins and transcripts each on to a single UMAP dimension, similar to a recently published one-dimensional t-stochastic neighbor embedding (t-SNE) representation for scRNA-seq data (Linderman et al., 2019). The combined plot shows the overall distribution of protein expression profiles in the x-axis and the top differentially expressed gene profiles on the y-axis. Aligned heatmaps that represent median expression with bins of cells are provided to annotate the one-dimensional UMAP protein and gene expression profiles (blue: low expression, red: high expression). Black areas in the heatmaps refer to areas of the One-SENSE plot that are devoid of cells. This approach allows easy identification of cellular clusters that are similar by transcripts but separated by proteins and vice versa (Figure 3G). One example for this is highlighted in Figure 3G (red box and arrow), where cluster 2 (light green, TEMRA cells) is relatively homogeneous by transcript but can be separated by CD56 protein expression. A fraction of cells between cluster 1 (dark blue, effector memory CD8^+^ T cells) and 2 (light green, TEMRA) shares the same protein signature but can be distinguished by GNLY and GZMH expression (Figure 3G, red box and arrows). Varying degrees of concordance and ability to discriminate cellular subsets from gene and protein expression profiles can be seen across this plot.

To determine if targeted transcriptomics is amenable for studying rare-antigen-specific T cell populations, we analyzed CD8^+^ T cells recognizing the HLA-A*02-restricted EBV epitope YVLDHLIVV (Dunne et al., 2002). Visualization on the UMAP plot revealed remarkable similarity of EBV-specific T cells across all three donors (Figure 3H), with most of the cells grouped within the effector memory CD8^+^ T cell cluster. However, relative to the EBV-nonspecific memory T cell cluster, the EBV-Tet^+^ T cells showed a significant downregulation of the Granulysin transcripts and an upregulation of YBX3, an RNA binding protein whose function has not been defined in T cells but has recently been shown to be a critical regulator for the stability of specific mRNAs (Cooke et al., 2019).

Overall, these data show that a combined analysis of targeted transcriptomics and protein expression by sequencing is a valuable approach for studying T cell subsets and could be used as a resource-efficient tool for studying T cell responses in human disease.

### Short-Term Stimulation of T and NK Cells Reveals Chemokine Heterogeneity and a Disconnect with the Early Activation Marker CD69

**Figure 4.**
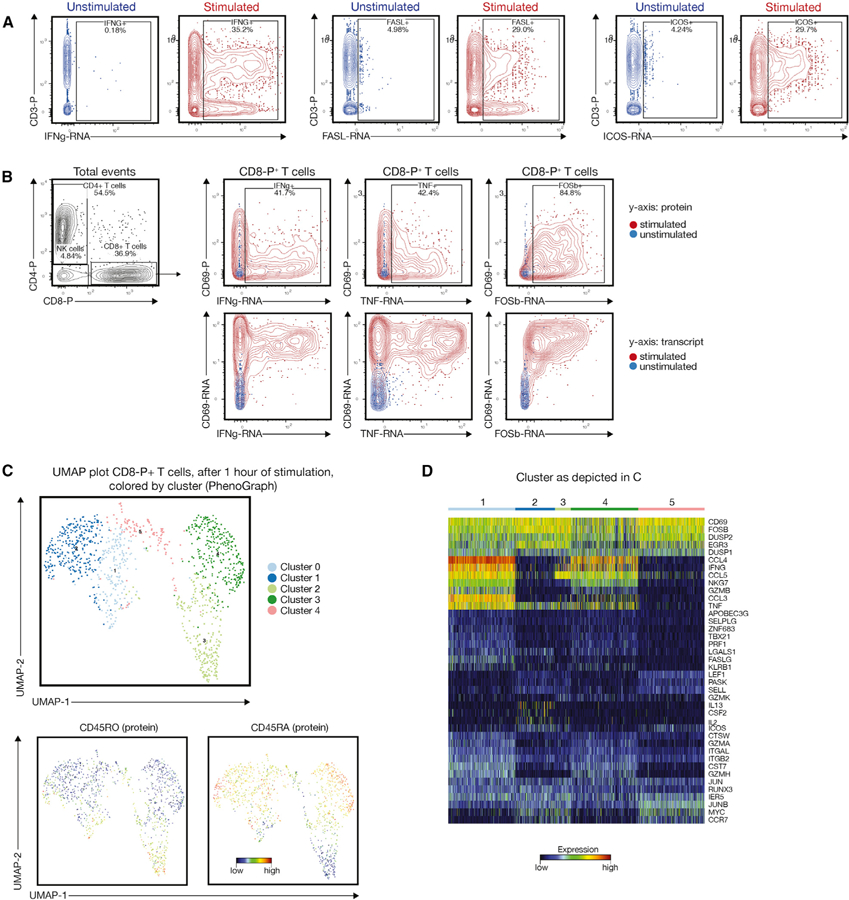
Multi-omic Analysis of the T and NK Cell Compartment 1 h after Stimulation (A) Representative plots showing the upregulation of selected effector transcripts, such as IFNG, FASL, and ICOS, after stimulation (red) relative to unstimulated cells (blue). (B) Disconnect between surface protein expression of the early activation marker CD69 and IFNG and TNF transcript within CD8 protein^+^ T cells. Blue overlay indicates unstimulated cells, and red indicates stimulated cells. (C) UMAP plot of stimulated CD8-protein^+^ T cells showing five phenograph-defined clusters and corresponding CD45RA and CD45RO protein expression. (D) Heatmap showing the expression of key effector transcripts within the clusters identified in (C).

Cytokines and chemokines are the quintessential effector molecules of T cells, and the existence of specific T cell subsets that are poised to produce certain cytokines has been the subject of intense research over the past decade (van den Broek et al., 2018; Zhou et al., 2009). To test whether multi-omic single-cell analysis can provide additional insight, we purified T and NK cells and stimulated them for 1 h with phorbol-myristate-acetate (PMA) and ionomycin. We probed early transcriptional changes with a T-cell-centric-targeted transcriptomic approach covering 259 genes. Transcripts encoding for IFNG, FASL, and ICOS exhibited robust upregulation in the stimulated versus unstimulated sample (Figure 4A), as was the case for CD69, a commonly used protein marker for early T cell activation (Figure 4B). Notably, when we analyzed cytokine expression relative to the surface protein expression of CD69 on CD8^+^ T cells, we observed that both IFNG as well as TNF transcripts were primarily expressed in CD69-transcript-positive, but CD69-protein-negative cells, whereas the FOSB transcript (encoding for a subunit of the transcription factor AP-1) was co-expressed with the CD69 protein (Figure 4B).

Data projection on a UMAP plot after phenograph-based clustering (Levine et al., 2015) suggested the presence of five different clusters (Figure 4C). Protein expression patterns for CD45RA and CD45RO highlight the distribution of naive, effector, and memory T cells within this plot (Figure 4C). A heatmap visualization of the most highly expressed transcripts show that these five clusters are primarily defined by the differential expression of CCL3, CCL4, IFNG, TNF, and various granzymes (Figure 4D). Overall, this analysis reveals considerable functional diversity within the CD8^+^ T cell compartment that is detectable 1 h after stimulation. Given the nature of PMA- and ionomycin-induced activation, this finding likely reflects intrinsic functional heterogeneity.

### Multi-omic Analysis of the Peripheral Myeloid Compartment Reveals Inflammatory Subsets Not Captured by Surface Protein Phenotype

**Figure 5.**
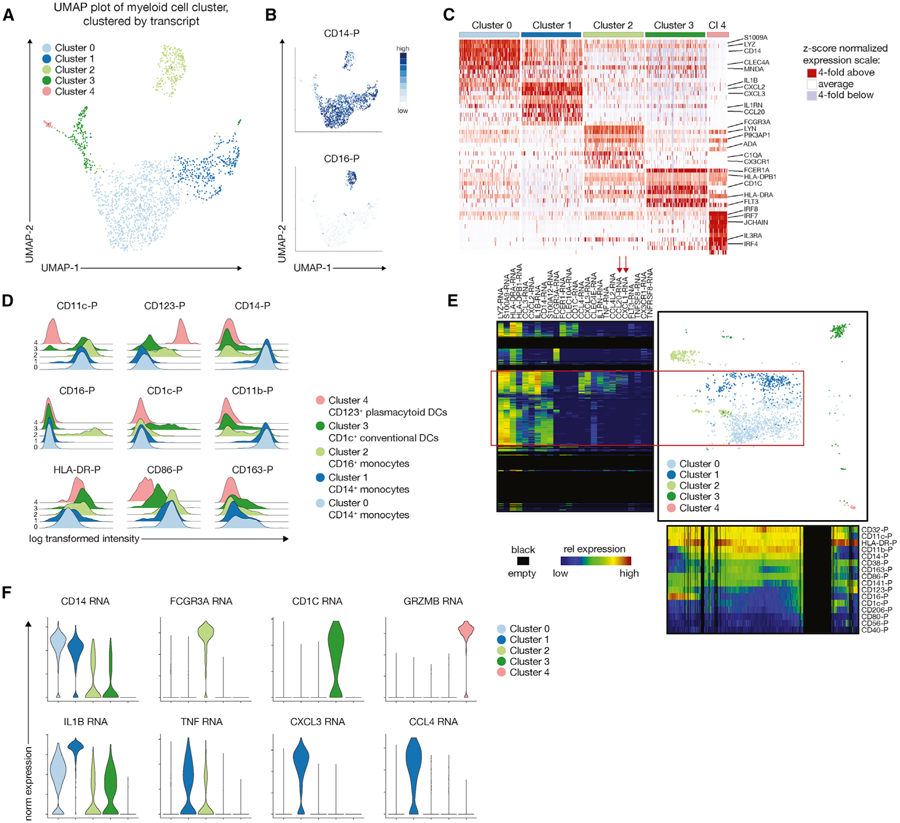
Combined Protein and Transcript Phenotyping of the Peripheral Myeloid Compartment Reveals Inflammatory Subsets Not Captured by Surface Protein Phenotype (A) UMAP plot and graph-based clustering of the peripheral non-T/non-NK/non-B cell compartment, revealing 5 distinct populations. (B) Heatmap overlay of CD14 and CD16 protein expression. (C) Heatmap of the top differentially expressed genes identified by MAST for each of the 5 clusters highlighted in (A). (D) Protein signatures of the 5 clusters identifies canonical CD123^+^ plasmacytoid DCs, CD1c^+^ conventional DCs, and CD16^+^ monocytes but two of the clusters map to CD14^+^ monocytes. (E) One-SENSE plot depicting protein expression heatmap along the x-axis and transcript expression heatmap of some of the top differentially expressed genes along the y-axis. Red box and arrows highlight the differentially expressed genes between cluster 0 and 1. (F) Violin plots showing key genes of the respective myeloid population (top panel) and differentially expressed genes between cluster 0 and 1, suggesting the presence of an inflammatory subpopulation within CD14^+^ CD16^‒^ monocytes that expresses high levels of IL1B, TNF, CXCL3, and CCL4.

Next, we wanted to determine whether the targeted transcriptomics approach can also be used for other immune populations that are not as well studied as T cells. During the past decade, it has become evident that the myeloid cell compartment is complex in terms of cellular heterogeneity (Guilliams et al., 2014; See et al., 2017; Villani et al., 2017) and that commonly used bone-marrow-derived differentiation protocols do not faithfully capture the phenotype of myeloid cells *in vivo* (Guilliams and Malissen, 2016; Helft et al., 2015). Thus, we tested how well targeted transcriptomics could dissect the heterogeneity of the peripheral myeloid compartment. Unbiased clustering using transcripts suggested the presence of 5 different populations (Figure 5A), with clear separation of CD14 and CD16 protein expression (Figure 5B). As expected, visualization of the top differentially expressed genes (Figure 5C) as well as key surface proteins (Figure 5D) mapped these clusters to CD123^+^ plasmacytoid DCs (pDCs), CD1c^+^ cDCs, CD16^+^ monocytes, and CD14^+^ monocytes. We used One-SENSE to further explore the relationship between cluster 0 and 1, revealing that these two populations were very similar in terms of surface protein profile (CD14^+^ CD16^—^ classical monocytes) but were separated by a specific set of transcripts encoding for pro-inflammatory cytokines and chemokines (Figure 5E). We used MAST to confirm that these transcripts were differentially expressed (Finak et al., 2015), with higher expression of CXCL3 and CCL4 (also known as MIP-1b, a chemoattractant for natural killer cells) in cluster 1 (Figure 5F). These cells in cluster 1 could be related to a very recently defined inflammatory subset of CD14^+^ CD163^+^ myeloid cells (Dutertre et al., 2019). Thus, combining protein and transcriptome data allowed us to observe multiple functional subsets within the peripheral CD14^+^ myeloid population, which were not apparent by surface marker expression alone. In summary, these data highlight that targeted transcriptomics can be used for exploratory studies of different immune compartments.

## DISCUSSION

Current efforts in the field of single-cell analysis focus on the integrative measurement of multiple modalities per cell. Ultimately, being able to analyze DNA sequence, genome accessibility status, transcript, regulatory RNAs, and protein expression all together would allow a holistic understanding of cellular function, but this has not yet been achieved (Stuart and Satija, 2019). Arguably one of the most important steps on this trajectory has been the ability to combine protein and transcript measurements by sequencing at the single-cell level by using high-throughput methods (Peterson et al., 2017; Stoeckius et al., 2017). However, with increased cell numbers, these combined measurements can quickly become resource intensive, mostly due to the high number of sequencing reads that are required per cell. Moreover, to fully leverage the advantage of multi-omic single-cell analysis approaches, it is imperative to collect large cell numbers to adequately represent low-abundance cellular populations, such as antigen-specific T cells or antigen-presenting cells (APCs). This can be exemplified with DC subsets; CD1c^+^ cDCs typically represent only 0.1%–0.5% of cells in the peripheral blood, i.e., to capture 50 cells without prior enrichment requires sequencing a minimum of 10,000 cells. However, even 50 cells might be insufficient to extrapolate functional potential if there is pronounced heterogeneity within such a rare population.

The targeted transcriptomic approach that we describe here provides an alternative platform that significantly lowers the number of reads required for sequencing saturation of transcripts compared to WTA approaches but still provides valuable information on 492 immune-centric genes. Notably, the targeted workflow avoids the significant number of reads used by transcripts encoding ribosomal proteins, which provide limited insight in the context of specifically assessing immune cell function. In combination with the lower number of target genes relative to WTA, this contributes to requiring less reads to achieve library saturation. It is important to keep in mind that a targeted approach sacrifices the unbiased nature and breadth of WTA. However, many immunological applications are centered around a set of critical immune effector molecules, such as cytokines, chemokines, or transcription factors, which are comprehensively covered by a targeted gene panel. Because genes of interest can be selected for such a panel, this approach allows tailoring toward a specific research question.

Our experiments suggest that a targeted workflow can, in some cases, deliver high sensitivity when it comes to detecting relatively low abundance transcripts, but we also found a set of genes that was underrepresented relative to WTA. This is probably related to the sub-optimal amplification efficiencies of certain targeted primer pairs. Modifying primer design should be sufficient to improve detection sensitivity of these genes. Overall, in many experimental setups it might be beneficial to combine both approaches: first, use a WTA platform to identify potentially unknown transcripts and then use a targeted approach (ideally tailored toward gene sets of interest) for profiling larger cell numbers or interrogating cellular responses to specific stimuli. Here, we provide proof-of-concept data that as early as 1 h after stimulation CD8^+^ T cells show heterogeneous patterns of chemokine expression. Comprehensive chemokine and cytokine profiling of T cells after a very short stimulus could be very valuable to gain additional insights into their function, e.g., in the context of cancer immunotherapy (Nagarsheth et al., 2017).

The rather low number of reads per cell required for targeted transcriptomics makes the approach very suitable for combined profiling of transcript and protein expression for larger numbers of cells. This is particularly relevant in the context of T cell biology, where well established T cell subsets, such as memory T cells and Tregs can be difficult to identify in some droplet-based scRNA-seq studies solely on the basis of transcripts (Zheng et al., 2017). This has been attributed to the fact that lymphocytes contain a relatively low amount of mRNA, which, in combination with the inherent drop-out rate of scRNA-seq protocols, fails to detect some low-abundance transcripts that define these cellular subsets (Stuart and Satija, 2019). This issue can be alleviated by measuring surface protein markers, such as the splice variants CD45RA and CD45RO, which have been well studied in the context of naive and memory T cells, or the interleukin-2 (IL-2) receptor alpha chain (CD25) and IL-7 receptor (CD127) for the distinction of Tregs.

In addition, the parallel measurement of surface protein phenotypes allows linking novel cellular clusters (that are defined solely by transcript) with previously characterized cellular subsets that are defined by surface protein phenotype only. Finally, the combined measurement approach can be useful to identify targets with a significant disconnect between transcript and protein expression, as we observed for CD69.

The development of novel technologies can sometimes outpace our ability to validate platforms and reagents. Given that typical single-cell sequencing experiments require complex pre-processing steps and are often visualized using dimensionality reduction techniques, such as UMAP or t-SNE, there is a disconnect between the actual raw data and the interpretation of final heatmaps. Although this might be less of a problem for transcript counts, antibody-based probes require careful validation, which is highlighted by our observation that even the same antibody clone can yield a different result in a multi-omic sequencing experiment relative to conventional cytometry. In general, we found that sequencing-based expression patterns for proteins that show clear bimodal expression (such as CD3, CD14, or other lineage markers) were easy to interpret, but making a positivity call could become challenging for protein markers with low or continuous expression patterns (such as PD1, Tim-3, or IL-7Rα). Distinguishing signal from noise for proteins with rather low expression levels is further complicated by the fact that antibody-based probes inevitably yield some degree of unspecific binding, thus introducing a background signal. As sequencing-based protein measurements become more common, it is important to reach a consensus in the field regarding standard quality-control practices. Reagents need to be carefully tested and validated, preferably with parallel deposition of the validation data in public databases, and analysis approaches should rely on algorithm-assisted determination of positivity cutoffs, e.g., by using SCAMP (Greene et al., 2018) or other tools developed for this purpose.

Additional work is needed to assess whether more complex antibody panels require different sequencing depths relative to our study. We are not providing a general recommendation regarding an optimal sequencing depth to measure protein expression because the required read depth will depend on the oligo-to-antibody ratio of a given reagent and the relative abundance of the target epitopes in an experimental sample. Specifically, samples that are stained with antibody-oligo conjugates that consume a large fraction of the sequencing reads (such as, e.g., HLA-DR, CD4, and CD8) may require additional read depth to ensure that low-abundance protein targets are not missed due to a lack of reads. Finally, it is important to note that sequencing-based antibody measurements are only semiquantitative and cannot be used to determine absolute surface protein counts, which is, in part, due to the limited efficiency of current single-cell platforms regarding oligonucleotide capture and subsequent cDNA synthesis (Zheng et al., 2017).

Ultimately, to advance our understanding of biology, the field relies on innovative approaches to analyze and visualize high-dimensional data (Butler et al., 2018; Cao et al., 2019; Stuart and Satija, 2019). Due to the complexity of the data and different expression scales, this presents a challenge for combined protein-transcript datasets. To alleviate this problem, we have adopted an analysis approach successfully used for high-dimensional cytometry data, One-SENSE (Cheng et al., 2016). By visualizing the top differentially expressed genes in one dimension relative to the measured protein phenotypes, this method allows easy dissection of cells that are similar in transcript phenotype but different in protein phenotype and vice versa. This visualization approach will be a useful tool for biologists to explore future multi-omic datasets to extract biological meaning from these complex multi-dimensional data.

## STAR★METHODS

### LEAD CONTACT AND MATERIALS AVAILABILITY

Further information and requests for resources and reagents should be directed to and will be fulfilled by the Lead Contact, Martin Prlic, at mprlic@fredhutch.org. This study did not generate new unique reagents.

### EXPERIMENTAL MODEL AND SUBJECT DETAILS

#### Primary Cells

Peripheral blood mononuclear cells (PBMCs) were obtained as cryopreserved samples from healthy controls (Seattle Area Control Cohort) via the HIV Vaccine Trial network (HVTN). Researchers authored on this manuscript did not have access to any patient information. Vials with cryopreserved cells were thawed at 37^○^C until a tiny ice crystal was left in the tube, and then carefully diluted in 1mL of pre-warmed complete RPMI (RPMI (GIBCO, #18875119) with 10% FBS (Nucleus Biologics, #AU FBS-500ml L1 HI) and 1% Penicillin-Streptomycin (GIBCO, #15140122) and transferred to a new tube. An additional 13 mL of pre-warmed complete RPMI were added drop by drop, followed by centrifugation for 5 minutes at 400 g and resuspension in 1 mL of complete RPMI.

### METHOD DETAILS

#### T cell stimulation assay

Freshly thawed PBMCs (Seattle Area Control Cohort) were depleted of myeloid cells, B cells and IL3Ra-expressing cells using magnetic-activated cell sorting (MACS) with antibodies targeting CD33, CD20 and CD123 and anti-mouse IgG microbeads (Miltenyi #130-048-402). The purified cell fraction was washed and stimulated in RPMI with 10% FBS with PMA (10 ng/mL) and Ionomycin (1 µg/mL) for 60 minutes at 37^○^C. Unstimulated cells were incubated without PMA/Ionomycin for 60 minutes at 37^○^C. After that, cells were stained with AbSeq antibody-oligo-conjugates targeting CD3, TCRαβ, CD4, CD8, PD1, CD137, CD103, CD69, CD39, CCR7, CD45RA, CD45RO and Tim3, strictly following the manufacturers protocol (BD Biosciences) and subjected to the targeted transcriptomic workflow described below, using the BD Rhapsody T Cell Expression Panel Hs (BD Biosciences, #633751).

#### Flow Cytometry and Cell sorting

For flow cytometric analysis good practices were followed as outlined in the guidelines for use of flow cytometry (Cossarizza et al., 2019). Following thawing, PBMCs were incubated with Fc-blocking reagent (BioLegend Trustain FcX, #422302) and fixable UV Blue Live/Dead reagent (ThermoFisher, #L34961) in PBS (GIBCO, #14190250) for 15 minutes at room temperature. If required, cells were stained with an EBV-Tetramer reagent (peptide YVLDHLIVV; Fred Hutch Immune Monitoring Core) diluted in FACS buffer (PBS with 2% FBS, Nucleus Biologics, #AU FBS-500ml L1 HI) for 30 minutes at room temperature, followed by two washes. After this, cells were incubated for 20 minutes at room temperature with 100 µL total volume of antibody master mix freshly prepared in Brilliant staining buffer (BD Bioscience, #563794), followed by two washes. All antibodies were titrated and used at optimal dilution, and staining procedures were performed in 96-well round-bottom plates. A detailed list of panels used, including fluorochromes and final dilutions of all antibodies are listed in Table S4. Stained cells were fixed with 4% PFA for 20 minutes at room temperature, washed, resuspended in FACS buffer and stored at 4^○^C in the dark until acquisition.

All samples were acquired using a FACSymphony A5 (BD Biosciences), equipped with 30 detectors and 355nm, 405nm, 488nm, 532nm and 628nm lasers and FACSDiva (BD Biosciences). Detector gains were optimized using a modified voltage titration approach (Perfetto et al., 2012) and standardized from day to day using 6-peak Ultra Rainbow Beads (Spherotec, # URCP-38-2K). Single-stained controls were prepared with every experiment using antibody capture beads diluted in FACS buffer (BD Biosciences anti-mouse, #552843 and anti-rat, #552844), or cells for Live/Dead reagent. After acquisition, data was exported in FCS 3.1 format and analyzed using FlowJo (version 10.5.x, BD Biosciences). Doublets were excluded by FSC-A versus FSC-H gating. For some of the plots, the number of acquired cells was down-sampled using the appropriate FlowJo plugin to match the number of cells analyzed in the AbSeq workflow.

All cell sorting was performed on a FACSAria III (BD Biosciences), equipped with 20 detectors and 405nm, 488nm, 532nm and 628nm lasers. For all sorts, an 85 mm nozzle operated at 45 psi sheath pressure was used. Cells were sorted into chilled Eppendorf tubes containing 500 µL of complete RPMI, washed once in PBS and immediately used for subsequent processing.

#### Targeted Transcriptome and protein single-cell library preparation and Sequencing

For all targeted transcriptomics experiments, we utilized the BD Rhapsody Express system, which is based on Fan et al. (2015) with some adaptations prior to the commercial release by BD Biosciences. CD45^+^ live PBMCs and EBV-tetramer^+^ CD8^+^ T cells were sequentially labeled using Single Cell Labeling with the BD Single-Cell Multiplexing Kit (BD Biosciences, #633781) and BD AbSeq Ab-Oligos reagents strictly following the manufacturers protocol (BD Biosciences). Briefly, cells from each donor or subtype of cells (after sorting) were labeled with sample tags (Stoeckius et al., 2018). Each sample was washed twice with FACS buffer before pooling all samples together. Pooled samples were washed one more time and then stained in a total volume of 200 µL of FACS buffer with AbSeq Ab-Oligos (BD Biosciences) diluted as listed in Table S4. The pooled sample was then washed twice, counted and resuspended in cold BD Sample Buffer (BD Biosciences) to achieve approximately 20,000 cells in 620 µL. Single cells from the pooled sample were isolated using Single Cell Capture and cDNA Synthesis with the BD Rhapsody Express Single-Cell Analysis System following the manufacturers protocol (BD Biosciences). After priming the nanowell cartridges, the pooled sample was loaded onto two BD Rhapsody cartridges and incubated at room temperature. Cell Capture Beads (BD Biosciences) were prepared and then loaded onto the cartridge and incubated prior to shaking at 1,000 rpm at room temperature for 15 s on a ThermoMixer C (Eppendorf). According to the manufacturers protocol, cartridges were washed, cells were lysed, and Cell Capture Beads were retrieved and washed prior to performing reverse transcription and treatment with Exonuclease I. cDNA Libraries were prepared using mRNA Targeted, Sample Tag, and BD AbSeq Library Preparation with the BD Rhapsody Targeted mRNA and AbSeq Amplification and BD Single-Cell Multiplexing Kits and protocol (BD Biosciences). In brief, cDNA underwent targeted amplification using the Human Immune Response Panel primers and a custom supplemental panel (all 492 targets are listed in Table S1) via PCR (10 cycles). PCR products were purified, and mRNA PCR products were separated from sample tag and AbSeq products with double-sided size selection using SPRIselect magnetic beads (Beckman Coulter, #B23318). mRNA and Sample Tag products were further amplified using PCR (10 cycles). PCR products were then purified using SPRIselect magnetic beads. Quality and quantity of PCR products were determined by using an Agilent 2200 TapeStation with High Sensitivity D5000 ScreenTape (Agilent) in the Fred Hutch Genomics Shared Resource laboratory. Targeted mRNA product was diluted to 2.5 ng/µL and sample tag and AbSeq PCR products were diluted to 1 ng/µL to prepare final libraries. Final libraries were indexed using PCR (6 cycles). Index PCR products were purified using SPRIselect magnetic beads. Quality of final libraries was assessed by using Agilent 2200 TapeStation with High Sensitivity D5000 ScreenTape and quantified using a Qubit Fluorometer using the Qubit dsDNA HS Kit (ThermoFisher, #Q32854). Final libraries were diluted to 2 nM and multiplexed for paired-end (150bp) sequencing on a HiSeq 2500 sequencer (Illumina). The final mean read depths for the three experiments that we sequenced were as follows: Experiment 1 (used in Figure S3): 18,445 reads/cell for AbSeq library (saturation 88.4%), 9,137 reads/cell for mRNA library (saturation 86.7%). Experiment 2 (main figures): 4,330 reads/cell for AbSeq library (saturation 53.3%), 2,165 reads/cell for mRNA library (saturation 89.9%). Experiment 3 (used for Figure 4; T cell stimulation assay): 19,236 reads/cell for the AbSeq library (saturation 94.7%), 14,042 reads/cell for the mRNA library (saturation 94.9%).

#### Whole Transcriptome single-cell library preparation and sequencing

cDNA libraries of CD45^+^ Live PBMCs were generated using the Chromium Single Cell 3ʹ Reagent Kits v2 (10x Genomics) protocol targeting 5,000 cells in two separate wells. Briefly, single cells were isolated into oil emulsion droplets with barcoded gel beads and reverse transcriptase mix. cDNA was generated within these droplets, then the droplets were dissociated. cDNA was purified using DynaBeads MyOne Silane magnetic beads (ThermoFisher, #370002D). cDNA amplification was performed by PCR (10 cycles) using reagents within the Chromium Single Cell 3ʹ Reagent Kit v2 (10x Genomics). Amplified cDNA was purified using SPRIselect magnetic beads (Beckman Coulter). cDNA was enzymatically fragmented and size selected prior to library construction. Libraries were constructed by performing end repair, A-tailing, adaptor ligation, and PCR (12 cycles). Quality of the libraries was assessed by using Agilent 2200 TapeStation with High Sensitivity D5000 ScreenTape (Agilent). Quantity of libraries was assessed by performing digital droplet PCR (ddPCR) with Library Quantification Kit for Illumina TruSeq (BioRad, #1863040). Libraries were diluted to 2 nM and paired-end sequencing was performed on a HiSeq 2500 sequencer (Illumina). The final read depths for the two technical replicates that we sequenced were 77,049 reads/cell and 86,246 reads/cell, respectively.

### QUANTIFICATION AND STATISTICAL ANALYSIS

#### Cell Ranger processing for WTA data

Raw base call (BCL) files were demultiplexed to generate Fastq files using the cellranger mkfastq pipeline within Cell Ranger 2.1.1 (10x Genomics). Targeted transcriptome Fastqs were further analyzed via Seven Bridges (BD Biosciences). Whole transcriptome Fastq files were processed using the standard cellranger pipeline (10x genomics) within Cell Ranger 2.1.1. Briefly, cellranger count performs alignment, filtering, barcode counting, and UMI counting. The cellranger count output was fed into the cellranger aggr pipeline to normalize sequencing depth between samples. The final output of cellranger (molecule per cell matrix) was then analyzed in R using the package Seurat (version 2.3 and 3.0) as described below.

#### Seven Bridges processing for targeted transcriptomics data

Targeted transcriptomics Fastq files were processed via the standard Rhapsody analysis pipeline (BD Biosciences) on Seven Bridges (https://www.sevenbridges.com) per the manufacturérs recommendations. First, R1 and R2 reads are filtered for high-quality reads, dropping reads that are too short (less than 64 bases for R2) or have a base quality score of less than 20. Then, R1 reads are annotated to identify cell label sequences and unique molecular identifiers (UMIs), and R2 reads are mapped to the respective reference sequences using Bowtie2. Finally, all valid R1 and R2 reads are combined and annotated to the respective molecules. For all of our analysis, we utilized recursive substation error correction (RSEC) as well as distribution-based error correction (DBEC), which are manufacturer-developed algorithms correcting for PCR and sequencing errors. For determining putative cells (which will contain many more reads than noise cell labels), a filtering algorithm takes the number of DBEC-corrected reads into account, calculating the minimum second derivative along the cumulative reads as the cut-off point. Final expression matrices contain DBEC-adjusted molecule counts in a CSV format. For sample tag assignment, a cell is called as a singlet if the minimum read count for a given sample tag is reached, and more than 75% of the sample tag reads are derived from a single sample tag antibody. In turn, if the count for two more sample tag antibodies exceeds the minimum thresholds, these cells are labeled as multiplets, and if a cell does not reach criteria for either a multiplet or singlet, it is labeled as undetermined. Both multiplets and undetermined cells were excluded from analysis as described below.

For further analysis, molecule count tables were read into the R package Seurat (version 2.3 and 3.0) using customized scripts and analyzed as described below.

#### Seurat workflow for targeted and WTA data

The R package Seurat (Butler et al., 2018) was utilized for all downstream analysis. For whole transcriptome data, based on commonly used cutoffs suggested by Butler et al., only cells that had at least 200 genes (with % 20% being mitochondrial genes) were included in analysis (removing 182 out of a total of 5,416 cells). A natural log normalization using a scale factor of 10,000 was performed across the library for each cell. UMIs and mitochondrial genes (only for WTA data) were linearly scaled to remove these variables as unwanted sources of variation. For the targeted data, singlets were identified during pre-processing as described above. In our case, out of 29,033 total cells, 1,532 were called as doublets (and 243 events as undetermined), with 27,258 cells remaining (donor 1: 8,984 cells, donor 2: 8,956 cells, donor 3: 9,318 cells). For WTA data, doublets and low-quality cells were identified by their outlier UMI and gene counts (more than 15,000 UMIs and more than 3,000 genes), and their high percentage of mitochondrial genes (more than 20%), removing 640 cells.

For WTA, dimensionality reduction using UMAP and clustering was performed on a subset of variable genes. For targeted transcriptomics, no gene per cell cutoffs were imposed and the data was normalized with the same method (natural log normalization using a scale factor of 10,000). To make sure that the same normalization methods could be used, we compared the library size factor distribution (using the package scater) between the WTA and targeted transcriptomic data, which showed similar distributions irrespective of whether using all genes or only the targeted gene set (however, further comparative experiments might be required to ensure that the standard log normalization implemented in Seurat is an effective normalization method for different targeted transcriptomic experimental setups). When scaling data, UMI was the only regressed variable. Dimensionality reduction using UMAP and clustering was based on either all genes or all proteins. For differential gene expression analysis we utilized the Seurat implementation of MAST (model-based analysis of single-cell transcriptomes) with the number of UMIs included as a covariate (proxy for cellular detection rate (CDR)) in the model (Finak et al., 2015).

For generation of some FCS files the antibody molecule count tables were converted using the R packages premessa and flow-Core. FCS-files with antibody molecule count signals were analyzed in FlowJo 10.5.x (BD Biosciences) using either an arcsinh transformation or biexponential transformation.

#### Data processing for One-SENSE and generation of FCS files

CSV files of raw counts were converted to FCS files using a script adapted from https://gist.github.com/yannabraham/c1f9de9b23fb94105ca5. Raw counts were normalized based on total counts per cell, then scaled to a value of 10,000 based on the Seurat normalization algorithm. A natural log transformation was applied to gene expression data, while protein expression data was randomized by adding a random uniform distribution from 0 to 1, followed by transformation with the function arcsinh(x/5). Dimensionality reduction using UMAP was performed separately on all genes and proteins to reduce them to one dimension before plotting. Cells were also split into 500 bins of equivalent width based on one-dimensional UMAP data, then used to generate heatmaps colored by median marker intensity per bin (low expression: blue, mid expression: green, high expression: red). Heatmaps corresponding to empty areas of the One-SENSE plot were colored in black. An example script for One-SENSE processing can be found at https://github.com/MairFlo/Targeted_transcriptomics.

### DATA AND CODE AVAILABILITY

The sequencing data discussed in this publication have been deposited in the NCBI’s Gene Expression Omnibus (Edgar et al., 2002) and are accessible through GEO series accession number GEO: GSE135325. (https://www.ncbi.nlm.nih.gov/geo/query/acc.cgi?acc=GSE135325). The accession number for all flow cytometry data reported in this paper is FlowRepository: FR-FCM-Z266 (http://flowrepository.org/id/FR-FCM-Z266). All scripts used for data processing and plot generation are available at https://github.com/MairFlo/Targeted_transcriptomics.

## Supplementary Material

1

2

## Figures and Tables

**Table:** KEY RESOURCES TABLE

REAGENT or RESOURCE	SOURCE	IDENTIFIER
Antibodies		
CD183 (CXCR3)-BUV395 (clone 1C6)	BD Biosciences	Cat#565223; RRID:AB_2687488
CD3-BUV496 (clone UCHT1)	BD Biosciences	Cat#564809; RRID:AB_2744388
CD25-BUV563 (clone 2A3)	BD Biosciences	Cat#565699; RRID:AB_2744341
HLA-DR-BUV661 (cloneG46-6)	BD Biosciences	Cat#565073; RRID:AB_2722500
ICOS-BUV737 (clone DX29)	BD Biosciences	Cat#564778; RRID:AB_2738947
CD8-BUV805 (clone SK1)	BD Biosciences	Cat#564912; RRID:AB_2744465
CD196 (CCR6)-BV421 (clone 11A9)	BD Biosciences	Cat#562724; RRID:AB_2737747
TCRgd-BV480 (clone DX12)	BD Biosciences	Cat#566084; RRID:AB_2739495
CD14-BV570 (clone M5E2)	BioLegend	Cat#301832; RRID:AB_2563629
PD1-BV605 (clone EH12.1)	BD Biosciences	Cat#563245; RRID:AB_2738091
CD69-BV650 (clone FN50)	BD Biosciences	Cat#563835; RRID:AB_2738442
CD45RA-BV711 (clone UCHL1)	BD Biosciences	Cat#564675; RRID:AB_2738885
CD103-BV750 (clone Ber-ACT8)	BD Biosciences, custom	Cat#624380; no RRID
CD127-BV785 (HIL-7R-M21)	BD Biosciences	Cat#563324; RRID:AB_2738138
Tim3-BB515 (clone 7D3)	BD Biosciences	Cat#565569; RRID:AB_2744368
CD16-BB630 (clone 3G8	BD Biosciences, custom	Cat#624294; no RRID
CD27-BB660 (clone M-T271)	BD Biosciences, custom	Cat#624295; no RRID
CD161-BB700 (clone DX12)	BD Biosciences	Cat#745791; RRID:AB_2743247
CD38-BB790 (clone HIT2)	BD Biosciences, custom	Cat#624296; no RRID
CD194 (CCR4)-PE (clone 1G1)	BD Biosciences	Cat#551120; RRID:AB_394054
CD39-PECF594 (TU66)	BD Biosciences	Cat#563678; RRID:AB_2738367
CD137-PECy5 (clone 4B4-1)	BD Biosciences	Cat#551137; RRID:AB_394067
CD19-PE-Cy5.5 (clone SJ25-C1)	Thermo Fisher	Cat#MHCD1918; RRID:AB_10373840
CD197 (CCR7)-PECy7 (clone 3D12)	BD Biosciences	Cat#557648; RRID:AB_396765
EBV-Tetramer-APC	Fred Hutchinson Immune monitoring core	peptide YVLDHLIVV
CD45RO-AF700 (clone UCHL1)	BioLegend	Cat#304218; RRID:AB_493765
CD4-APCH7 (clone RPA-T4)	BD Biosciences	Cat#560158; RRID:AB_1645478
CD40-BUV395 (clone 5C3)	BD Biosciences	Cat#565202; RRID:AB_2739110
CD56-BUV563 (clone NCAM16.2)	BD Biosciences	Cat#565704; RRID:AB_2744431
CD86-BUV737 (clone FUN-1)	BD Biosciences	Cat#564428; RRID:AB_2738804
CX3CR1-BV421 (clone 2A9-1)	BD Biosciences	Cat#565800; no RRID
CD28-BV480 (clone CD28.2)	BD Biosciences	Cat#566110; RRID:AB_2739512
CD141-BV605 (clone 1A4)	BD Biosciences	Cat#740421; RRID:AB_2740151
Sirpa-BV650 (clone SE5A5)	BD Biosciences	Cat#743565; no RRID
OX40-BV711 (clone ACT-35)	BioLegend	Cat#350029; RRID:AB_2632863
CD11b-BV750 (clone ICRF44)	BD Biosciences, custom	Cat#624380; no RRID
CD123-BV786 (clone 7G3)	BD Biosciences	Cat#564196; RRID:AB_2738662
CD206-BB515 (clone 19.2)	BD Biosciences	Cat#564668; RRID:AB_2738882
CD32-BB700 (clone FLI8.26)	BD Biosciences	Cat#742216; no RRID
Lag3-PE (clone T47-530)	BD Biosciences	Cat#565617; no RRID
CD163-PECF594 (clone GHI/61)	BD Biosciences	Cat#562670; RRID:AB_2737711
CD80-PECy5 (clone L307.4)	BD Biosciences	Cat#559370; RRID:AB_397239
CD4-PECy7 (clone SK3)	BD Biosciences	Cat#557852; RRID:AB_396897
CD1c-AF647 (clone F10/21A3)	BD Biosciences	Cat#565048; RRID:AB_2744318
CD11c-AF700 (clone B-ly6)	BD Biosciences	Cat#561352; RRID:AB_10612006
HLA-DR-APCH7 (clone L243)	BD Biosciences	Cat#561358; RRID:AB_10611876
UV Fixable Live-Dead	Thermo Fisher	Cat#L34961; no RRID
Human TruStain FcX (Fc-Block)	BioLegend	Cat#422302; no RRID
Cytofix/CytoPerm	BD Biosciences	Cat#554722; no RRID
CD3-Ab-O (clone SK7)	BD Biosciences	AHS0033; no RRID
CD4-Ab-O (clone SK3)	BD Biosciences	AHS0032; no RRID
CD8-Ab-O (clone RPA-T8)	BD Biosciences	AHS0027; no RRID
CD19-Ab-O (clone SJ25C1)	BD Biosciences	AHS0030; no RRID
CD14-Ab-O (clone MPHIP9)	BD Biosciences	AHS0037; no RRID
CD16-Ab-O (clone 3G8)	BD Biosciences	AHS0053; no RRID
CD56-Ab-O (clone NCAM16.2)	BD Biosciences	AHS0019; no RRID
CD11b-Ab-O (clone M1/70)	BD Biosciences	AHS0005; no RRID
CD25-Ab-O (clone 2A3)	BD Biosciences	AHS0026; no RRID
HLA-DR-Ab-O (cloneG46-6)	BD Biosciences	AHS0035; no RRID
CD45RA-Ab-O (clone HI100)	BD Biosciences	AHS0009; no RRID
CD127-Ab-O (clone HIL-7R-M21)	BD Biosciences	AHS0028; no RRID
CD38-Ab-O (clone HIT2)	BD Biosciences	AHS0022; no RRID
CD197-Ab-O (clone 3D12)	BD Biosciences	AHS0007; no RRID
CD279-Ab-O (clone EH12.1)	BD Biosciences	AHS0014; no RRID
CD28-Ab-O (clone CD28.2)	BD Biosciences	AHS0024; no RRID
CD27-Ab-O (clone M-T271)	BD Biosciences	AHS0025; no RRID
CD69-Ab-O (clone FN50)	BD Biosciences	AHS0010; no RRID
CD123-Ab-O (clone 7G3)	BD Biosciences	AHS0020; no RRID
CD45RO-Ab-O (clone UCHL1)	BD Biosciences	AHS0036; no RRID
CD11c-Ab-O (clone B-Ly6)	BD Biosciences	AHS0056; no RRID
CD86-Ab-O (clone FUN-1)	BD Biosciences	AHS0057; no RRID
CD183-Ab-O (clone 1C6)	BD Biosciences	AHS0031; no RRID
CD196-Ab-O (clone 11A9)	BD Biosciences	AHS0034; no RRID
CD80-Ab-O (clone L307.4)	BD Biosciences	AHS0046; no RRID
CD278-Ab-O (clone DX29)	BD Biosciences	AHS0012; no RRID
CD194-Ab-O (clone 1G1)	BD Biosciences	AHS0038; no RRID
CD40-Ab-O (clone 5C2)	BD Biosciences	AHS0117; no RRID
CD137-Ab-O (clone 4B4-1)	BD Biosciences	AHS0003; no RRID
TCRgd-Ab-O (clone B1)	BD Biosciences	AHS0015; no RRID
CD163-Ab-O (clone GH1/61)	BD Biosciences	AHS0062; no RRID
CD134-Ab-O (clone ACT35)	BD Biosciences	AHS0013; no RRID
Tim3-Ab-O (clone 7D3)	BD Biosciences	AHS0016; no RRID
CD103-Ab-O (clone Ber-ACT8)	BD Biosciences	AHS0016; no RRID
CD206-Ab-O (clone 19.2)	BD Biosciences	AHS0072; no RRID
CD32-Ab-O (clone FLI8.26)	BD Biosciences	AHS0073; no RRID
CD161-Ab-O (clone DX12)	BD Biosciences	AHS0002; no RRID
CD39-Ab-O (clone TU66)	BD Biosciences	AHS0006; no RRID
CD141-Ab-O (clone 1A4)	BD Biosciences	AHS0083; no RRID
Lag3-Ab-O (clone T47-530)	BD Biosciences	AHS0018; no RRID
CD1c-Ab-O (clone F10/21A3)	BD Biosciences	AHS0088; no RRID
Biological Samples		
Cryopreserved peripheral blood mononuclear cells	HVTN, Fred Hutch	NA
Critical Commercial Assays		
Rhapsody AbSeq reagent pack (4 reactions)	BD Biosciences	Cat#633771
Rhapsody Human T cell expression panel	BD Biosciences	Cat#633751
Rhapsody Human Immune Response panel	BD Biosciences	Cat#633750
Rhapsody custom gene panel (see Table S1)	BD Biosciences	Cat#633743
Human Single cell multiplexing kit	BD Biosciences	Cat#633781
Deposited Data		
Flow Cytometry Data	http://www.flowrepository.org	FR-FCM-Z266
sc-RNaseq/AbSeq Data	https://www.ncbi.nlm.nih.gov/geo/	GEO: GSE135325
Software and Algorithms		
R Studio and R environment	The R project for Statistical Computing	https://rstudio.com/ and https://cran.r-project.org/
Seurat v2.3 and v3.0	Satija Lab, NYU, New York Genome Center	https://github.com/satijalab/seurat
Seven Bridges (standard pre-processing of Rhapsody raw sequencing data, i.e., FASTQ files)	BD Biosciences	https://www.sevenbridges.com
CellRanger (standard pre-processing of WTA raw sequencing data, i.e., FASTQ files)	10x genomics	https://www.10xgenomics.com/
FlowJo 10.5.x (analysis and visualization of flow cytometry and AbSeq data)	BD Biosciences	https://www.flowjo.com
Prism (plotting)	GraphPad	N/A
Illustrator (figure generation)	Adobe	N/A
Seurat workflow for all WTA and targeted trancriptomic single cell molecule count tables	Prlic Lab, FHCRC, Seattle	https://github.com/MairFlo/
One-SENSE (visualization of data proteintranscript)	Newell Lab, FHCRC, Seattle	https://github.com/MairFlo/
Other		
FACSymphony flow cytometer	BD Biosciences	N/A
Rhapsody Express instrument	BD Biosciences	N/A

## Highlights

- Targeted transcriptomics captures immune cell heterogeneity at a low sequencing depth
- Antibody panels for sequencing-based protein measurement require validation
- Combined protein and transcript measurements highlight T cell heterogeneity
- One-SENSE provides an intuitive visualization tool for protein-transcript datasets
