# Supplementary material for: A Targeted Multi-omic Analysis Approach Measures Protein Expression and Low-Abundance Transcripts on the Single-Cell Level
Source: Cell Rep. Author manuscript; Available in PMC 2020 May 14. (PMC7224638; doi:10.1016/j.celrep.2020.03.063)
Supplement: 1 [file NIHMS1582940-supplement-1.pdf]

Cell Reports, Volume *31*

Supplemental Information

A Targeted Multi-omic Analysis Approach Measures Protein Expression and Low-Abundance Transcripts on the Single-Cell Level

Florian Mair, Jami R. Erickson, Valentin Voillet, Yannick Simoni, Timothy Bi, Aaron J. Tyznik, Jody Martin, Raphael Gottardo, Evan W. Newell, and Martin Prlic

Suppl. Figure 1

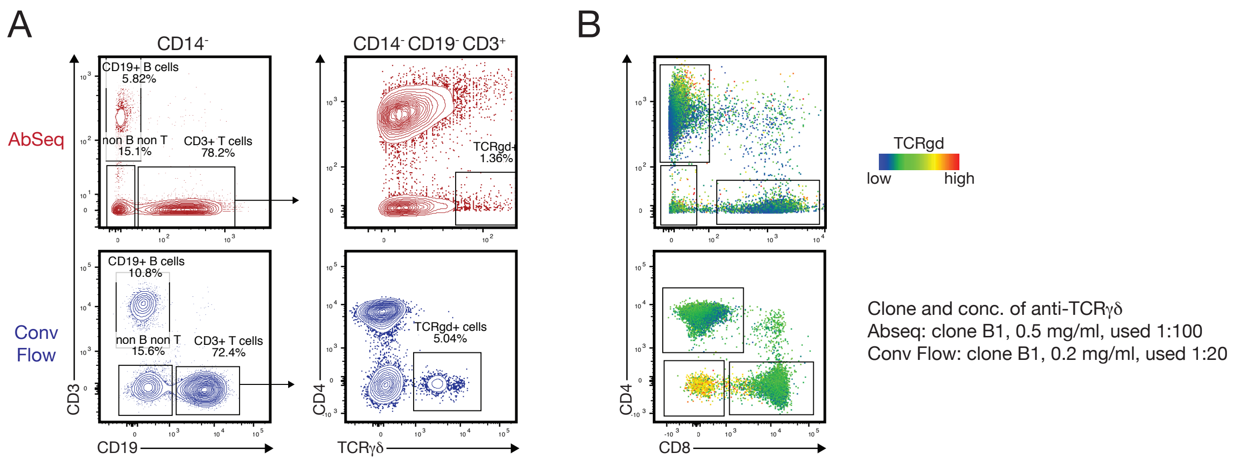

Supplementary figure 1 (related to figure 1): Example for a poorly performing reagent
(A) Manual gating of main immune subsets using the combined AbSeq data set (upper panel, red) and concatenated and downsampled events from the flow cytometry data set (lower panel, blue), highlighting the population of γδ T cells.
(B) Heatmap overlay of the TCRγδ signal on a CD4 vs CD8 plot for the AbSeq data set (upper panel) and flow cytometry data set (lower panel). Antibody stock concentrations and dilutions are listed.

Suppl. Figure 2

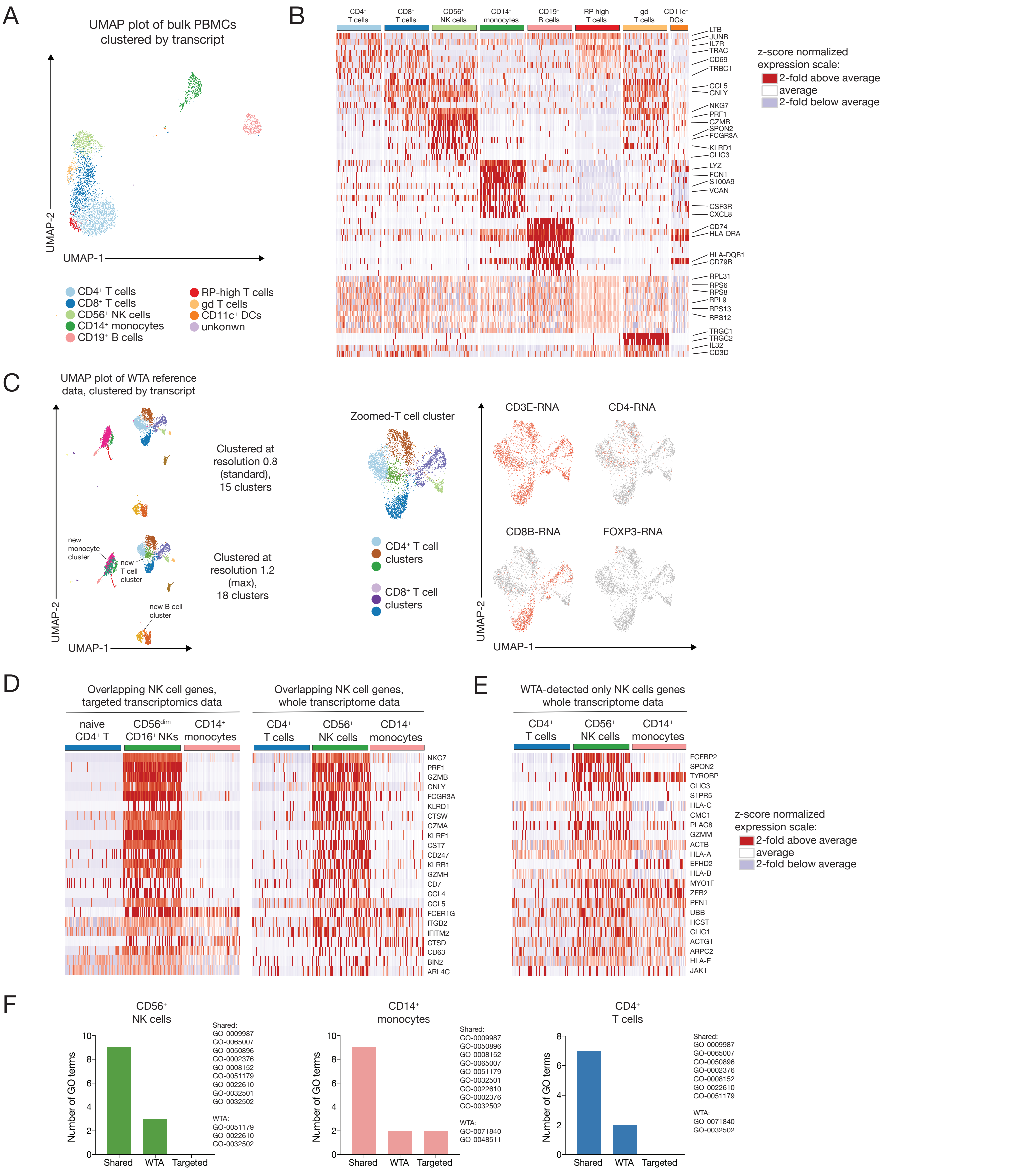

Supplementary figure 2 (related to figure 2): Comparison of targeted transcriptomic and WTA data
(A) Graph-based clustering of WTA data obtained from the same donor as in main Figure 2.
(B) The top 10-differentially expressed genes for each cluster were identified using the Seurat implementation of MAST and visualized on a heatmap after z-score normalization. Cluster names are shown in the same color scheme as in (A).
(C) Graph-based clustering of WTA data from a public whole transcriptome reference data set at two different clustering resolution (left) and the zoomed T cell-population with an heatmap overlay showing CD3E, CD4, CD8B and FOXP expression.
(D) Expression pattern of all genes that were detected as differentially expressed both in WTA and targeted transcriptomic data for the NK cell cluster relative to naive CD4+ T cells and CD14+ monocytes.
(E) Expression pattern of genes that were only detected in the NK cell cluster of the WTA data.
(F) Number of gene ontology (GO)-terms that were shared between both data sets, or only detected in either the WTA or targeted data. GO-Term accession numbers are listed next to the bar graphs.

Suppl. Figure 3

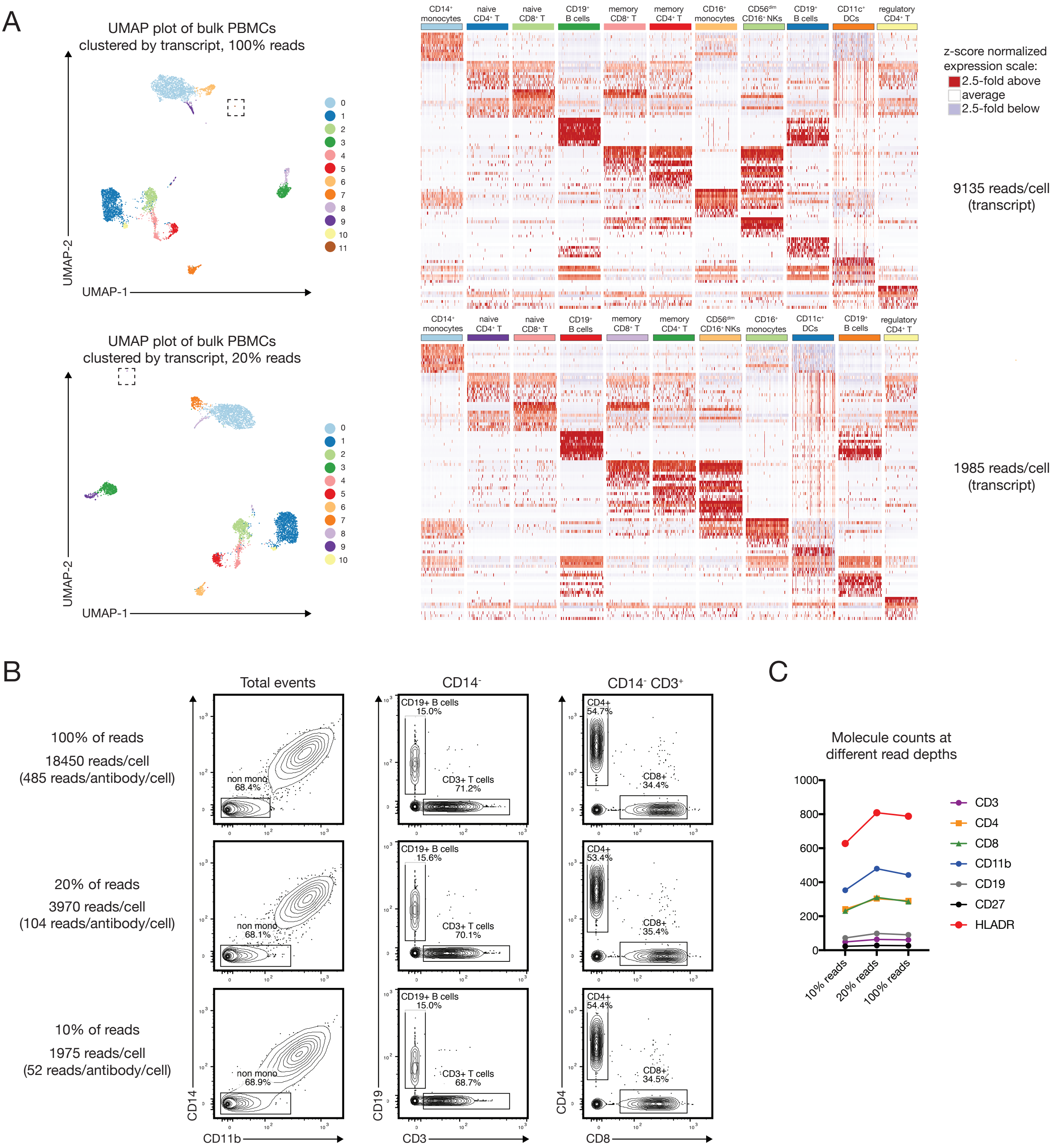

Supplementary figure 3 (related to figure 2): Effect of different read depths on the resolution of targeted transcriptomic and AbSeq data

(A) 5,400 cells from a different donor were sequenced at a total depth of approximately 30,000 reads/cell. Upper panel depicts UMAP plot after graph-based clustering (using transcript) and a heatmap of the top differentially expressed genes at full read depth, lower panel using only 20% of the reads. Read depth per cell for the transcript library is indicated on the right. Squared box on the UMAP plot indicates one cluster that is separated as cluster 11 at full read depth, but pooled with cluster 8 at lower read-depth.

(B) Representative protein signals at the indicated read depths.

(C) Positive cells for the indicated markers (only bimodal populations were selected) were gated, median molecule counts calculated and plotted across the different read depths.

Supplementary Table 1
List of genes for targeted transcriptomics assay, related to main figure 2

Human Immune Response Panel							
1	ADA	100	CEACAM8	200	IGHD_membrane	300	NCAM1
2	ADGRE1	101	CHI3L1	201	IGHE_secreted	301	NCR3
3	ADGRG3	102	CHI3L2	202	IGHG1_membrane	302	NINJ2
4	AIM2	103	CLC	203	IGHG1_secreted	303	NKG7
5	ALAS2	104	CLEC10A	204	IGHG2_secreted	304	NRP1
6	ANXA5	105	CLEC4D	205	IGHG3_secreted	305	NT5E
7	AOC3	106	CLEC4E	206	IGHG4_secreted	306	PASK
8	APOBEC3G	107	CMKLR1	207	IGHM_membrane	307	PAX5
9	APOE	108	CMTM2	208	IGHM_secreted	308	PCNA
10	AQP9	109	CNOT2	209	IGKC	309	PDCD1
11	ARG1	110	CNTNAP3	210	IGLC3	310	PDIA4
12	ARL4C	111	CPA3	211	IKZF1	311	PDIA6
13	ATF6B	112	CR2	212	IKZF2	312	PI3
14	AURKB	113	CSF2	213	IL12A	313	PIK3AP1
15	AZU1	114	CSF3	214	IL12RB1	314	PIK3IP1
16	B3GAT1	115	CST7	215	IL12RB2	315	PMCH
17	BACH2	116	CTLA4	216	IL13	316	POU2AF1
18	BAX	117	CTSD	217	IL15	317	PRDM1
19	BCL11B	118	CTSG	218	IL15RA	318	PRF1
20	BCL2	119	CTSW	219	IL17A	319	PSEN1
21	BCL2A1	120	CX3CR1	220	IL17F	320	PTGDR2
22	BCL6	121	CXCL1	221	IL18	321	PTPRC
23	BIN2	122	CXCL10	222	IL18R1	322	PTTG2
24	BIRC3	123	CXCL11	223	IL18RAP	323	QPCT
25	BLK	124	CXCL13	224	IL1B	324	RGS1
26	BLNK	125	CXCL16	225	IL1R2	325	RNASE2
27	BPI	126	CXCL2	226	IL1RL1	326	RNASE6
28	BTG1	127	CXCL3	227	IL1RN	327	RORA
29	BTLA	128	CXCL5	228	IL2	328	RORC
30	C10orf54	129	CXCL8	229	IL21	329	RPN2
31	C1QA	130	CXCL9	230	IL22	330	RUNX3
32	C1QB	131	CXCR1	231	IL23R	331	S100A10
33	CASP5	132	CXCR2	232	IL25	332	S100A12
34	CBLB	133	CXCR3	233	IL2RA	333	S100A9
35	CCL1	134	CXCR4	234	IL2RB	334	SELL
36	CCL13	135	CXCR5	235	IL3	335	SELPLG
37	CCL17	136	CXCR6	236	IL31	336	SLC25A37
38	CCL19	137	DEFA3	237	IL32	337	SLC7A7
39	CCL2	138	DEFA4	238	IL33	338	SNCA
40	CCL20	139	DOCK8	239	IL3RA	339	SPP1
41	CCL22	140	DPP4	240	IL4	340	STAT1
42	CCL3	141	DUSP1	241	IL4R	341	STAT3
43	CCL4	142	DUSP2	242	IL5	342	STAT4
44	CCL5	143	DUSP4	243	IL6	343	STAT5A
45	CCND2	144	EBF1	244	IL7R	344	STAT6
46	CCR1	145	EGR1	245	IL9	345	TARP_refseq
47	CCR10	146	EGR3	246	IRF4	346	TBX21
48	CCR2	147	ELANE	247	IRF8	347	TCF4
49	CCR3	148	ENTPD1	248	ITGA4	348	TCF7
50	CCR4	149	EOMES	249	ITGAE	349	TCL1A
51	CCR5	150	EPX	250	ITGAM	350	TGFB1
52	CCR7	151	F13A1	251	ITGAX	351	TGFB3
53	CCR8	152	F5	252	ITGB2	352	TGFBI
54	CCR9	153	FAM129C	253	JCHAIN	353	THBD
55	CD14	154	FAM65B	254	JUN	354	THBS1
56	CD160	155	FAS	255	JUNB	355	TIAF1
57	CD163	156	FASLG	256	KCNE3	356	TIGIT
58	CD1A	157	FCER1A	257	KDELR1	357	TLR2
59	CD1B	158	FCER1G	258	KIAA0101	358	TLR7
60	CD1C	159	FCER2	259	KIR2DL1	359	TLR8
61	CD2	160	FCGR3A	260	KIT	360	TLR9
62	CD200	161	FCN1	261	KLRB1	361	TMEM97
63	CD209	162	FLT3	262	KLRC1	362	TNF
64	CD22	163	FN1	263	KLRC3	363	TNFRSF13C
65	CD24	164	FOSB	264	KLRC4	364	TNFRSF17
66	CD244	165	FOSL1	265	KLRF1	365	TNFRSF25
67	CD247	166	FOXO1	266	KLRG1	366	TNFRSF4
68	CD27	167	FOXP1	267	KLRK1	367	TNFRSF8
69	CD274	168	FOXP3	268	LAG3	368	TNFRSF9
70	CD28	169	FTH1	269	LAIR2	369	TNFSF10
71	CD300A	170	FUT4	270	LAMP1	370	TNFSF13
72	CD33	171	FYB	271	LAMP3	371	TNFSF13B
73	CD34	172	FYN	272	LAP3	372	TNFSF14
74	CD36	173	GAB2	273	LAT	373	TNFSF8
75	CD37	174	GAPDH	274	LAT2	374	TOP2A
76	CD38	175	GIMAP2	275	LCK	375	TPSAB1
77	CD3D	176	GIMAP5	276	LEF1	376	TRAC
78	CD3E	177	GNAI2	277	LGALS1	377	TRAT1
79	CD3G	178	GNLY	278	LGALS3	378	TRBC2
80	CD4	179	GZMA	279	LGALS9	379	TRDC
81	CD40	180	GZMB	280	LIF	380	TREM1
82	CD44	181	GZMH	281	LILRB4	381	TRIB2
83	CD48	182	GZMK	282	LIPA	382	TSPAN32
84	CD5	183	HAVCR2	283	LRRC32	383	TXK
85	CD52	184	HLA-A	284	LTA	384	TYMS
86	CD6	185	HLA-DMA	285	LTB	385	UBE2C
87	CD63	186	HLA-DPA1	286	LY86	386	VEGFA
88	CD69	187	HLA-DQB1	287	LYN	387	VMO1
89	CD7	188	HLA-DRA	288	MCM2	388	VNN2
90	CD70	189	HMMR	289	MCM4	389	VPREB3
91	CD72	190	ICAM1	290	MGST1	390	VPS28
92	CD74	191	ICOS	291	MITF	391	VSIG4
93	CD79A	192	IER3	292	MKI67	392	XBP1
94	CD79B	193	IFITM2	293	MME	393	YBX3
95	CD80	194	IFITM3	294	MMP12	394	ZAP70
96	CD86	195	IFNA1	295	MMP9	395	ZBED2
97	CD8A	196	IFNG	296	MS4A1	396	ZBTB16
98	CD8B	197	IFNGR1	297	MYC	397	ZNF683
99	CD9	198	IGBP1	298	MZB1		
		199	IGHA1_secr	299	NAMPT		

Custom Gene Panel	
1	ADAM15
2	ADAM17
3	ADAM28
4	AHR
5	AIF1
6	APOC1
7	AXL
8	BATF
9	BIRC5
10	CA10
11	CAPG
12	CCL3L3
13	CCL4L2
14	CCR6
15	CD207
16	CD273
17	CD40LG
18	CD68
19	CD83
20	CD96
21	CDH1
22	CLU
23	CRTAM
24	CSF1
25	CTSK
26	CTSS
27	NSG1
28	DUSP6
29	FBLN2
30	FBN2
31	FCGR3B
32	FOS
33	FOXO3
34	GATA3
35	GZMM
36	HIF1A
37	HLA-DPB1
38	HLA-DQA1
39	HOPX
40	ICOSLG
41	ID3
42	IFITM1
43	IL10
44	IL22RA2
45	IL23A
46	IL6R
47	IRF7
48	ITGA1
49	ITGA5
50	KCNK5
51	KLF2
52	KLF3
53	KLF6
54	KLRC2
55	KLRD1
56	LTC4S
57	LYZ
58	MFAP2
59	MMP1
60	MMP2
61	MNDA
62	MX1
63	MX2
64	MXRA8
65	NLRP3
66	NPDC1
67	PTGDS
68	PTPN6
69	PXDN
70	RAP1GAP2
71	RhoA
72	S100A8
73	S1PR1
74	SBK1
75	SERPINA1
76	SOCS3
77	SOX13
78	STK38
79	TCF21
80	TIMP1
81	TIMP2
82	TIMP3
83	TIMP4
84	TMEM123
85	TNFRSF18
86	TNFRSF1B
87	TOX
88	TSPAN18
89	TTC16
90	TTYH2
91	VCAN
92	XCL1
93	XCL2
94	ZFP36
95	ZNF219

Supplementary Table 2, related to main figure 2
Relative detection ratio of genes in the targeted transcriptomic assay relative to WTA for one donor
Color coding and order of genes is the same as in main figure 2D

	Raw Counts			Normalized to cell#			
**Gene**	**AbSeq**	**10x**	**ratio**	**AbSeq**	**10x**	**ratio**	**Log Transform**
**LTC4S**	1	190	0.005263	0.000113	0.036315	0.003114	-2.506685491
**TGFB1**	91	3723	0.024443	0.010291	0.711583	0.014462	-1.839783534
**TNFRSF9**	2	72	0.027778	0.000226	0.013761	0.016435	-1.78423439
**S100A8**	452	13173	0.034313	0.051114	2.517775	0.020301	-1.692478147
**LTB**	1431	36656	0.039039	0.161823	7.006116	0.023097	-1.636437328
**XCL1**	91	1739	0.052329	0.010291	0.332378	0.030961	-1.509190079
**CD96**	234	3302	0.070866	0.026462	0.631116	0.041928	-1.377493101
**STAT3**	482	6598	0.073052	0.054506	1.261086	0.043222	-1.364297163
**FN1**	2	25	0.08	0.000226	0.004778	0.047332	-1.324841903
**GZMM**	716	7298	0.098109	0.080968	1.394878	0.058047	-1.236222726
**IFITM1**	886	7840	0.11301	0.100192	1.498471	0.066863	-1.17481423
**TNFRSF18**	84	653	0.128637	0.009499	0.124809	0.076109	-1.118565785
**BLK**	29	220	0.131818	0.003279	0.042049	0.077991	-1.107956573
**ZNF219**	13	95	0.136842	0.00147	0.018157	0.080963	-1.091712143
**ITGA1**	46	262	0.175573	0.005202	0.050076	0.103878	-0.983475349
**FOXP1**	1728	7714	0.224008	0.195409	1.474388	0.132535	-0.877667786
**NSG1**	376	1656	0.227053	0.04252	0.316514	0.134337	-0.871804377
**ADAM15**	95	416	0.228365	0.010743	0.079511	0.135113	-0.869301615
**FTH1**	14533	57656	0.252064	1.643447	11.01988	0.149135	-0.826421125
**ICOSLG**	57	200	0.285	0.006446	0.038226	0.168622	-0.77308703
**EOMES**	144	497	0.289738	0.016284	0.094992	0.171425	-0.765925786
**GATA3**	539	1842	0.292617	0.060952	0.352064	0.173128	-0.76163275
**KLF6**	8885	30044	0.295733	1.00475	5.742355	0.174972	-0.757032211
**KLRD1**	2669	9005	0.296391	0.301821	1.721139	0.175361	-0.756067033
**CD2**	4047	11140	0.363285	0.45765	2.129205	0.214939	-0.667683876
**CCR10**	37	98	0.377551	0.004184	0.018731	0.22338	-0.650956241
**CCR6**	91	215	0.423256	0.010291	0.041093	0.250421	-0.601328957
**ELANE**	3	7	0.428571	0.000339	0.001338	0.253566	-0.595908675
**PDCD1**	39	87	0.448276	0.00441	0.016628	0.265224	-0.576386535
**AZU1**	9	20	0.45	0.001018	0.003823	0.266244	-0.574719376
**TNFRSF25**	701	1460	0.480137	0.079272	0.279052	0.284075	-0.546566728
**KLRG1**	1872	3746	0.499733	0.211693	0.715979	0.295669	-0.529193818
**SPP1**	2	4	0.5	0.000226	0.000765	0.295827	-0.528961885
**CD3G**	6045	12089	0.500041	0.683592	2.310589	0.295852	-0.528925962
**NPDC1**	432	837	0.516129	0.048852	0.159977	0.30537	-0.515173601
**ENTPD1**	501	945	0.530159	0.056655	0.180619	0.313671	-0.503525972
**TCF7**	3723	7006	0.531402	0.421011	1.339067	0.314406	-0.502508986
**LAG3**	964	1799	0.535853	0.109013	0.343846	0.31704	-0.498886019
**TNFRSF4**	217	383	0.56658	0.024539	0.073203	0.335219	-0.47467093
**THBS1**	106	186	0.569892	0.011987	0.03555	0.337179	-0.472138969
**NCAM1**	60	105	0.571429	0.006785	0.020069	0.338088	-0.470969938
**JUN**	28082	45537	0.616685	3.175619	8.703555	0.364865	-0.43786827
**RORA**	4447	7132	0.623528	0.502884	1.36315	0.368913	-0.433076095
**FCGR3B**	9	14	0.642857	0.001018	0.002676	0.380349	-0.419817416
**FOS**	30993	47576	0.651442	3.504806	9.093272	0.385428	-0.414056199
**STAT4**	2825	4325	0.653179	0.319462	0.826644	0.386456	-0.412899549
**KLRC4**	217	316	0.686709	0.024539	0.060398	0.406294	-0.391159238
**ICOSLG**	141	200	0.705	0.015945	0.038226	0.417116	-0.379742773
**TIGIT**	659	917	0.718648	0.074522	0.175268	0.425191	-0.371415811
**CCL5**	64027	87628	0.730668	7.240416	16.74847	0.432303	-0.364211636
**CD7**	9127	12162	0.750452	1.032116	2.324541	0.444008	-0.352608838
**CD70**	261	345	0.756522	0.029515	0.06594	0.447599	-0.349110477
**IL1R2**	68	88	0.772727	0.00769	0.01682	0.457188	-0.339905649
**CNTNAP3**	4	5	0.8	0.000452	0.000956	0.473324	-0.324841903
**BATF**	480	575	0.834783	0.05428	0.109901	0.493903	-0.306358497
**KLRC1**	3488	4120	0.846602	0.394436	0.787462	0.500896	-0.300252629
**LAIR2**	348	403	0.863524	0.039353	0.077026	0.510908	-0.291657692
**GZMK**	11582	13098	0.884257	1.309737	2.50344	0.523175	-0.281353315
**TNFSF14**	199	224	0.888393	0.022504	0.042813	0.525622	-0.279326832
**PRDM1**	1608	1779	0.903879	0.181839	0.340023	0.534784	-0.271821793
**CD8B**	8578	9355	0.916943	0.970033	1.788035	0.542513	-0.26558964
**MKI67**	35	38	0.921053	0.003958	0.007263	0.544945	-0.263647442
**KLRC2**	1064	1078	0.987013	0.120321	0.20604	0.583971	-0.233609023
**GZMH**	16595	16177	1.025839	1.876626	3.091934	0.606942	-0.216852618
**GNLY**	102389	99026	1.033961	11.57854	18.92699	0.611747	-0.213427825
**BIRC5**	72	69	1.043478	0.008142	0.013188	0.617379	-0.209448484
**KIAA0101**	206	196	1.05102	0.023295	0.037462	0.621841	-0.206320741
**PTPRC**	27417	25635	1.069514	3.100418	4.899656	0.632783	-0.198745279
**CD44**	23465	21345	1.099321	2.653511	4.079702	0.650418	-0.18680749
**KLF3**	3971	3574	1.11108	0.449056	0.683104	0.657375	-0.182186551
**CD247**	6479	5403	1.199149	0.73267	1.032683	0.709482	-0.149058877
**PXDN**	10	8	1.25	0.001131	0.001529	0.739568	-0.131021877
**STAT1**	2917	2294	1.271578	0.329865	0.438456	0.752335	-0.123588874
**ATF6B**	5152	4016	1.282869	0.582608	0.767584	0.759015	-0.119749739
**TSPAN32**	3439	2651	1.297246	0.388895	0.50669	0.767522	-0.114909442
**THBD**	281	216	1.300926	0.031777	0.041284	0.769699	-0.113679321
**TNFRSF13C**	269	201	1.338308	0.03042	0.038417	0.791816	-0.101375667
**GZMA**	27871	20568	1.355066	3.151758	3.931193	0.801731	-0.095971402
**FCGR3B**	19	14	1.357143	0.002149	0.002676	0.80296	-0.095306324
**CD3D**	44895	32787	1.369293	5.076897	6.266628	0.810148	-0.091435594
**ZNF683**	2669	1922	1.388658	0.301821	0.367355	0.821605	-0.085336699
**TTC16**	829	585	1.417094	0.093746	0.111812	0.83843	-0.076533225
**ADAM17**	1951	1326	1.471342	0.220626	0.25344	0.870526	-0.060218144
**CXCR3**	1848	1232	1.5	0.208979	0.235474	0.887482	-0.051840631
**TCF4**	333	222	1.5	0.037657	0.042431	0.887482	-0.051840631
**BACH2**	1137	740	1.536486	0.128576	0.141437	0.909069	-0.041403145
**CD40LG**	457	294	1.554422	0.051679	0.056193	0.919681	-0.03636302
**IL12RB1**	1508	961	1.569199	0.17053	0.183677	0.928423	-0.032253936
**FAM65B**	14072	8786	1.601639	1.591315	1.679281	0.947617	-0.023367262
**CTSD**	6783	4220	1.607346	0.767047	0.806575	0.950993	-0.021822524
**JUNB**	97948	60365	1.622596	11.07633	11.53765	0.960016	-0.017721522
**ZAP70**	5975	3630	1.646006	0.675676	0.693807	0.973866	-0.011500605
**CD2**	18574	11140	1.667325	2.100418	2.129205	0.98648	-0.005911639
**STAT6**	2134	1248	1.709936	0.241321	0.238532	1.011691	0.00504794
**PSEN1**	1281	748	1.712567	0.14486	0.142966	1.013248	0.005715642
**PRDM1**	3114	1779	1.750422	0.352143	0.340023	1.035645	0.01521077
**CTSW**	30029	16711	1.79696	3.395793	3.193998	1.063179	0.026606541
**HLA-DQA1**	3100	1692	1.832151	0.35056	0.323394	1.084	0.035029445
**SBK1**	926	504	1.837302	0.104716	0.09633	1.087048	0.036248561
**NCR3**	3992	2171	1.838784	0.451431	0.414946	1.087925	0.036598819
**KCNK5**	24	13	1.846154	0.002714	0.002485	1.092285	0.038336
**CRTAM**	943	503	1.874751	0.106638	0.096139	1.109205	0.045011818
**CD9**	325	173	1.878613	0.036752	0.033066	1.11149	0.045905368
**IL7R**	32561	17238	1.888908	3.682121	3.294725	1.117581	0.048278968
**CD8A**	17171	9056	1.896091	1.941762	1.730887	1.121831	0.049927285
**CBLB**	4766	2472	1.927994	0.538957	0.472477	1.140706	0.057173682
**DOCK8**	6857	3522	1.946905	0.775416	0.673165	1.151895	0.061412908
**BCL11B**	8157	4188	1.947708	0.922425	0.800459	1.15237	0.0615919
**AIM2**	143	71	2.014085	0.016171	0.01357	1.191642	0.076145799
**NKG7**	108579	53730	2.020826	12.27853	10.2695	1.195631	0.077597107

	Raw Counts			Normalized to cell#			
**LYZ**	107433	21087	5.09475	12.14893	4.03039	3.014332	0.479191
**MGST1**	1018	199	5.115578	0.115119	0.038035	3.026654	0.480963
**CCL4**	22872	4458	5.130552	2.586453	0.852064	3.035514	0.482232
**CXCL5**	36	7	5.142857	0.004071	0.001338	3.042794	0.483273
**STK38**	7312	1420	5.149296	0.826869	0.271407	3.046604	0.483816
**AHR**	3205	621	5.161031	0.362434	0.118693	3.053547	0.484805
**CD274**	262	50	5.24	0.029628	0.009557	3.100269	0.491399
**CD80**	21	4	5.25	0.002375	0.000765	3.106186	0.492227
**BIN2**	33630	6386	5.266207	3.803008	1.220566	3.115775	0.493566
**FCGR3A**	25844	4868	5.308956	2.922538	0.930428	3.141068	0.497077
**RUNX3**	31623	5918	5.343528	3.576049	1.131116	3.161522	0.499896
**CD40**	1010	189	5.343915	0.114215	0.036124	3.161751	0.499928
**CD69**	82006	15340	5.345893	9.27355	2.931957	3.162921	0.500088
**FBLN2**	211	39	5.410256	0.023861	0.007454	3.201002	0.505286
**CCR2**	542	100	5.42	0.061291	0.019113	3.206767	0.506067
**RPN2**	14494	2653	5.463249	1.639037	0.507072	3.232355	0.509519
**CHI3L2**	1697	310	5.474194	0.191903	0.059251	3.238831	0.510388
**NT5E**	952	173	5.50289	0.107656	0.033066	3.255809	0.512659
**IL18R1**	1099	199	5.522613	0.124279	0.038035	3.267478	0.514213
**VCAN**	11662	2105	5.540143	1.318783	0.402332	3.27785	0.515589
**PTGDS**	3870	693	5.584416	0.437634	0.132454	3.304044	0.519046
**LAMP1**	20186	3604	5.600999	2.282709	0.688838	3.313856	0.520334
**FYN**	27226	4820	5.648548	3.078819	0.921254	3.341988	0.524005
**IL12A**	231	40	5.775	0.026122	0.007645	3.416804	0.53362
**ICOS**	2463	426	5.78169	0.278525	0.081422	3.420763	0.534123
**TNFSF13B**	3073	524	5.864504	0.347507	0.100153	3.46976	0.540299
**ADA**	5259	895	5.875978	0.594708	0.171063	3.476548	0.541148
**CD244**	2678	455	5.885714	0.302838	0.086965	3.482309	0.541867
**GIMAP2**	11943	2025	5.897778	1.35056	0.387041	3.489446	0.542757
**CD24**	427	72	5.930556	0.048287	0.013761	3.508839	0.545163
**CMTM2**	107	18	5.944444	0.0121	0.00344	3.517057	0.546179
**CD28**	4235	708	5.981638	0.47891	0.135321	3.539063	0.548888
**IL3RA**	565	94	6.010638	0.063892	0.017966	3.556221	0.550989
**CD48**	86413	14277	6.052602	9.77191	2.728784	3.581049	0.55401
**IFNGR1**	6866	1122	6.11943	0.776433	0.21445	3.620588	0.558779
**C1QB**	374	61	6.131148	0.042293	0.011659	3.627521	0.55961
**FCER1A**	3685	599	6.15192	0.416714	0.114488	3.639811	0.561079
**XCL2**	6559	1058	6.199433	0.741717	0.202217	3.667922	0.56442
**KLRF1**	9438	1515	6.229703	1.067285	0.289564	3.685831	0.566535
**IRF7**	4474	710	6.301408	0.505937	0.135703	3.728256	0.571506
**FUT4**	354	56	6.321429	0.040032	0.010703	3.740101	0.572883
**CCL22**	19	3	6.333333	0.002149	0.000573	3.747145	0.5737
**CCR3**	19	3	6.333333	0.002149	0.000573	3.747145	0.5737
**ITGAM**	6309	988	6.385628	0.713446	0.188838	3.778085	0.577272
**HLA-DPA1**	49085	7672	6.397941	5.550718	1.466361	3.78537	0.578108
**TLR2**	1785	278	6.420863	0.201855	0.053135	3.798932	0.579662
**S100A9**	99413	15416	6.44869	11.242	2.946483	3.815396	0.58154
**ZBTB16**	1880	291	6.460481	0.212598	0.055619	3.822372	0.582333
**MXRA8**	182	28	6.5	0.020581	0.005352	3.845754	0.584981
**SELPLG**	21267	3250	6.543692	2.404953	0.621177	3.871604	0.587891
**ANXA5**	13505	2023	6.675729	1.527197	0.386659	3.949725	0.596567
**IL2**	87	13	6.692308	0.009838	0.002485	3.959533	0.597644
**PCNA**	3715	552	6.730072	0.420106	0.105505	3.981877	0.600088
**VPREB3**	1515	225	6.733333	0.171322	0.043005	3.983806	0.600298
**CXCR4**	212805	31249	6.809978	24.0648	5.972668	4.029154	0.605214
**DPP4**	3983	584	6.820205	0.450413	0.111621	4.035205	0.605866
**LTA**	1769	255	6.937255	0.200045	0.048739	4.104457	0.613256
**AURKB**	174	25	6.96	0.019677	0.004778	4.117915	0.614677
**FOSB**	136646	19532	6.996007	15.45245	3.73318	4.139218	0.616918
**CLEC10A**	1699	242	7.020661	0.192129	0.046254	4.153805	0.618446
**LILRB4**	1500	213	7.042254	0.169626	0.040711	4.16658	0.61978
**TYMS**	361	51	7.078431	0.040823	0.009748	4.187985	0.622005
**TNF**	8171	1138	7.180141	0.924008	0.217508	4.248162	0.628201
**TNFRSF8**	216	30	7.2	0.024426	0.005734	4.259912	0.629401
**SELL**	46325	6315	7.335709	5.238607	1.206995	4.340204	0.63751
**LIPA**	8299	1130	7.344248	0.938482	0.215979	4.345257	0.638015
**ITGAX**	5106	695	7.346763	0.577406	0.132836	4.346745	0.638164
**CX3CR1**	3042	413	7.365617	0.344001	0.078937	4.3579	0.639277
**KCNE3**	914	124	7.370968	0.103359	0.0237	4.361066	0.639593
**TOX**	1894	255	7.427451	0.214181	0.048739	4.394484	0.642908
**CCR6**	1601	215	7.446512	0.181047	0.041093	4.405761	0.644021
**FCER1G**	31374	4177	7.511132	3.547891	0.798356	4.443995	0.647774
**VEGFA**	1533	204	7.514706	0.173357	0.038991	4.446109	0.64798
**CSF1**	279	37	7.540541	0.03155	0.007072	4.461394	0.649471
**TGFB3**	276	36	7.666667	0.031211	0.006881	4.536017	0.656675
**MZB1**	1741	227	7.669604	0.196879	0.043387	4.537755	0.656841
**ITGB2**	75666	9726	7.779766	8.556598	1.858945	4.602933	0.663035
**TRAT1**	7378	946	7.799154	0.834332	0.18081	4.614404	0.664116
**IER3**	13020	1638	7.948718	1.472351	0.313073	4.702894	0.672365
**B3GAT1**	916	115	7.965217	0.103585	0.02198	4.712656	0.673266
**HLA-DMA**	16420	2056	7.986381	1.856836	0.392966	4.725178	0.674418
**MYC**	6857	856	8.010514	0.775416	0.163609	4.739456	0.675728
**IL10**	163	20	8.15	0.018433	0.003823	4.821983	0.683226
**CLEC4D**	432	53	8.150943	0.048852	0.01013	4.822542	0.683276
**BCL2A1**	11911	1447	8.231513	1.346941	0.276567	4.870211	0.687548
**TBX21**	6745	819	8.235653	0.76275	0.156537	4.872661	0.687766
**TNFRSF1B**	18711	2262	8.271883	2.115911	0.432339	4.894096	0.689673
**CCL4L2**	4038	481	8.39501	0.456632	0.091934	4.966945	0.696089
**EGR3**	361	43	8.395349	0.040823	0.008219	4.967145	0.696107
**RNASE6**	3444	406	8.482759	0.389461	0.077599	5.018862	0.700605
**PIK3IP1**	48078	5661	8.492846	5.436843	1.081995	5.02483	0.701121
**TMEM123**	56255	6621	8.496451	6.361529	1.265482	5.026963	0.701306
**RNASE2**	1271	149	8.530201	0.14373	0.028479	5.046931	0.703027
**CCR7**	10834	1265	8.564427	1.22515	0.241781	5.067181	0.704766
**IRF4**	1234	140	8.814286	0.139545	0.026758	5.215011	0.717255
**LAMP3**	287	32	8.96875	0.032455	0.006116	5.306401	0.7248
**JCHAIN**	6644	739	8.990528	0.751329	0.141246	5.319285	0.725853
**CD207**	54	6	9	0.006107	0.001147	5.32489	0.726311
**HLA-DRA**	113741	12396	9.175621	12.86226	2.369266	5.428797	0.734704
**CXCR1**	83	9	9.222222	0.009386	0.00172	5.456369	0.736904
**LAT2**	5318	568	9.362676	0.60138	0.108563	5.539469	0.743468
**IFITM3**	26465	2796	9.465308	2.992763	0.534404	5.600191	0.748203
**ADGRE1**	853	90	9.477778	0.09646	0.017202	5.607569	0.748775
**FLT3**	418	44	9.5	0.047269	0.00841	5.620717	0.749792
**ITGA5**	5034	527	9.552182	0.569264	0.100726	5.651591	0.752171
**BTLA**	643	67	9.597015	0.072713	0.012806	5.678116	0.754204
**EGR1**	7898	822	9.608273	0.893136	0.15711	5.684777	0.754713
**STAT5A**	6542	668	9.793413	0.739794	0.127676	5.794316	0.763002
**PTPN6**	24415	2483	9.832863	2.760941	0.47458	5.817657	0.764748
**FCER2**	1191	120	9.925	0.134683	0.022936	5.87217	0.768799
**LIF**	50	5	10	0.005654	0.000956	5.916544	0.772068

VPS28	9035	4417	2.045506	1.021712	0.844228	1.210233	0.082868868
VMO1	198	96	2.0625	0.022391	0.018349	1.220287	0.086462068
IFITM2	33211	16071	2.066517	3.755626	3.071674	1.222664	0.087307162
KDELR1	2400	1156	2.076125	0.271401	0.220948	1.228348	0.089321518
TXK	5293	2484	2.130837	0.598553	0.474771	1.260719	0.100618413
PDCD1LG2	30	14	2.142857	0.003393	0.002676	1.267831	0.103061329
FOXO3	1971	915	2.154098	0.222888	0.174885	1.274482	0.105333641
CLU	446	204	2.186275	0.050435	0.038991	1.293519	0.111772802
ALAS2	20	9	2.222222	0.002262	0.00172	1.314788	0.118855597
ZBED2	9	4	2.25	0.001018	0.000765	1.331222	0.124250628
IKZF1	9403	4146	2.267969	1.063327	0.792431	1.341854	0.127705249
RAP1GAP2	1608	708	2.271186	0.181839	0.135321	1.343757	0.128320897
CD27	14646	6431	2.277406	1.656225	1.229167	1.347438	0.129508631
TMEM97	258	112	2.303571	0.029176	0.021407	1.362918	0.134469794
TIMP1	12975	5616	2.310363	1.467262	1.073394	1.366937	0.135748378
AIF1	12935	5575	2.320179	1.462739	1.065558	1.372744	0.137589672
IL15	419	179	2.340782	0.047382	0.034213	1.384934	0.141429102
HAVCR2	1493	634	2.35489	0.168834	0.121177	1.393281	0.14403866
GNAI2	8341	3509	2.37703	0.943232	0.67068	1.406381	0.148102863
DUSP4	859	357	2.406162	0.097139	0.068234	1.423617	0.153393058
TLR7	85	35	2.428571	0.009612	0.00669	1.436875	0.157418992
BIRC3	4154	1674	2.481481	0.46975	0.319954	1.468179	0.166779149
CMKLR1	354	142	2.492958	0.040032	0.027141	1.474969	0.168783028
HOPX	15668	6259	2.503275	1.771797	1.196292	1.481074	0.170576722
SLC25A37	3300	1307	2.524866	0.373177	0.249809	1.493848	0.174306463
TREM1	1645	646	2.54644	0.186023	0.123471	1.506612	0.178001495
EBF1	161	63	2.555556	0.018206	0.012041	1.512006	0.179553437
SOCS3	5137	2006	2.560818	0.580911	0.38341	1.515119	0.180446747
APOBEC3G	8256	3215	2.567963	0.93362	0.614488	1.519346	0.181656817
IL32	127779	48976	2.609013	14.44973	9.360856	1.543634	0.188544283
CCR1	444	170	2.611765	0.050209	0.032492	1.545262	0.189002159
CD160	2000	764	2.617801	0.226168	0.146024	1.548834	0.190004747
LGALS1	30948	11401	2.714499	3.499717	2.17909	1.606045	0.205757753
CD52	185215	67835	2.730375	20.94482	12.96541	1.615439	0.208290437
PRF1	17485	6395	2.734167	1.97727	1.222286	1.617682	0.208893198
MMP9	22	8	2.75	0.002488	0.001529	1.62705	0.211400804
PDIA4	4171	1506	2.769588	0.471673	0.287844	1.638639	0.214483328
GZMB	18568	6691	2.775071	2.09974	1.278861	1.641883	0.215342208
FAM129C	345	124	2.782258	0.039014	0.0237	1.646135	0.21646552
IL23A	1429	508	2.812992	0.161597	0.097095	1.664319	0.221236627
CST7	29835	10522	2.835488	3.373855	2.011086	1.677629	0.224695855
IL6R	1817	629	2.888712	0.205473	0.120222	1.709119	0.232772392
TOP2A	203	70	2.9	0.022956	0.013379	1.715798	0.234466108
BCL2	5023	1696	2.961675	0.56802	0.324159	1.752288	0.243605441
MCM4	409	138	2.963768	0.046251	0.026376	1.753526	0.243912332
LAT	19297	6405	3.012802	2.182178	1.224197	1.782538	0.251038773
BTG1	224167	72851	3.077061	25.34966	13.92412	1.820557	0.260204272
CXCR6	133	43	3.093023	0.01504	0.008219	1.830001	0.262451296
NRP1	19	6	3.166667	0.002149	0.001147	1.873572	0.272670461
CD38	606	187	3.240642	0.068529	0.035742	1.91734	0.282699128
DUSP2	63937	19729	3.240762	7.230239	3.770833	1.917411	0.282715292
IKZF2	869	266	3.266917	0.09827	0.050841	1.932886	0.28620625
FYB	33389	10174	3.281797	3.775755	1.944572	1.94169	0.288179789
PDIA6	9147	2772	3.299784	1.034377	0.529817	1.952332	0.290553563
CTSS	30120	9084	3.31572	3.406084	1.736239	1.96176	0.292645952
ADAM28	959	286	3.353147	0.108447	0.054664	1.983904	0.297520684
NINJ2	1106	325	3.403077	0.125071	0.062118	2.013445	0.303939876
AXL	82	24	3.416667	0.009273	0.004587	2.021486	0.305670721
MX2	3497	1021	3.425073	0.395454	0.195145	2.02646	0.306738001
YBX3	7574	2211	3.425599	0.856497	0.422592	2.026771	0.306804669
GAPDH	84429	24641	3.426363	9.547552	4.709671	2.027223	0.306901427
NAMPT	14697	4277	3.436287	1.661993	0.817469	2.033094	0.308157554
HLA-DPB1	36490	10609	3.439532	4.126428	2.027714	2.035015	0.308567521
IFNG	3881	1120	3.465179	0.438878	0.214067	2.050188	0.31179373
XBP1	18546	5304	3.496606	2.097252	1.013761	2.068783	0.31571485
CD36	1941	553	3.509946	0.219496	0.105696	2.076675	0.317368514
CD63	23331	6618	3.525385	2.638358	1.264908	2.08581	0.319274701
CD79B	5205	1466	3.550477	0.588601	0.280199	2.100656	0.322354874
ID3	1059	291	3.639175	0.119756	0.055619	2.153134	0.333071081
MITF	73	20	3.65	0.008255	0.003823	2.159539	0.334360975
CCND2	5268	1425	3.696842	0.595725	0.272362	2.187253	0.339899012
C1QA	584	155	3.767742	0.066041	0.029625	2.229201	0.348149259
KLRB1	39182	10383	3.773668	4.430849	1.984518	2.232708	0.348831857
CD34	38	10	3.8	0.004297	0.001911	2.248287	0.351851707
SOX13	478	125	3.824	0.054054	0.023891	2.262486	0.354585994
MNDA	4667	1204	3.876246	0.527762	0.230122	2.293398	0.360479424
IGBP1	8275	2122	3.899623	0.935768	0.405581	2.307229	0.363090733
CNOT2	6596	1667	3.956809	0.745901	0.318616	2.341063	0.369413157
HLA-A	452441	113704	3.979112	51.16375	21.73242	2.354259	0.371854321
BAX	12273	3068	4.000326	1.387877	0.586391	2.366811	0.374163489
KLF2	37252	9229	4.036407	4.212598	1.763953	2.388158	0.378063059
MX1	3174	784	4.048469	0.358928	0.149847	2.395295	0.37935897
LCK	35362	8707	4.06133	3.998869	1.664182	2.402904	0.380736386
ARL4C	48347	11865	4.074758	5.467262	2.267775	2.410848	0.382169898
DUSP6	4376	1064	4.112782	0.494855	0.203364	2.433346	0.386203796
LAP3	4002	967	4.138573	0.452561	0.184824	2.448605	0.388918721
ITGAE	4388	1060	4.139623	0.496212	0.202599	2.449226	0.389028864
IL2RA	820	197	4.162437	0.092729	0.037653	2.462724	0.391415737
HIF1A	10460	2506	4.173982	1.182856	0.478976	2.469555	0.392618728
UBE2C	88	21	4.190476	0.009951	0.004014	2.479314	0.394331488
LGALS3	8537	2008	4.251494	0.965396	0.383792	2.515415	0.400609683
S100A10	64095	14993	4.274995	7.248106	2.865635	2.52932	0.403003721
CCR5	352	79	4.455696	0.039805	0.015099	2.636232	0.420983682
CD300A	6625	1462	4.531464	0.74918	0.279434	2.681061	0.42830662
ITGA4	12828	2805	4.573262	1.450639	0.536124	2.705791	0.432294196
CD3E	62665	13622	4.600279	7.086396	2.603593	2.721775	0.434852278
FAS	2328	506	4.600791	0.263259	0.096713	2.722078	0.434900569
IL15RA	817	176	4.642045	0.092389	0.033639	2.746487	0.438777499
FASLG	288	62	4.645161	0.032568	0.01185	2.74833	0.439068909
HLA-DQB1	21662	4637	4.671555	2.449621	0.886277	2.763946	0.441529566
CDH1	38	8	4.75	0.004297	0.001529	2.810358	0.44876172
TRAC	103183	21659	4.763978	11.66833	4.139717	2.818629	0.45003786
CCR4	106	22	4.818182	0.011987	0.004205	2.850699	0.454951295
SNCA	323	67	4.820896	0.036526	0.012806	2.852304	0.45519583
C10orf54	25072	5148	4.870241	2.835237	0.983945	2.8815	0.459618551
CD37	59955	12251	4.893886	6.779939	2.341552	2.895489	0.461721978
FBN2	55	11	5	0.00622	0.002102	2.958272	0.471038115
MME	40	8	5	0.004523	0.001529	2.958272	0.471038115
HMMR	35	7	5	0.003958	0.001338	2.958272	0.471038115
BCL6	2847	569	5.003515	0.32195	0.108754	2.960352	0.471343311
TNFSF10	3903	779	5.01027	0.441366	0.148891	2.964348	0.471929204
FOXO1	2900	575	5.043478	0.327943	0.109901	2.983996	0.474798264
CD72	772	153	5.045752	0.087301	0.029243	2.985341	0.47499398

CD86	2138	213	10.03756	0.241773	0.040711	5.938766	0.773696
RHOA	71167	7090	10.03766	8.047834	1.355122	5.938825	0.773701
F5	784	78	10.05128	0.088658	0.014908	5.946885	0.77429
CD5	11114	1090	10.19633	1.256813	0.208333	6.032704	0.780512
CLC	31	3	10.33333	0.003506	0.000573	6.113762	0.786309
MS4A1	8859	854	10.37354	1.001809	0.163226	6.137549	0.787995
LY86	5891	562	10.48221	0.666177	0.107416	6.201844	0.792521
CD209	42	4	10.5	0.00475	0.000765	6.212371	0.793257
TIMP2	2725	258	10.56202	0.308153	0.049312	6.249063	0.795815
CXCL10	74	7	10.57143	0.008368	0.001338	6.254632	0.796202
IL4R	12609	1189	10.60471	1.425874	0.227255	6.274323	0.797567
CD74	261924	24617	10.63996	29.61936	4.705084	6.295182	0.799008
TNFSF8	4195	392	10.70153	0.474387	0.074924	6.331608	0.801514
IL18	516	48	10.75	0.058351	0.009174	6.360285	0.803477
LYN	10189	932	10.9324	1.152211	0.178135	6.468205	0.810784
CD6	32129	2929	10.96927	3.633269	0.559824	6.490019	0.812246
QPCT	1067	96	11.11458	0.12066	0.018349	6.575992	0.817961
VNN2	3607	323	11.16718	0.407893	0.061735	6.607113	0.820012
TSPAN18	2308	203	11.36946	0.260997	0.0388	6.72679	0.827808
VSIG4	205	18	11.38889	0.023182	0.00344	6.738286	0.828549
TRBC2	149823	13083	11.45173	16.94255	2.500573	6.775467	0.830939
CD33	2294	200	11.47	0.259414	0.038226	6.786276	0.831632
IGKC	55156	4770	11.5631	6.23725	0.911697	6.841361	0.835142
CD79A	17291	1494	11.57363	1.955332	0.28555	6.847588	0.835538
ICAM1	2634	226	11.65487	0.297863	0.043196	6.895654	0.838575
IL12RB2	317	27	11.74074	0.035848	0.005161	6.946461	0.841764
F13A1	1549	131	11.82443	0.175167	0.025038	6.995975	0.844848
CCL3L3	15572	1283	12.13718	1.760941	0.245222	7.181015	0.856186
SLC7A7	3434	280	12.26429	0.38833	0.053517	7.256219	0.86071
FOS	585716	47576	12.31117	66.23499	9.093272	7.283955	0.862367
LGALS9	7372	593	12.4317	0.833654	0.113341	7.355272	0.866599
CXCR2	411	33	12.45455	0.046477	0.006307	7.368787	0.867396
GAB2	1244	99	12.56566	0.140676	0.018922	7.434526	0.871253
TRDC	11861	926	12.80886	1.341287	0.176988	7.578416	0.879578
CD200	334	26	12.84615	0.03777	0.004969	7.600484	0.880841
CD1A	65	5	13	0.00735	0.000956	7.691507	0.886011
TLR8	496	38	13.05263	0.05609	0.007263	7.722647	0.887766
PIK3AP1	5677	434	13.08065	0.641977	0.082951	7.739221	0.888697
CD14	16036	1222	13.12275	1.813412	0.233563	7.764133	0.890093
PTTG2	132	10	13.2	0.014927	0.001911	7.809838	0.892642
TTYH2	2231	168	13.27976	0.25229	0.03211	7.85703	0.895258
FCN1	50958	3809	13.37831	5.762524	0.72802	7.915339	0.89847
CAPG	9453	705	13.40851	1.068981	0.134748	7.933205	0.899449
CLEC4E	2507	186	13.47849	0.283501	0.03555	7.974611	0.90171
CR2	445	33	13.48485	0.050322	0.006307	7.97837	0.901914
LEF1	28736	2128	13.50376	3.249576	0.406728	7.989559	0.902523
AQP9	907	67	13.53731	0.102567	0.012806	8.009411	0.903601
CCR9	122	9	13.55556	0.013796	0.00172	8.020204	0.904185
IL2RB	27617	1986	13.90584	3.123035	0.379587	8.227452	0.915265
BLNK	1032	74	13.94595	0.116702	0.014144	8.251181	0.916516
PTGDR2	224	16	14	0.025331	0.003058	8.283162	0.918196
PASK	10390	737	14.09769	1.174941	0.140864	8.340963	0.921216
SERPINA1	22155	1552	14.27513	2.505371	0.296636	8.445943	0.926648
DUSP1	361619	24942	14.4984	40.89325	4.767202	8.57804	0.933388
CD1C	2921	201	14.53234	0.330318	0.038417	8.598122	0.934404
IRF8	9205	632	14.56487	1.040936	0.120795	8.617372	0.935375
S100A12	18492	1258	14.69952	2.091146	0.240443	8.697038	0.939371
CCL3	28499	1835	15.53079	3.222775	0.350726	9.188861	0.963262
CD163	1679	103	16.30097	0.189868	0.019687	9.644541	0.984282
CXCL16	6149	374	16.44118	0.695352	0.071483	9.727495	0.988001
IL18RAP	3289	199	16.52764	0.371933	0.038035	9.77865	0.990279
TRIB2	7065	418	16.90191	0.798937	0.079893	10.00009	1.000004
TCL1A	1785	105	17	0.201855	0.020069	10.05813	1.002517
MCM2	1011	59	17.13559	0.114328	0.011277	10.13835	1.005967
CTSK	1377	79	17.43038	0.155716	0.015099	10.31276	1.013375
RORC	598	33	18.12121	0.067624	0.006307	10.7215	1.030255
TGFBI	9661	511	18.90607	1.092503	0.097668	11.18586	1.048669
LRRC32	95	5	19	0.010743	0.000956	11.24143	1.050822
RGS1	52303	2656	19.69239	5.914622	0.507645	11.65109	1.066367
S1PR1	18459	933	19.78457	2.087414	0.178326	11.70563	1.068395
KLF2	183528	9229	19.88601	20.75404	1.763953	11.76565	1.070616
ZFP36	444245	22249	19.96696	50.23691	4.252485	11.81354	1.07238
CD68	16656	797	20.89837	1.883524	0.152332	12.36461	1.092181
CD83	22925	1090	21.03211	2.592446	0.208333	12.44374	1.094951
CD22	4808	219	21.95434	0.543707	0.041858	12.98938	1.113588
PAX5	1647	75	21.96	0.186249	0.014335	12.99273	1.1137
NLRP3	8643	371	23.2965	0.977383	0.07091	13.78347	1.139359
FOSL1	573	23	24.91304	0.064797	0.004396	14.73991	1.168495
CD4	18434	717	25.7099	2.084587	0.137041	15.21138	1.182169
TNFRSF17	182	7	26	0.020581	0.001338	15.38301	1.187041
CXCL2	24769	940	26.35	2.800973	0.179664	15.59009	1.192849
IL1B	64692	2356	27.4584	7.315617	0.450306	16.24589	1.210743
CTLA4	2023	70	28.9	0.228769	0.013379	17.09881	1.232966
KLRC3	1537	53	29	0.17381	0.01013	17.15798	1.234466
CXCL8	139272	4538	30.69017	15.74941	0.867355	18.15798	1.259067
IL1RN	3121	101	30.90099	0.352935	0.019304	18.28271	1.262041
CXCR5	1780	56	31.78571	0.201289	0.010703	18.80616	1.2743
CXCL3	10693	325	32.90154	1.209205	0.062118	19.46634	1.289284
GIMAP5	4978	148	33.63514	0.562931	0.028287	19.90038	1.298861
KLRK1	15327	444	34.52027	1.733235	0.084862	20.42407	1.310142
BPI	312	9	34.66667	0.035282	0.00172	20.51069	1.31198
ADGRG3	178	5	35.6	0.020129	0.000956	21.0629	1.323518
IGLC3	64901	1387	46.79236	7.339251	0.265099	27.68491	1.442243
KIR2DL1	764	14	54.57143	0.086396	0.002676	32.28743	1.509033
POU2AF1	2240	41	54.63415	0.253308	0.007836	32.32453	1.509532
IL23R	3123	48	65.0625	0.353161	0.009174	38.49452	1.585399
FOXP3	1363	19	71.73684	0.154133	0.003631	42.44342	1.62781
CXCL1	1868	22	84.90909	0.211241	0.004205	50.23684	1.701022
IL6	2503	21	119.1905	0.283049	0.004014	70.51957	1.84831
CCL20	2024	13	155.6923	0.228882	0.002485	92.11604	1.964335
TNFSF13	3565	7	509.2857	0.403144	0.001338	301.3211	2.47903
TIAF1	5517	8	689.625	0.623883	0.001529	408.0197	2.610681
CASP5	9095	4	2273.75	1.028497	0.000765	1345.274	3.128811

Supplementary Table 3, related to main figure 2
List of genes overlapping/only detect in one assay between targeted transcriptomics and WTA in the indicated cluste

CD4 cluster		
**Gene overlap**	**Targeted only**	**WTA only**
JUNB	LEF1	LTB
IL7R	TMEM123	MALAT1
TRAC	FYB	ZFP36L2
CD69	FOS	RPS21
CD3D	PIK3IP1	SARAF
BTG1	CD4	AAK1
CXCR4	KLF2	PABPC1
CD44	CD6	GSTK1
	CD48	ACAP1
	CD27	FXYD5
	TRBC2	RP11-347P5.1
	SELL	TRBC1
	CD3E	ITGB1
	KLF2	SLC2A3
	TRAT1	SRSF7
	CD52	TSC22D3
	LAT	SF1
	FOS	CCNL1
	CD5	PRRC2C
	CCR7	N4BP2L2
	FOSB	
	IL32	
	S1PR1	
	FAM65B	
	BCL11B	
	ITGA4	
	GIMAP2	
	TRIB2	
	RGS1	
	CD2	
	LCK	
	XBP1	
	IKZF1	
	RPN2	
	IL4R	
	VPS28	
	STK38	
	CD3G	
	ARL4C	
	FYN	
	PTPRC	
	KLF6	
	TIMP1	

NK cluster			
**Gene overlap**	**Targeted only**	**WTA only**	
NKG7	TRDC	FGFBP2	FAM49B
PRF1	IL2RB	SPON2	PDIA3
GZMB	TBX21	TYROBP	CALR
GNLY	KLRK1	CLIC3	IL2RG
FCGR3A	HOPX	S1PR5	RP11-160E2.6
KLRD1	KLRC1	HLA-C	EVL
CTSW	RUNX3	CMC1	LCP1
GZMA	ITGAM	PLAC8	H3F3A
KLRF1	CX3CR1	GZMM	HNRNPA2B1
CST7	ITGAX	ACTB	SRP14
CD247	CD160	HLA-A	PSME1
KLRB1	CD300A	EFHD2	TAPBP
GZMH	CD244	HLA-B	DDX6
CD7	CCL3	MYO1F	CD53
CCL4	XCL2	ZEB2	LY6E
CCL5	NCR3	PFN1	PPP2R5C
FCER1G	LAT2	UBB	AES
ITGB2	IL18RAP	HCST	MYL12B
IFITM2	TARP	CLIC1	HNRNPA3
CTSD	APOBEC3G	ACTG1	ID2
CD63	PIK3AP1	ARPC2	SERF2
BIN2	LYN	HLA-E	MYL6
ARL4C	IFNG	JAK1	CCDC12
	TXK	SPN	SH3BGRL3
	IFITM3	FLNA	COX6C
	ZAP70	MYL12A	SSR2
	SELPLG	CFL1	RSRP1
	LAMP1	SUN2	PPIA
	STK38	CALM1	ZFP36
		CCND3	HNRNPK
		CYBA	
		ANXA6	
		PSMB9	
		CD99	
		RAC2	
		UCP2	
		43715	
		LSP1	
		RARRES3	
		LITAF	
		RNF213	
		IRF1	
		CORO1A	

Monocyte cluster				
**Gene overlap**	**Targeted only**	**WTA only**		
LYZ	CLEC4E	SAT1	PTPRE	ATP6V0B
FCN1	TREM1	NEAT1	MT-ND1	PTP4A2
FTH1	SLC7A7	FTL	PHACTR1	CMTM6
NAMPT	NLRP3	CLEC7A	HLA-DRB5	ARRB2
S100A9	TGFBI	S100A8	GPX1	POU2F2
VCAN	TLR2	CSF3R	S100A11	VIM
CTSS	CXCL16	SLC11A1	CEBPD	KLF6
FOS	CXCL2	TYMP	RP11-386I14.4	TALDO1
FOS	RNASE2	PLAUR	AP1S2	RP5-1171I10.5
SERPINA1	KCNE3	RP11-1143G9.	JUND	FMNL1
CXCL8	LGALS3	CST3	ZEB2	ANXA1
CD14	CCL3L3	PSAP	RHOB	FKBP1A
MNDA	CD163	KLF4	HLA-DRB1	SDCBP
IL1B	MGST1	LINC00936	GABARAPL1	AP2S1
AIF1	VNN2	SPI1	PTCH2	WSB1
DUSP1	CD33	SGK1	RNF130	JMJD1C
HIF1A	QPCT	CFD	TKT	ACTR2
S100A12	BCL6	MT-CO1	NPC2	LCP1
CD36	TIMP2	IFI30	CTSB	GLIPR1
CD68	VEGFA	RP11-160E2.6	FCGRT	ANXA2
DUSP6	GAB2	G0S2	LYST	LAMTOR4
FCER1G	LAP3	FGL2	MXD1	C4orf48
BCL2A1	CD86	LST1	GPCPD1	LRRFIP1
HLA-DRA	CCL3	MAFB	APLP2	TCOF1
IER3	F13A1	CYBB	SOD2	TLE4
HLA-DQB1	ITGAX	SRGN	FOSL2	NR4A2
TNFRSF1B	TNFSF10	CEBPB	STXBP2	MYO1F
FOSB	YBX3	MCL1	TMEM107	LAPTM4A
LYN	IFNGR1	RGS2	VMP1	DDX21
CD74	ANXA5	CD300E	H2AFY	STK17B
IFITM3	CD83	S100A6	S100A4	SAT2
TIMP1	SLC25A37	CLEC12A	PYCARD	RTN4
ZFP36	RNASE6	MPEG1	ZFAND5	TUBA1A
LGALS1	LAT2	CH17-373J23.	JAML	UBC
CTSD	CAPG	GLUL	CTNNB1	VASP
HLA-DMA	SOCS3	C5AR1	MEF2C	EVI2B
C10orf54	AHR	TYROBP	WDR74	IRF2BP2
HLA-DPA1	LIPA	TNFAIP2	FGR	IQGAP1
HLA-DPB1	LY86	NFKBIA	ZFP36L1	CD44
	LGALS9	LILRB2	MYADM	NOP10
	IRF8	CSTA	ATP2B1	RNH1
	TNFSF13B	RAB31	MIDN	CAPZA1
	PIK3AP1	MS4A6A	PLEK	DAZAP2
	CD63	GRN	RNU12	HNRNPU
	TNFSF13	COTL1	GNAI2	DDX3X
	LILRB4	LGALS2	RAC1	VAPA
	PTPN6	CFP	C1orf162	EFHD2
	FCGR3A	ASAH1	GSTP1	SAMHD1
		SLC43A2	HLA-DMB	IL10RA
		NFKBIZ	NCF1	ATF4

Supplementary Table 4, related to STAR methods
List of staining dilutions and fluorochrome-conjugates used

FACSymphony **Panel 1 - T cell centric**

		Flourophore	Antigen	Dilution
1	355nm	BUV395	CD183 (CXCR3)	1: 20
2		UV Blue L/D	N.A	1: 500
3		BUV496	CD3	1: 40
4		BUV563	CD25	1: 40
5		BUV661	HLA-DR	1: 80
6		BUV737	ICOS	1: 20
7		BUV805	CD8	1: 80
8	405nm	BV421	CD196 (CCR6)	1: 20
9		BV480	TCRgd	1: 20
10		BV570	CD14	1: 20
11		BV605	PD1	1: 20
12		BV650	CD69	1: 20
13		BV711	CD45RA	1: 320
14		BV750	CD103	1: 160
15		BV785	CD127	1: 10
16	488nm	BB515	Tim3	1: 80
17		BB630	CD16	1: 1280
18		BB660	CD27	1: 160
19		BB700	CD161	1: 20
20		BB790	CD38	1: 80
21	532nm	PE	CD194 (CCR4)	1: 20
22		PE-CF594	CD39	1: 80
23		PE-Cy5	CD137	1: 20
24		PE-Cy5.5	CD19	1: 160
25		PE-Cy7	CD197 (CCR7)	1: 40
26	628nm	APC	EBV-TET	1: 200
27		AF700	CD45RO	1: 20
28		APC-H7	CD4	1: 40

FACSymphony **Panel 2 - APC centric**

		Flourophore	Antigen	Dilution
1	355nm	BUV395	CD40	1: 40
2		UV Blue L/D	N.A	1: 500
3		BUV496	CD3	1: 40
4		BUV563	CD56	1: 160
5		BUV661	EMPTY	
6		BUV737	CD86	1: 20
7		BUV805	CD8	1: 40
8	405nm	BV421	CX3CR1	1: 20
9		BV480	CD28	1: 40
10		BV570	CD14	1: 20
11		BV605	CD141	1: 320
12		BV650	Sirpa	1: 160
13		BV711	OX40	1: 40
14		BV750	CD11b	1: 160
15		BV785	CD123	1: 40
16	488nm	BB515	CD206	1: 20
17		BB630	CD16	1: 1280
18		BB660	CD27	1: 160
19		BB700	CD32	1: 160
20		BB790	CD38	1: 80
21	532nm	PE	Lag3	1: 20
22		PE-CF594	CD163	1: 80
23		PE-Cy5	CD80	1: 10
24		PE-Cy5.5	CD19	1: 160
25		PE-Cy7	CD4	1: 80
26	628nm	AF647	CD1c	1: 160
27		AF700	CD11c	1: 320
28		APC-H7	HLA-DR	1: 40

Transcriptomics - Oligonucleotide-Antibodies

	AbSeq-AbO	Stock mg/ml	µg used per stain
1	CD3	0.125	0.25
2	CD4	0.03	0.06
3	CD8	0.125	0.25
4	CD19	0.03	0.06
5	CD14	0.125	0.25
6	CD16	0.25	0.5
7	CD56	0.0625	0.125
8	CD11b	0.03	0.06
9	CD25	0.125	0.25
10	HLA-DR	0.5	1
11	CD45RA	0.0625	0.125
12	CD127	0.125	0.25
13	CD38	0.25	0.5
14	CD197	0.25	0.5
15	CD279	0.25	0.5
16	CD28	0.125	0.25
17	CD279	0.125	0.25
18	CD69	0.125	0.25
19	CD123	0.25	0.5
20	CD45RO	0.25	0.5
21	CD11c	0.25	0.5
22	CD86	0.0625	0.125
23	CD183	0.5	1
24	CD196	0.25	0.5
25	CD80	0.125	0.25
26	CD278	0.03	0.06
27	CD194	0.25	0.5
28	CD40	0.015	0.03
29	CD137	0.5	1
30	TCRgd	0.5	1
31	CD163	0.5	1
32	CD134	0.5	1
33	Tim3	0.25	0.5
34	CD103	0.0625	0.125
35	CD206	0.25	0.5
36	CD32	0.5	1
37	CD161	0.25	0.5
38	CD39	0.25	0.5
39	CD141	0.125	0.25
40	Lag3	0.5	1
41	CD1c	0.25	0.5
