# Supplementary material for: A Targeted Multi-omic Analysis Approach Measures Protein Expression and Low-Abundance Transcripts on the Single-Cell Level
Source: Cell Rep. Author manuscript; Available in PMC 2020 May 14. (PMC7224638; doi:10.1016/j.celrep.2020.03.063)
Supplement: 2 [file NIHMS1582940-supplement-2.pdf]

Resource

Cell Reports

A Targeted Multi-omic Analysis Approach Measures Protein Expression and Low-Abundance Transcripts on the Single-Cell Level

Graphical Abstract

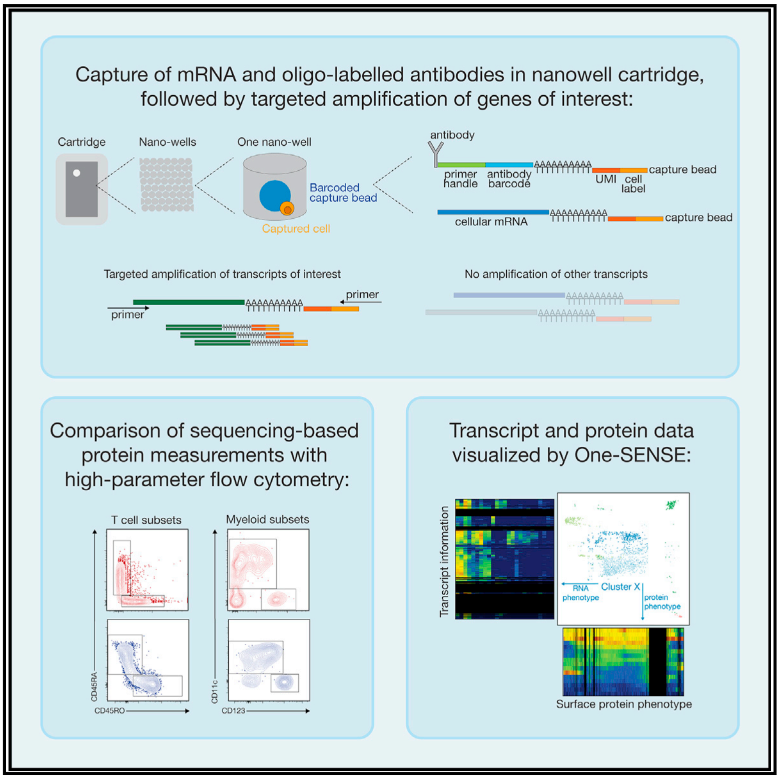

Highlights

- Targeted transcriptomics captures immune cell heterogeneity at a low sequencing depth
- Antibody panels for sequencing-based protein measurement require validation
- Combined protein and transcript measurements highlight T cell heterogeneity
- One-SENSE provides an intuitive visualization tool for protein-transcript datasets

Authors

Florian Mair, Jami R. Erickson, Valentin Voillet, ..., Raphael Gottardo, Evan W. Newell, Martin Prlic

Correspondence

rgottard@fredhutch.org (R.G.),
enewell@fredhutch.org (E.W.N.),
mprlic@fredhutch.org (M.P.)

In Brief

Mair et al. describe a targeted transcriptomics approach combined with surface protein measurement to capture immune cell heterogeneity at a low sequencing depth. One-SENSE is used as a visualization tool to intuitively explore the relationship of protein and transcript expression on the single-cell level.

Check for updates

Mair et al., 2020, Cell Reports *31*, 107499
April 7, 2020 © 2020 The Author(s).
https://doi.org/10.1016/j.celrep.2020.03.063

CellPress

OPEN ACCESS
CellPress

A Targeted Multi-omic Analysis Approach Measures Protein Expression and Low-Abundance Transcripts on the Single-Cell Level

Florian Mair,[1,6] Jami R. Erickson,[1,6] Valentin Voillet,[1] Yannick Simoni,[1] Timothy Bi,[1] Aaron J. Tyznik,[2] Jody Martin,[2] Raphael Gottardo,[1,3,4,*] Evan W. Newell,[1,*] and Martin Prlic[1,5,7,*]
[1]Fred Hutchinson Cancer Research Center, Vaccine and Infectious Disease Division, Seattle, WA 98109, USA
[2]BD Biosciences, La Jolla, CA 92037, USA
[3]Fred Hutchinson Cancer Research Center, Public Health Sciences Division, Seattle, WA 98109, USA
[4]Department of Statistics, University of Washington, Seattle, WA 98195, USA
[5]Department of Global Health and Department of Immunology, University of Washington, Seattle, WA 98195, USA
[6]These authors contributed equally
[7]Lead Contact
*Correspondence: rgottard@fredhutch.org (R.G.), enewell@fredhutch.org (E.W.N.), mprlic@fredhutch.org (M.P.)
https://doi.org/10.1016/j.celrep.2020.03.063

SUMMARY

High-throughput single-cell RNA sequencing (scRNA-seq) has become a frequently used tool to assess immune cell heterogeneity. Recently, the combined measurement of RNA and protein expression was developed, commonly known as cellular indexing of transcriptomes and epitopes by sequencing (CITE-seq). Acquisition of protein expression data along with transcriptome data resolves some of the limitations inherent to only assessing transcripts but also nearly doubles the sequencing read depth required per single cell. Furthermore, there is still a paucity of analysis tools to visualize combined transcript-protein datasets. Here, we describe a targeted transcriptomics approach that combines an analysis of over 400 genes with simultaneous measurement of over 40 proteins on 2 × 10^4 cells in a single experiment. This targeted approach requires only about one-tenth of the read depth compared to a whole-transcriptome approach while retaining high sensitivity for low abundance transcripts. To analyze these multi-omic datasets, we adapted one-dimensional soli expression by nonlinear stochastic embedding (One-SENSE) for intuitive visualization of protein-transcript relationships on a single-cell level.

INTRODUCTION

Pioneering work almost 20 years ago illustrated the ability to study transcript expression at the single-cell level (Chiang and Melton, 2003; Phillips and Eberwine, 1996), but recent advances in microfluidics and reagents allow the high-throughput analysis of transcripts of 10^4 single cells in one experiment (Jaitin et al., 2014; Klein et al., 2015; Macosko et al., 2015). Several methods have been developed for this purpose, and currently the most widely adopted platform is a droplet-based microfluidics system commercialized by 10x Genomics (Zheng et al., 2017).

Although analysis of transcript expression on the single-cell level is a powerful tool to characterize the phenotypic and functional properties of cells, it is imperative to consider the relationship between transcripts and proteins when trying to extrapolate biology. Typically, transcripts are expressed at a much lower level than proteins—for example, murine liver cells have a median copy number of 43,100 proteins but only 3.7 mRNA molecules per gene (Azimifar et al., 2014). Similarly, the dynamic range of expression is much greater for proteins, with copy numbers spanning about 6–7 orders of magnitude, whereas transcript copy numbers span about 2 orders of magnitude (Schwanhäusser et al., 2011). Finally, the correlation of gene expression and protein expression has been estimated to have a Pearson correlation coefficient between 0.4 (Schwanhäusser et al., 2011) and 0.6 (Azimifar et al., 2014). These discrepancies in transcript and protein expression patterns are relevant for the biological interpretation of single-cell transcriptome data but also pose analytical challenges. Suitable approaches are required to visualize the data despite the pronounced differences in abundance and dynamic range of expression.

The parallel measurement of transcript and protein phenotype has been recently reported as cellular indexing of transcriptomes and epitopes by sequencing (CITE-seq) (Stoeckius et al., 2017) or RNA expression and protein sequencing (REAP-seq) (Peterson et al., 2017). These technologies leverage existing single-cell RNA sequencing (scRNA-seq) platforms that use an unbiased whole-transcriptome analysis (WTA) approach that captures cellular mRNA by its poly-A tail and use oligonucleotide-labeled antibodies (carrying unique barcodes) to interrogate surface protein abundance. Typically, current droplet-based WTA approaches result in the detection of ~1,000 unique transcripts per single cell for the transcriptome (with a substantial fraction of these transcripts encoding ribosomal proteins), and antibody panels of up to 80 targets have been reported (Peterson et al., 2017).

This is an open access article under the CC BY license (http://creativecommons.org/licenses/by/4.0/).

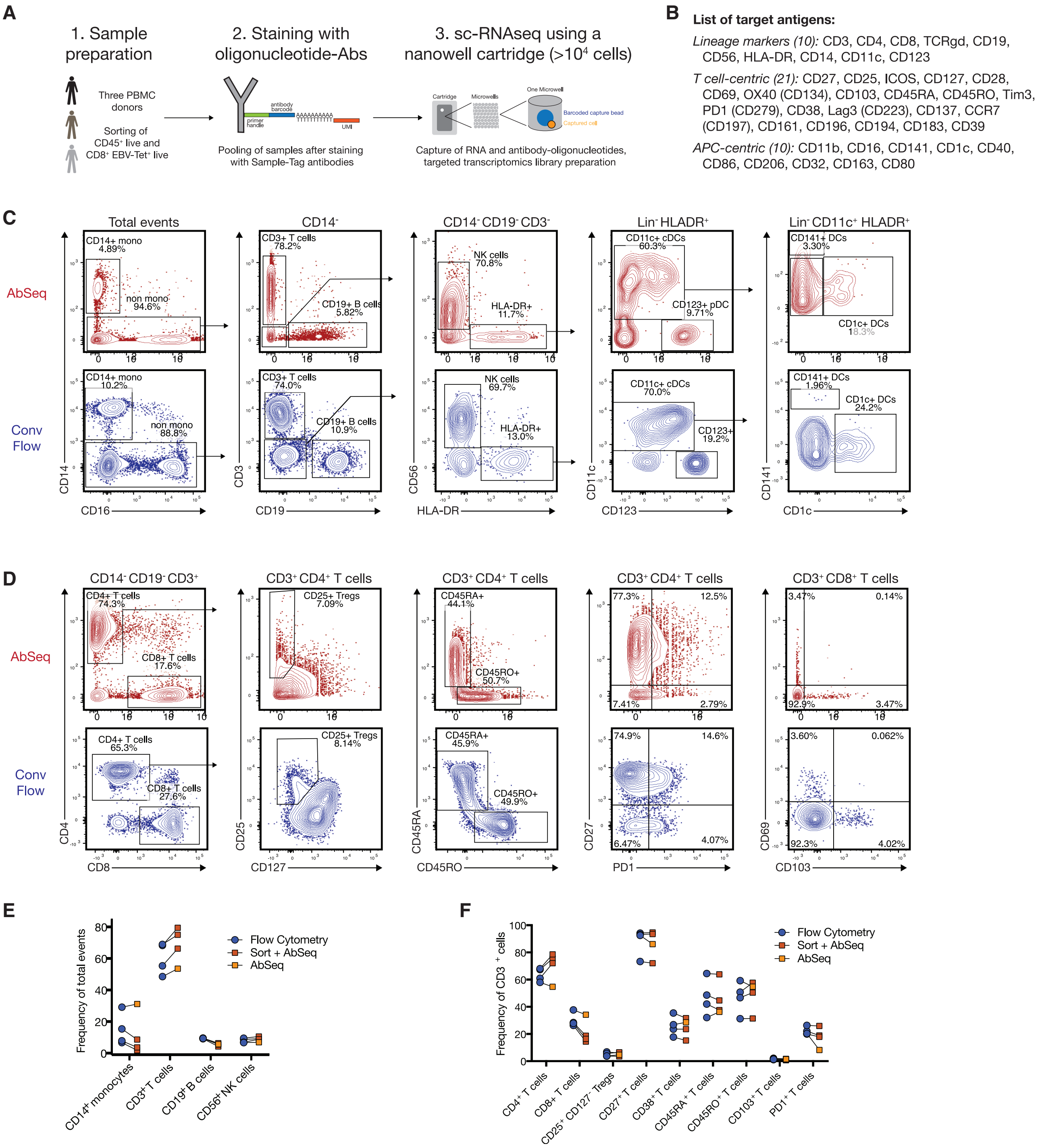

Figure 1. Comparison of Oligonucleotide-Labeled Antibody Probes to High-Dimensional Flow Cytometry

(A) Schematic graph describing the workflow of the experiment. PBMC samples from three donors were split in half, with one aliquot used for the multi-omic workflow and one aliquot used for flow cytometry phenotyping using two 30-parameter panels.

(B) Overview of antibody targets used in both the multi-omic and conventional flow cytometry experiment.

(C) Manual gating of main immune subsets by using the combined AbSeq dataset (top panel, red) and concatenated and down-sampled events (27,000 cells, from three donors) from the conventional (conv) flow cytometry dataset (bottom panel, blue).

(D) Manual gating of various T cell markers by using the combined AbSeq dataset (top panel, red) and concatenated, down-sampled events from the cytometry dataset (bottom panel, blue).

(legend continued on next page)

Although proof-of-principle experiments for this sequencing-based technology have been established, it remains unclear how the antibody detection compares to established flow-cytometry-based assays in different experimental settings with regard to capturing the dynamic range of protein expression and identifying low abundance protein expression. In addition, the combined WTA plus protein approach can quickly become resource intensive given the high number of reads per cell required to achieve library saturation. Finally, droplet-based WTA pipelines may still miss specific transcripts of interest if they are below the limit of detection, with current high-throughput chemistries capturing an estimated 10% of the total cellular mRNA (Zheng et al., 2017).

Here, we report using a high-throughput (>10^4 single cells) targeted transcriptomics approach using nanowells to capture single cells (Rhapsody platform, commercialized by BD Biosciences) (Fan et al., 2015) in combination with oligonucleotide-barcoded antibodies (termed AbSeq). Specifically, we simultaneously interrogated 492 immune-related genes and 41 surface proteins that are commonly used for immunophenotyping. We found that this targeted approach was efficient at detecting low-abundance transcripts while only requiring about one-tenth of the sequencing read depth needed for WTA, indicating that targeted transcriptomics is a sensitive and cost-efficient alternative when the focus is on interrogating defined transcripts. Notably, this approach clearly separated different memory T cell subsets as well as regulatory T cells (Tregs) solely based on transcript information, which is often difficult due to the low amount of mRNA recovered from T lymphocytes (Zheng et al., 2017). Furthermore, we used 30-parameter fluorescent-based flow cytometry to simultaneously measure the same protein targets as in the multi-omic assay. Our data indicate that the validation of oligonucleotide-barcoded antibody panels is necessary for meaningful interpretation of the multi-omic data.

To demonstrate the sensitivity and robustness of the system, we analyzed T cells before and after 1 h of stimulation. We found distinct chemokine expression patterns within the CD8$^+$ T cell population 1 h after stimulation, indicating significant heterogeneity in the response of this compartment.

Finally, to intuitively visualize protein and transcriptome data in a single plot, we adapted one-dimensional soli expression by nonlinear stochastic embedding (One-SENSE), which was originally developed for visualization of mass cytometry data (Cheng et al., 2016). This adaptation allows effective visualization and identification of cellular phenotypes that differ either by transcripts or by proteins. Overall, we provide a methodological toolset for generating high-throughput multi-omic single-cell data with a focus on selected targets at minimal read depth and an analytical tool to visualize these protein and transcript datasets.

RESULTS

Comparison of Oligonucleotide-Labeled Antibody Probes to High-Dimensional Flow Cytometry

For our reference dataset, we used peripheral blood mononuclear cells (PBMCs) from three healthy control subjects carrying the HLA-A*02:01 allele, which allowed isolation of Epstein-Barr Virus (EBV)-specific CD8$^+$ T cells by using an EBV-tetramer reagent (EBV epitope YVLDHLIVV) (Dunne et al., 2002). To ensure sufficient cell numbers of these rare, antigen-specific T cells, we enriched tetramer-positive T cells by fluorescence-activated cell sorting (FACS). In parallel, we sorted CD45$^+$ live leukocytes from PBMCs (Figure 1A). Moreover, to minimize batch effects during subsequent staining with 41 oligo-nucleotide-labeled antibodies (Figure 1B), we used a multiplexing protocol with barcoded cell-hashing antibodies (Stoeckius et al., 2018). All samples were processed simultaneously using the Rhapsody platform, a nanowell-based cartridge system (Fan et al., 2015) for scRNA-seq with a targeted approach focusing on 492 immune-relevant transcripts (397 pre-defined targets complemented by 95 experiment-specific targets, full list provided in Table S1). Following quality control and removal of multiplets, we recovered 27,258 cells from the sequencing data, which were evenly distributed across the three different donors (donor 1: 8,984 cells, donor 2: 8,956 cells, donor 3: 9,318 cells).

First, we wanted to assess whether the surface protein phenotypes as defined by sequencing match known biology. For this, we designed two optimized 30-parameter immunophenotyping panels (adapted from Mair and Prlic, 2018) covering the same 41 protein targets in an overlapping fashion. We used these panels to stain whole unsorted PBMC samples from the same 3 donors, down-sampled the cytometry data to 27,000 cells, and used biaxial gating to identify the main immune lineages of the myeloid compartment (Figure 1C) as well as the lymphoid compartment (Figure 1D). All populations were present at comparable frequencies in the two different datasets, with myeloid cells showing slightly lower abundance in the experiment that included cell sorting (Figures 1E and 1F). Notably, even low-abundance cell populations, such as CD1c$^+$ conventional dendritic cells (cDCs) and cross-presenting CD141$^+$ cDCs, were clearly identified by their surface protein phenotype (Figure 1C). Furthermore, the oligonucleotide-labeled antibodies allow discrimination of the CD45 splice variants CD45RO and CD45RA, which are used to subset T cells and cannot be distinguished by 3′ transcriptomic analysis alone.

However, for the anti-T cell receptor $\gamma\delta$ (TCR$\gamma\delta$) reagent we used, discordant patterns were observed when comparing the expression to conventional flow cytometry, despite using the same antibody clone (Figure S1A). This was not immediately evident when visualizing the data on a heatmap (Figure S1B),

(E) Quantification of main immune subsets by using AbSeq and flow cytometry either with prior cell sorting (red squares) or using AbSeq without prior cell sorting (orange squares).

(F) Quantification of main T cell populations and selected phenotyping markers from two independent experiments using AbSeq and flow cytometry either with prior cell sorting (red squares) or using AbSeq without prior cell sorting (orange squares).

See also Figure S1 and Table S1 for full list of genes.

emphasizing the need for careful reagent validation for sequencing-based protein measurements. Thus, we did not analyze γδ T cells separately for the rest of our study. Furthermore, the anti-CCR7 antibody clone used in the AbSeq experiment delivered sub-optimal resolution (data not shown). A detailed list of antibody clones and used concentrations can be found in the STAR Methods (Table S4).

Targeted Transcriptomics Captures the Major PBMC Lineages Similar to Whole-Transcriptome Approaches

Next, we wanted to assess how well a targeted transcriptomics approach can identify immune cell heterogeneity compared to a commonly used WTA pipeline (Zheng et al., 2017). For this, we used a single donor and compared the resulting populations after graph-based clustering of the transcript data by using the R package Seurat implementation of PhenoGraph at standard resolution settings (Butler et al., 2018; Levine et al., 2015; Figures 2A and S2A). For visualization, we used uniform manifold approximation and projection (UMAP), a dimensionality reduction approach that has recently been adopted for single-cell data (Becht et al., 2019; McInnes et al., 2018). Overall, the targeted transcriptomic approach using 492 genes revealed a similar resolution of known immune subsets in the peripheral blood relative to WTA (Figures 2B and S2B). In particular, $CD4^+$ T cells and $CD8^+$ T cells separated well, and we observed Tregs expressing FOXP3 and CTLA4 as a separate cluster (Figure 2B). For verification of this Treg cluster, we used the corresponding protein signature, which showed high expression of CD25 and low expression of CD127 (Figure 2C). To determine if we could extract these same clusters from a WTA set, we performed clustering of a publicly available WTA reference dataset at different resolution parameters (Figures S2B and S2C).

Next, to obtain a relative measure of detection efficiency, we calculated the average number of transcripts per cell both for the targeted transcriptomics as well as the WTA dataset from the same donor. Around 75% of the assayed genes showed equal or slightly superior detection efficiencies (Figure 2D). However, although there was a small subset of transcript targets that showed considerably higher detection efficiency (including FOXP3 and CTLA4, identifying the Treg cluster), around 34 targets (including PDCD1) were detected at less than 30% of their level in the WTA dataset (full list and calculation provided in Table S2). These differences are likely due to the different amplification efficiencies using multiplexed targeted primers (Rhapsody) versus primers designed to recognize the template switch-oligo (10x Genomics).

Next, we compared the gene expression pattern for four phenotypically similar clusters (as defined by their transcript profile) in our WTA and the targeted transcriptomics dataset from the same donor, and we included the same clusters from a publicly available WTA reference dataset containing more than 8,000 cells (Figure 2E). Visualizing the top differentially expressed genes (as identified by model-based analysis of single-cell transcriptomes [MAST] in the targeted dataset, see STAR Methods) of these four clusters on a heatmap yielded very similar patterns, suggesting that targeted transcriptomics with an immune-focused primer panel faithfully recapitulates cellular heterogeneity of PBMCs at the single-cell level.

To further compare our donor-matched datasets, we used MAST to identify all differentially expressed genes for each cluster with a log-fold change of >0.25 in both datasets. For three clusters ($CD56^+$ natural killer [NK] cells, $CD4^+$ T cells, and $CD14^+$ monocytes) we compiled a list of genes that were shared between the two datasets or that were only present in the WTA data and only present in the targeted transcriptomics data (full list of genes available in Table S3). Visualizing all overlapping genes for a representative cluster (the NK cell cluster) in both datasets again displayed very similar patterns (Figure S2D). In the case of the NK cell cluster, there were 73 additional genes identified only in the WTA data (heatmap representation of the top 23 of these is shown in Figure S2E). To gain insight into how well biological processes are captured with a targeted set of genes, we performed Gene Ontology (GO) analysis, showing that the majority of GO terms were detected in either dataset, with some GO terms (e.g., GO-0051179, ‘‘localization’’) only being found in the WTA dataset (Figure S2F).

Finally, to directly assess the effect of different read depths on the resolution of protein and transcript signals in the AbSeq dataset, we analyzed PBMCs of a different donor with a total of approximately 27,000 reads/cell (approximately 18,000 reads/cell for the antibody library, 9,000 reads/cell for the transcript library; see STAR Methods for list of all read depths) and subsampled the number of reads during processing of the raw data to 20% (approximately 4,000 reads/cell for the antibody library, 2,000 reads/cell for the transcript library) and 10%. Visualization of the resulting clusters on a UMAP plot as well as the top differentially expressed genes on a heatmap revealed no major differences between using 100% or 20% of the reads (Figure S3A). For the protein signal, differences only became apparent when using 10% of the reads, which resulted in a noticeable loss of signal intensities (Figure S3B). For protein targets that exhibited bimodal distributions, we calculated the absolute number of molecules detected, revealing a noticeable drop in signal intensity when down-sampling to 10% of the reads (Figure S3C).

Overall, we conclude that the panels used in our assay targeting at least 2,000–4,000 reads/cell for the transcript portion of the library (which is approximately one-tenth of the typical read depths used for WTA approaches) (Zheng et al., 2017) and at least 200-400 reads/antibody/cell for the antibody portion of the library deliver sufficient resolution.

Multi-omic Analysis Identifies Canonical Memory T Cell Populations and Allows the Study of Rare-Antigen-Specific $CD8^+$ T Cells

We next performed an in-depth analysis of the $CD8^+$ T cell compartment. First, we visualized protein and RNA data collected from total $CD45^+$ live cells from PBMCs from three patents on separate UMAP plots. We found that cells from different donors comingled and separated by cell type rather than by donor, suggesting that there was no cluster driven by a single donor (Figure 3A, left panel). Also, the two experimental batches (cartridges) did not show significant separation on a UMAP plot calculated using transcripts or proteins (Figure 3A, right panel).

Notably, protein information overlaid on the transcript-generated UMAP plot allowed accurate identification of all main

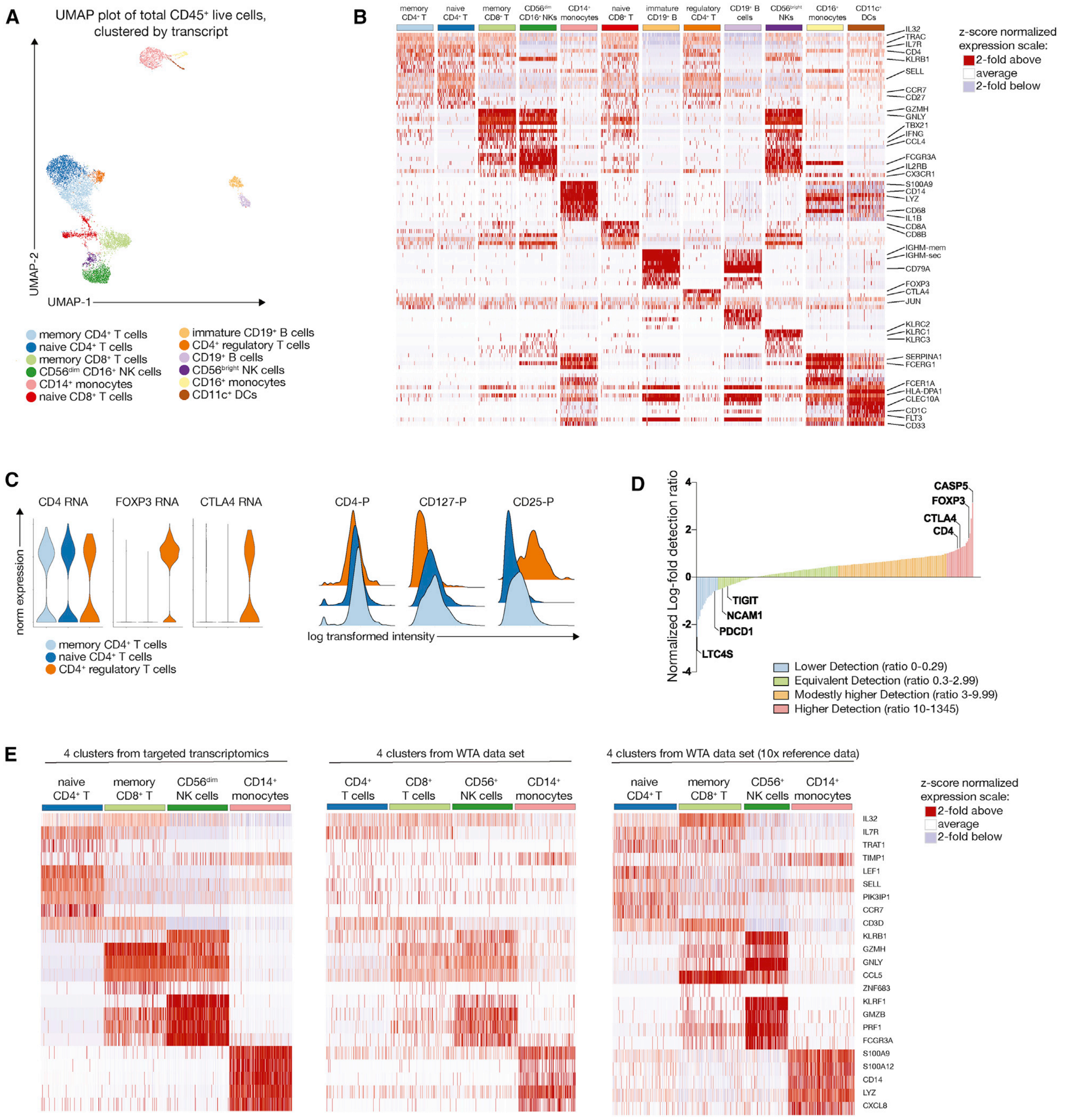

Figure 2. Targeted Transcriptomics Captures the Major PBMC Lineages Similar to Whole-Transcriptome Approaches

(A) Graph-based clustering of the transcript data from one representative donor (8,843 cells) is shown on a UMAP (uniform manifold approximation projection) plot. Clusters have been annotated by expression of key lineage genes.

(B) The top 10 differentially expressed genes for each cluster were identified using the Seurat implementation of MAST (model-based analysis of single-cell transcriptomes) and visualized on a heatmap after *Z* score normalization. Cluster names are shown in the same color scheme as in (A).

(C) Expression of the indicated transcripts and proteins on the three different CD4+ T cell clusters, highlighting the $CD25^{+}$ $CD127^{low}$ Treg cluster (orange).

(D) Relative detection ratio of all detected transcripts relative to a whole-transcriptome dataset from the same donor. Genes are manually assigned into four different groups according to their relative detection ratio.

(E) Expression pattern of the top 5 differentially expressed genes for each cluster (as identified by MAST on the targeted transcriptomics dataset) for 4 representative main immune populations on the targeted data (left), whole-transcriptome data from the same donor (middle), and a publicly available whole-transcriptome reference dataset (right).

See also Figures S2 and S3.

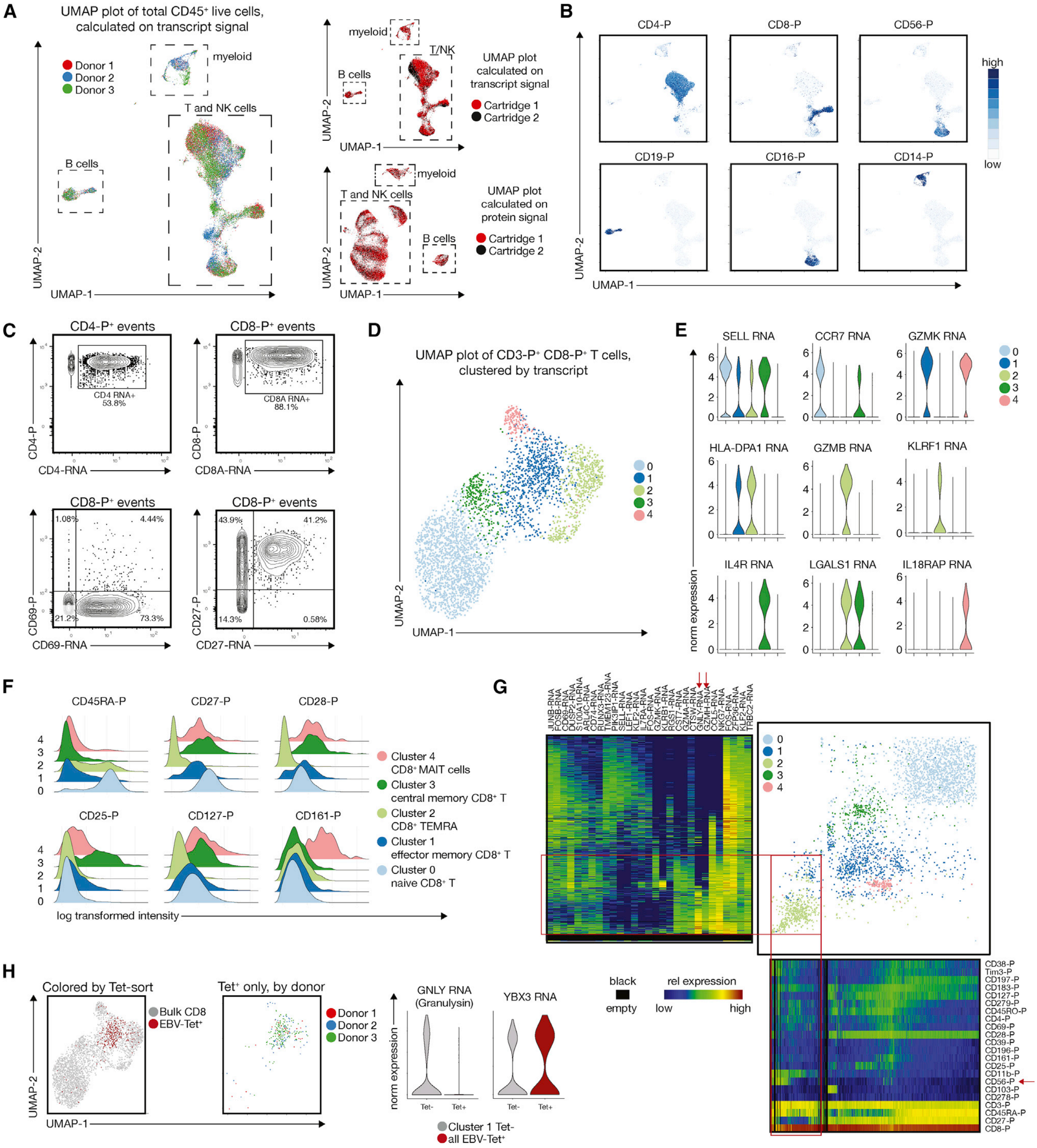

Figure 3. Multi-omic Targeted Transcriptomics Identifies Canonical Memory T Cell Populations and Allows the Study of Rare-Antigen-Specific CD8⁺ T Cells

(A) UMAP plots calculated as indicated and colored by donor (left) or by cartridge run (right) show that there are no major clusters driven from the different experimental runs or individual donors.

(B) Example UMAP plots (calculated on transcript) representing the expression of the main immune lineage protein markers, which allow the unequivocal identification of $CD4^+$ and $CD8^+$ T cells, $CD19^+$ B cells, and $CD14^+$, as well as $CD16^+$ myeloid cells.

(C) Example bivariate plots showing the poor correlation of transcript and protein levels for CD4 and CD69 and good correlation for CD8 and CD27. Protein signal is plotted on the y-axis, and transcript signal on the x-axis.

(legend continued on next page)

immune clusters (Figure 3B), which is not necessarily the case when using transcript information for the corresponding lineage markers. This is exemplified by biaxial plots showing protein signal on the y-axis and transcript signal on the x-axis (Figure 3C). Although CD8A, transcript, and protein are co-expressed in most cells, only half of the CD4-protein$^+$ (throughout the manuscript abbreviated as CD4-P) cells contained detectable CD4-transcript. On the contrary, CD69-RNA (plotted on the x-axis) was detected across a large number of CD8$^+$ T cells, but as expected only a few CD8$^+$ T cells in the peripheral blood express the CD69 protein (CD69-P, plotted on y-axis) on their surface. For CD27, we observed a higher correlation between transcripts and proteins (Figure 3C). Overall, these observations emphasize the importance of the parallel measurement of proteins and transcripts to faithfully study T cell biology.

Next, we continued our analysis of CD8$^+$ T cells (defined by surface protein expression) using SCAMP (selected clustering annotated using modes of projections) (Greene et al., 2018). Unbiased graph-based clustering using transcript information suggested the presence of 5 distinct cellular clusters (Figure 3D). Visualization of the top differentially expressed genes, such as SELL (encoding CD62L), CCR7, and GZMB, suggested that these 5 clusters reflect canonical naive and memory T cell populations (Sallusto et al., 1999; Figure 3E). Additionally, our data allowed identification of CD8$^+$ mucosal-associated invariant T (MAIT) cells, which express high levels of Interleukin-18 Receptor Accessory Protein (IL18RAP) and Tumor Necrosis Factor (TNF) (Slichter et al., 2016; Mori et al., 2016). We confirmed the resemblance of these populations by surface protein expression (Figure 3F), with central memory CD8$^+$ T cells expressing low levels of CD45RA protein and high levels of both CD27 and CD28 protein (Sallusto et al., 2004; Hamann et al., 1997). Importantly, the splice variants CD45RO and CD45RA cannot be distinguished by analyzing transcripts alone, highlighting the added value of combined protein and transcript analysis.

To visualize the correspondence between transcript and protein expression in the multi-omic dataset, we adopted One-SENSE, which has originally been developed for visualizing mass cytometry data (Cheng et al., 2016). We mapped cells by separately plotting proteins and transcripts each on to a single UMAP dimension, similar to a recently published one-dimensional t-stochastic neighbor embedding (t-SNE) representation for scRNA-seq data (Linderman et al., 2019). The combined plot shows the overall distribution of protein expression profiles in the x-axis and the top differentially expressed gene profiles on the y-axis. Aligned heatmaps that represent median expression with bins of cells are provided to annotate the one-dimensional UMAP protein and gene expression profiles (blue: low expression, red: high expression). Black areas in the heatmaps refer to areas of the One-SENSE plot that are devoid of cells. This approach allows easy identification of cellular clusters that are similar by transcripts but separated by proteins and vice versa (Figure 3G). One example for this is highlighted in Figure 3G (red box and arrow), where cluster 2 (light green, TEMRA cells) is relatively homogeneous by transcript but can be separated by CD56 protein expression. A fraction of cells between cluster 1 (dark blue, effector memory CD8$^+$ T cells) and 2 (light green, TEMRA) shares the same protein signature but can be distinguished by GNLY and GZMH expression (Figure 3G, red box and arrows). Varying degrees of concordance and ability to discriminate cellular subsets from gene and protein expression profiles can be seen across this plot.

To determine if targeted transcriptomics is amenable for studying rare-antigen-specific T cell populations, we analyzed CD8$^+$ T cells recognizing the HLA-A*02-restricted EBV epitope YVLDHLIVV (Dunne et al., 2002). Visualization on the UMAP plot revealed remarkable similarity of EBV-specific T cells across all three donors (Figure 3H), with most of the cells grouped within the effector memory CD8$^+$ T cell cluster. However, relative to the EBV-nonspecific memory T cell cluster, the EBV-Tet$^+$ T cells showed a significant downregulation of the Granulysin transcripts and an upregulation of YBX3, an RNA binding protein whose function has not been defined in T cells but has recently been shown to be a critical regulator for the stability of specific mRNAs (Cooke et al., 2019).

Overall, these data show that a combined analysis of targeted transcriptomics and protein expression by sequencing is a valuable approach for studying T cell subsets and could be used as a resource-efficient tool for studying T cell responses in human disease.

Short-Term Stimulation of T and NK Cells Reveals Chemokine Heterogeneity and a Disconnect with the Early Activation Marker CD69

Cytokines and chemokines are the quintessential effector molecules of T cells, and the existence of specific T cell subsets that are poised to produce certain cytokines has been the subject of intense research over the past decade (van den Broek et al., 2018; Zhou et al., 2009). To test whether multi-omic single-cell analysis can provide additional insight, we purified T and NK cells and stimulated them for 1 h with phorbol-myristate-acetate (PMA) and ionomycin. We probed early transcriptional changes with a T-cell-centric-targeted transcriptomic approach covering 259 genes. Transcripts encoding for IFNG, FASL, and ICOS exhibited robust upregulation in the stimulated versus unstimulated sample (Figure 4A), as was the case for CD69, a commonly used protein marker for early T cell activation (Figure 4B). Notably, when we analyzed cytokine expression relative to the surface

(D) UMAP plot and graph-based clustering of the CD3$^+$ CD8$^+$ CD4− T cell compartment, revealing 5 distinct populations.

(E) Violin plots showing some of the top differentially expressed genes identified by MAST for each of the 5 clusters in (D).

(F) Protein signatures of the 5 clusters identified canonical naive and memory CD8$^+$ T cell subsets, including CD8$^+$ mucosal-associated invariant T cells (MAIT cells).

(G) One-SENSE plot depicting protein expression heatmap along the x-axis, and transcript expression heatmap of the top differentially expressed genes along the y-axis.

(H) Identification of EBV-specific CD8$^+$ T cells relative to all CD8$^+$ T cells, and expression pattern of two differentially expressed genes between tetramer-positive cells and tetramer-negative cells in the effector memory cluster 1.

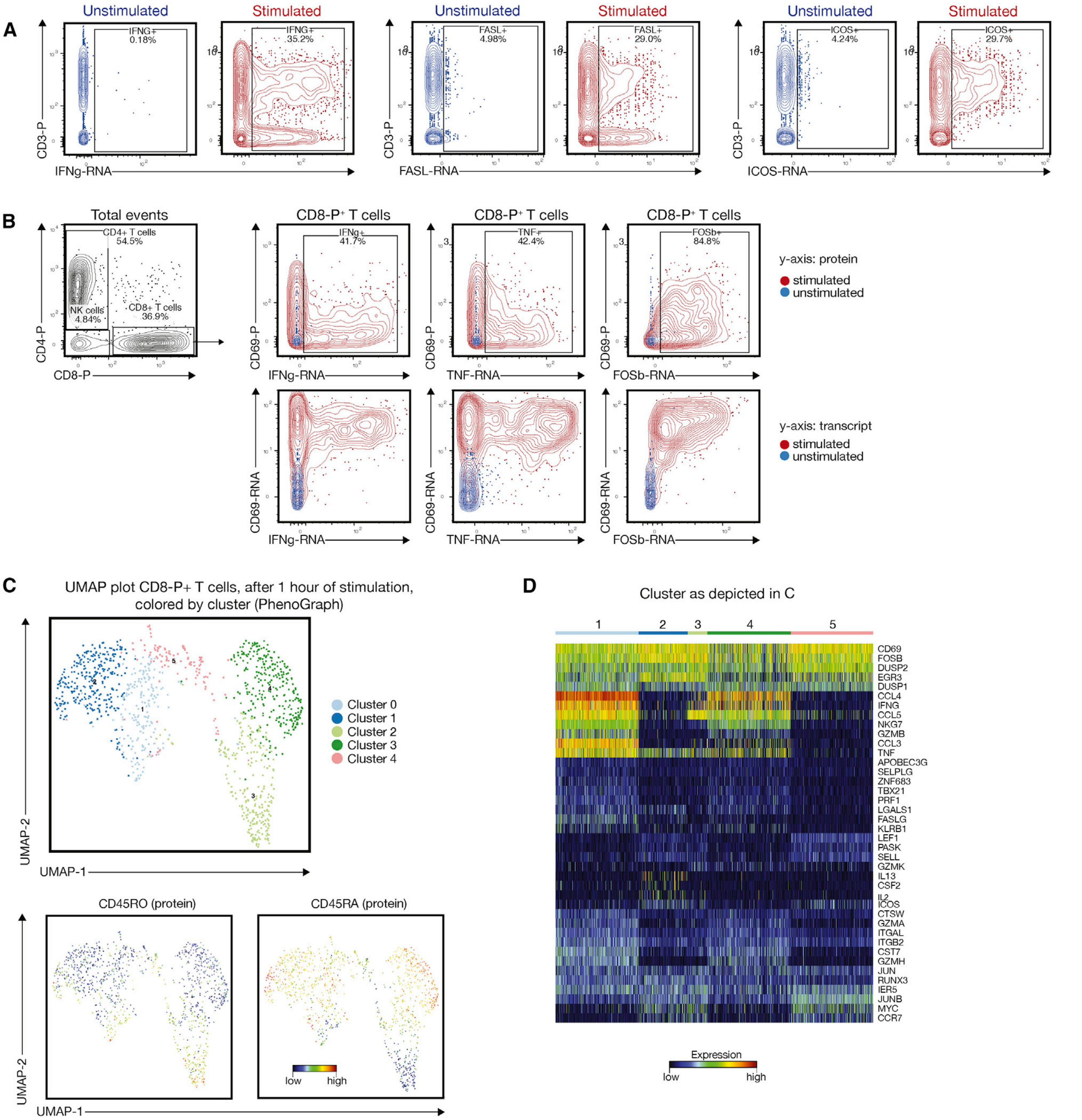

Figure 4. Multi-omic Analysis of the T and NK Cell Compartment 1 h after Stimulation

(A) Representative plots showing the upregulation of selected effector transcripts, such as IFNG, FASL, and ICOS, after stimulation (red) relative to unstimulated cells (blue).

(B) Disconnect between surface protein expression of the early activation marker CD69 and IFNG and TNF transcript within CD8 protein$^+$ T cells. Blue overlay indicates unstimulated cells, and red indicates stimulated cells.

(C) UMAP plot of stimulated CD8-protein$^+$ T cells showing five phenograph-defined clusters and corresponding CD45RA and CD45RO protein expression.

(D) Heatmap showing the expression of key effector transcripts within the clusters identified in (C).

protein expression of CD69 on CD8$^+$ T cells, we observed that both IFNG as well as TNF transcripts were primarily expressed in CD69-transcript-positive, but CD69-protein-negative cells, whereas the FOSB transcript (encoding for a subunit of the transcription factor AP-1) was co-expressed with the CD69 protein (Figure 4B).

Data projection on a UMAP plot after phenograph-based clustering (Levine et al., 2015) suggested the presence of five different clusters (Figure 4C). Protein expression patterns for CD45RA and CD45RO highlight the distribution of naive, effector, and memory T cells within this plot (Figure 4C). A heatmap visualization of the most highly expressed transcripts show that these five clusters are primarily defined by the differential expression of CCL3, CCL4, IFNG, TNF, and various granzymes (Figure 4D). Overall, this analysis reveals considerable functional diversity within the $CD8^+$ T cell compartment that is detectable 1 h after stimulation. Given the nature of PMA- and ionomycin-induced activation, this finding likely reflects intrinsic functional heterogeneity.

Multi-omic Analysis of the Peripheral Myeloid Compartment Reveals Inflammatory Subsets Not Captured by Surface Protein Phenotype

Next, we wanted to determine whether the targeted transcriptomics approach can also be used for other immune populations that are not as well studied as T cells. During the past decade, it has become evident that the myeloid cell compartment is complex in terms of cellular heterogeneity (Guilliams et al., 2014; See et al., 2017; Villani et al., 2017) and that commonly used bone-marrow-derived differentiation protocols do not faithfully capture the phenotype of myeloid cells *in vivo* (Guilliams and Malissen, 2016; Helft et al., 2015). Thus, we tested how well targeted transcriptomics could dissect the heterogeneity of the peripheral myeloid compartment. Unbiased clustering using transcripts suggested the presence of 5 different populations (Figure 5A), with clear separation of CD14 and CD16 protein expression (Figure 5B). As expected, visualization of the top differentially expressed genes (Figure 5C) as well as key surface proteins (Figure 5D) mapped these clusters to $CD123^+$ plasmacytoid DCs (pDCs), $CD1c^+$ cDCs, $CD16^+$ monocytes, and $CD14^+$ monocytes. We used One-SENSE to further explore the relationship between cluster 0 and 1, revealing that these two populations were very similar in terms of surface protein profile ($CD14^+$ $CD16^-$ classical monocytes) but were separated by a specific set of transcripts encoding for pro-inflammatory cytokines and chemokines (Figure 5E). We used MAST to confirm that these transcripts were differentially expressed (Finak et al., 2015), with higher expression of CXCL3 and CCL4 (also known as MIP-1b, a chemoattractant for natural killer cells) in cluster 1 (Figure 5F). These cells in cluster 1 could be related to a very recently defined inflammatory subset of $CD14^+$ $CD163^+$ myeloid cells (Dutertre et al., 2019). Thus, combining protein and transcriptome data allowed us to observe multiple functional subsets within the peripheral $CD14^+$ myeloid population, which were not apparent by surface marker expression alone. In summary, these data highlight that targeted transcriptomics can be used for exploratory studies of different immune compartments.

DISCUSSION

Current efforts in the field of single-cell analysis focus on the integrative measurement of multiple modalities per cell. Ultimately, being able to analyze DNA sequence, genome accessibility status, transcript, regulatory RNAs, and protein expression all together would allow a holistic understanding of cellular function, but this has not yet been achieved (Stuart and Satija, 2019). Arguably one of the most important steps on this trajectory has been the ability to combine protein and transcript measurements by sequencing at the single-cell level by using high-throughput methods (Peterson et al., 2017; Stoeckius et al., 2017). However, with increased cell numbers, these combined measurements can quickly become resource intensive, mostly due to the high number of sequencing reads that are required per cell. Moreover, to fully leverage the advantage of multi-omic single-cell analysis approaches, it is imperative to collect large cell numbers to adequately represent low-abundance cellular populations, such as antigen-specific T cells or antigen-presenting cells (APCs). This can be exemplified with DC subsets; $CD1c^+$ cDCs typically represent only 0.1%–0.5% of cells in the peripheral blood, i.e., to capture 50 cells without prior enrichment requires sequencing a minimum of 10,000 cells. However, even 50 cells might be insufficient to extrapolate functional potential if there is pronounced heterogeneity within such a rare population.

The targeted transcriptomic approach that we describe here provides an alternative platform that significantly lowers the number of reads required for sequencing saturation of transcripts compared to WTA approaches but still provides valuable information on 492 immune-centric genes. Notably, the targeted workflow avoids the significant number of reads used by transcripts encoding ribosomal proteins, which provide limited insight in the context of specifically assessing immune cell function. In combination with the lower number of target genes relative to WTA, this contributes to requiring less reads to achieve library saturation. It is important to keep in mind that a targeted approach sacrifices the unbiased nature and breadth of WTA. However, many immunological applications are centered around a set of critical immune effector molecules, such as cytokines, chemokines, or transcription factors, which are comprehensively covered by a targeted gene panel. Because genes of interest can be selected for such a panel, this approach allows tailoring toward a specific research question.

Our experiments suggest that a targeted workflow can, in some cases, deliver high sensitivity when it comes to detecting relatively low abundance transcripts, but we also found a set of genes that was underrepresented relative to WTA. This is probably related to the sub-optimal amplification efficiencies of certain targeted primer pairs. Modifying primer design should be sufficient to improve detection sensitivity of these genes. Overall, in many experimental setups it might be beneficial to combine both approaches: first, use a WTA platform to identify potentially unknown transcripts and then use a targeted approach (ideally tailored toward gene sets of interest) for profiling larger cell numbers or interrogating cellular responses to specific stimuli. Here, we provide proof-of-concept data that as early as 1 h after stimulation $CD8^+$ T cells show heterogeneous patterns of chemokine expression. Comprehensive chemokine and cytokine profiling of T cells after a very short stimulus could be very valuable to gain additional insights into their function, e.g., in the context of cancer immunotherapy (Nagarsheth et al., 2017).

The rather low number of reads per cell required for targeted transcriptomics makes the approach very suitable for combined

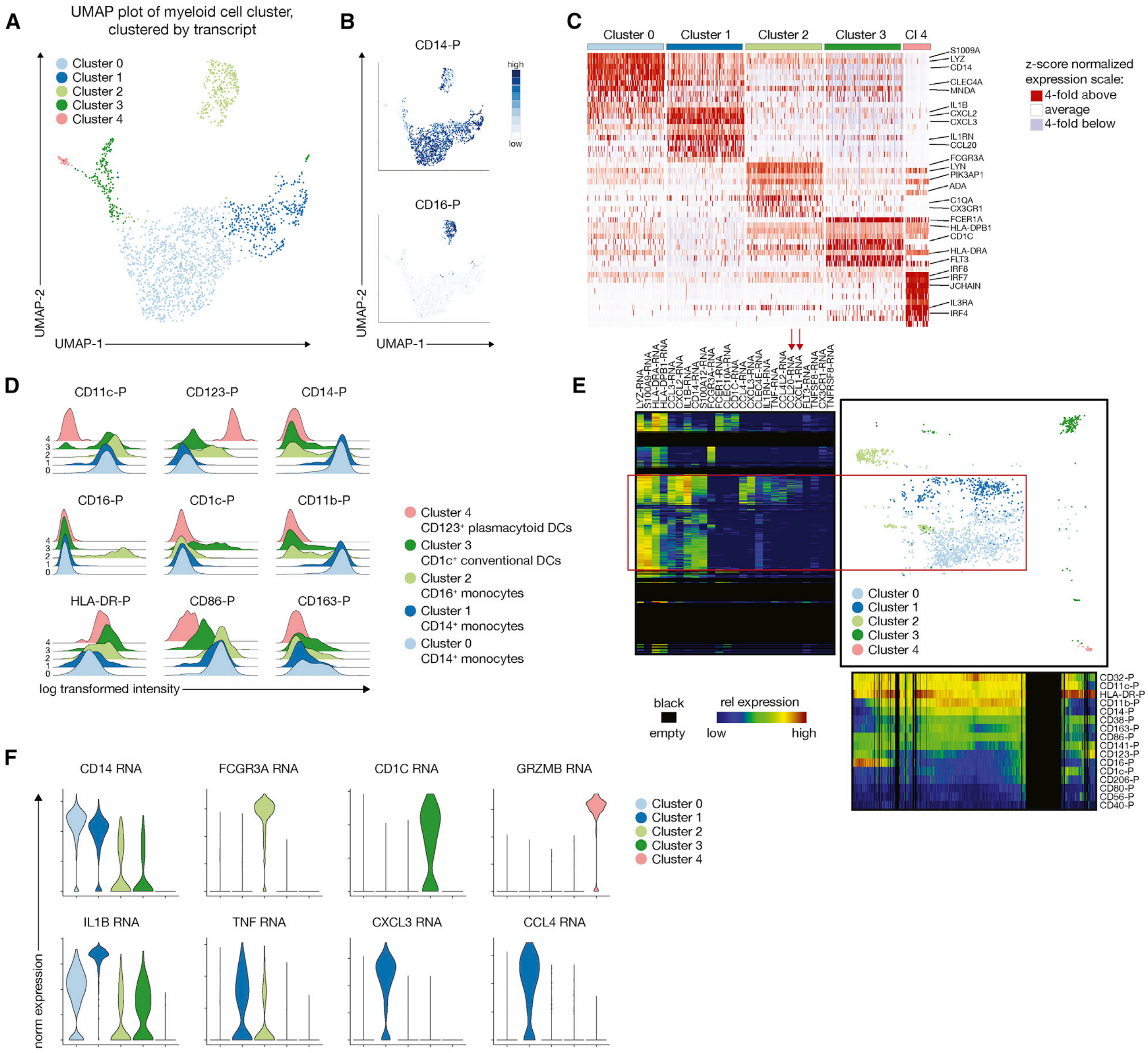

Figure 5. Combined Protein and Transcript Phenotyping of the Peripheral Myeloid Compartment Reveals Inflammatory Subsets Not Captured by Surface Protein Phenotype

(A) UMAP plot and graph-based clustering of the peripheral non-T/non-NK/non-B cell compartment, revealing 5 distinct populations.

(B) Heatmap overlay of CD14 and CD16 protein expression.

(C) Heatmap of the top differentially expressed genes identified by MAST for each of the 5 clusters highlighted in (A).

(D) Protein signatures of the 5 clusters identifies canonical CD123^{+} plasmacytoid DCs, CD1c^{+} conventional DCs, and CD16^{+} monocytes but two of the clusters map to CD14^{+} monocytes.

(E) One-SENSE plot depicting protein expression heatmap along the x-axis and transcript expression heatmap of some of the top differentially expressed genes along the y-axis. Red box and arrows highlight the differentially expressed genes between cluster 0 and 1.

(F) Violin plots showing key genes of the respective myeloid population (top panel) and differentially expressed genes between cluster 0 and 1, suggesting the presence of an inflammatory subpopulation within CD14^{+} CD16^{-} monocytes that expresses high levels of IL1B, TNF, CXCL3, and CCL4.

profiling of transcript and protein expression for larger numbers of cells. This is particularly relevant in the context of T cell biology, where well established T cell subsets, such as memory T cells and Tregs can be difficult to identify in some droplet-based scRNA-seq studies solely on the basis of transcripts (Zheng et al., 2017). This has been attributed to the fact that lymphocytes contain a relatively low amount of mRNA, which, in combination with the inherent drop-out rate of scRNA-seq protocols, fails to detect some low-abundance transcripts that define these cellular subsets (Stuart and Satija, 2019). This issue can be alleviated by measuring surface protein markers, such as the splice variants CD45RA and CD45RO, which have been well

studied in the context of naive and memory T cells, or the interleukin-2 (IL-2) receptor alpha chain (CD25) and IL-7 receptor (CD127) for the distinction of Tregs.

In addition, the parallel measurement of surface protein phenotypes allows linking novel cellular clusters (that are defined solely by transcript) with previously characterized cellular subsets that are defined by surface protein phenotype only. Finally, the combined measurement approach can be useful to identify targets with a significant disconnect between transcript and protein expression, as we observed for CD69.

The development of novel technologies can sometimes outpace our ability to validate platforms and reagents. Given that typical single-cell sequencing experiments require complex pre-processing steps and are often visualized using dimensionality reduction techniques, such as UMAP or t-SNE, there is a disconnect between the actual raw data and the interpretation of final heatmaps. Although this might be less of a problem for transcript counts, antibody-based probes require careful validation, which is highlighted by our observation that even the same antibody clone can yield a different result in a multi-omic sequencing experiment relative to conventional cytometry. In general, we found that sequencing-based expression patterns for proteins that show clear bimodal expression (such as CD3, CD14, or other lineage markers) were easy to interpret, but making a positivity call could become challenging for protein markers with low or continuous expression patterns (such as PD1, Tim-3, or IL-7Rα). Distinguishing signal from noise for proteins with rather low expression levels is further complicated by the fact that antibody-based probes inevitably yield some degree of unspecific binding, thus introducing a background signal. As sequencing-based protein measurements become more common, it is important to reach a consensus in the field regarding standard quality-control practices. Reagents need to be carefully tested and validated, preferably with parallel deposition of the validation data in public databases, and analysis approaches should rely on algorithm-assisted determination of positivity cutoffs, e.g., by using SCAMP (Greene et al., 2018) or other tools developed for this purpose.

Additional work is needed to assess whether more complex antibody panels require different sequencing depths relative to our study. We are not providing a general recommendation regarding an optimal sequencing depth to measure protein expression because the required read depth will depend on the oligo-to-antibody ratio of a given reagent and the relative abundance of the target epitopes in an experimental sample. Specifically, samples that are stained with antibody-oligo conjugates that consume a large fraction of the sequencing reads (such as, e.g., HLA-DR, CD4, and CD8) may require additional read depth to ensure that low-abundance protein targets are not missed due to a lack of reads. Finally, it is important to note that sequencing-based antibody measurements are only semiquantitative and cannot be used to determine absolute surface protein counts, which is, in part, due to the limited efficiency of current single-cell platforms regarding oligonucleotide capture and subsequent cDNA synthesis (Zheng et al., 2017).

Ultimately, to advance our understanding of biology, the field relies on innovative approaches to analyze and visualize high-dimensional data (Butler et al., 2018; Cao et al., 2019; Stuart and Satija, 2019). Due to the complexity of the data and different expression scales, this presents a challenge for combined protein-transcript datasets. To alleviate this problem, we have adopted an analysis approach successfully used for high-dimensional cytometry data, One-SENSE (Cheng et al., 2016). By visualizing the top differentially expressed genes in one dimension relative to the measured protein phenotypes, this method allows easy dissection of cells that are similar in transcript phenotype but different in protein phenotype and vice versa. This visualization approach will be a useful tool for biologists to explore future multi-omic datasets to extract biological meaning from these complex multi-dimensional data.

STAR★METHODS

Detailed methods are provided in the online version of this paper and include the following:

- KEY RESOURCES TABLE
- LEAD CONTACT AND MATERIALS AVAILABILITY
- EXPERIMENTAL MODEL AND SUBJECT DETAILS
  - Primary Cells
- METHOD DETAILS
  - T cell stimulation assay
  - Flow Cytometry and Cell sorting
  - Targeted Transcriptome and protein single-cell library preparation and Sequencing
  - Whole Transcriptome single-cell library preparation and sequencing
- QUANTIFICATION AND STATISTICAL ANALYSIS
  - Cell Ranger processing for WTA data
  - Seven Bridges processing for targeted transcriptomics data
  - Seurat workflow for targeted and WTA data
  - Data processing for One-SENSE and generation of FCS files
- DATA AND CODE AVAILABILITY

SUPPLEMENTAL INFORMATION

Supplemental Information can be found online at https://doi.org/10.1016/j.celrep.2020.03.063.

ACKNOWLEDGMENTS

We would like to thank the HIV Vaccine Trial Network (HVTN) for providing samples and for access to their flow cytometry instrumentation (in particular Dr. Stephen de Rosa), the Flow Cytometry Shared Resources Core of the FHCRC (in particular Andrew Berger), and the Genomics Shared Resources Core of the FHCRC for sequencing. We thank members of the Newell and Prlic labs for critical discussion. This work was supported by NIH grants R01AI123323 (to M.P.) and 5U19AI128914 (to R.G.). E.W.N., Y.S., and T.B. were supported by Fred Hutchinson Cancer Research Center New Development funding and by the Andy Hill Research Endowment. F.M. was supported through The American Association of Immunologists (AAI) Intersect Fellowship Program for Computational Scientists and Immunologists. J.R.E. was supported by National Institutes of Health (NIH) T32 AI007509-20.

AUTHOR CONTRIBUTIONS

F.M. and J.R.E. designed and performed experiments, analyzed data, and wrote the manuscript. V.V. analyzed data and provided critical input. Y.S. performed experiments. T.B. analyzed data. A.J.T. and J.M. provided critical input. E.W.N., R.G., and M.P. designed the study, analyzed data, and co-wrote the manuscript.

DECLARATION OF INTERESTS

A.J.T. and J.M. are employees of BD Biosciences (manuscript approval by BD Biosciences was not required, and BD Biosciences had no influence regarding data analysis, data interpretation, and discussion). R.G. has received support from Juno Therapeutics and Janssen Pharma; has consulted for Takeda Vaccines, Juno Therapeutics, and Infotech Soft; and has ownership interest in CellSpace Bio. E.W.N. is a cofounder, shareholder, and advisor for Immunoscape Pte. Ltd. and an advisor for Neogene Therapeutics.

Received: July 15, 2019
Revised: October 31, 2019
Accepted: March 18, 2020
Published: April 7, 2020

REFERENCES

Azimifar, S.B., Nagaraj, N., Cox, J., and Mann, M. (2014). Cell-type-resolved quantitative proteomics of murine liver. Cell Metab. *20*, 1076–1087.

Becht, E., McInnes, L., Healy, J., Dutertre, C.A., Kwok, I.W.H., Ng, L.G., Ginhoux, F., and Newell, E.W. (2019). Dimensionality reduction for visualizing single-cell data using UMAP. Nat. Biotechnol. *37*, 38–44.

Butler, A., Hoffman, P., Smibert, P., Papalexi, E., and Satija, R. (2018). Integrating single-cell transcriptomic data across different conditions, technologies, and species. Nat. Biotechnol. *36*, 411–420.

Cao, J., Spielmann, M., Qiu, X., Huang, X., Ibrahim, D.M., Hill, A.J., Zhang, F., Mundlos, S., Christiansen, L., Steemers, F.J., et al. (2019). The single-cell transcriptional landscape of mammalian organogenesis. Nature *566*, 496–502.

Cheng, Y., Wong, M.T., van der Maaten, L., and Newell, E.W. (2016). Categorical Analysis of Human T Cell Heterogeneity with One-Dimensional Soli-Expression by Nonlinear Stochastic Embedding. J. Immunol. *196*, 924–932.

Chiang, M.K., and Melton, D.A. (2003). Single-cell transcript analysis of pancreas development. Dev. Cell *4*, 383–393.

Cooke, A., Schwarzl, T., Huppertz, I., Kramer, G., Mantas, P., Alleaume, A.M., Huber, W., Krijgsveld, J., and Hentze, M.W. (2019). The RNA-Binding Protein YBX3 Controls Amino Acid Levels by Regulating SLC mRNA Abundance. Cell Rep *27*, 3097–3106.e3095.

Cossarizza, A., Chang, H.D., Radbruch, A., Acs, A., Adam, D., Adam-Klages, S., Agace, W.W., Aghaeepour, N., Akdis, M., Allez, M., et al. (2019). Guidelines for the use of flow cytometry and cell sorting in immunological studies (second edition). Eur. J. Immunol. *49*, 1457–1973.

Dunne, P.J., Faint, J.M., Gudgeon, N.H., Fletcher, J.M., Plunkett, F.J., Soares, M.V., Hislop, A.D., Annels, N.E., Rickinson, A.B., Salmon, M., and Akbar, A.N. (2002). Epstein-Barr virus-specific CD8(+) T cells that re-express CD45RA are apoptosis-resistant memory cells that retain replicative potential. Blood *100*, 933–940.

Dutertre, C.A., Becht, E., Irac, S.E., Khalilnezhad, A., Narang, V., Khalilnezhad, S., Ng, P.Y., van den Hoogen, L.L., Leong, J.Y., Lee, B., et al. (2019). Single-Cell Analysis of Human Mononuclear Phagocytes Reveals Subset-Defining Markers and Identifies Circulating Inflammatory Dendritic Cells. Immunity *51*, 573–589.e578.

Edgar, R., Domrachev, M., and Lash, A.E. (2002). Gene Expression Omnibus: NCBI gene expression and hybridization array data repository. Nucleic Acids Res. *30*, 207–210.

Fan, H.C., Fu, G.K., and Fodor, S.P. (2015). Expression profiling. Combinatorial labeling of single cells for gene expression cytometry. Science *347*, 1258367.

Finak, G., McDavid, A., Yajima, M., Deng, J., Gersuk, V., Shalek, A.K., Slichter, C.K., Miller, H.W., McElrath, M.J., Prlic, M., et al. (2015). MAST: a flexible statistical framework for assessing transcriptional changes and characterizing heterogeneity in single-cell RNA sequencing data. Genome Biol. *16*, 278.

Greene, E., Finak, G., and Gottardo, R. (2018). Selective Clustering Annotated using Modes of Projections. arXiv, arXiv:180710328. https://arxiv.org/abs/1807.10328.

Guilliams, M., and Malissen, B. (2016). A Matter of Perspective: Moving from a Pre-omic to a Systems-Biology Vantage of Monocyte-Derived Cell Function and Nomenclature. Immunity *44*, 5–6.

Guilliams, M., Ginhoux, F., Jakubzick, C., Naik, S.H., Onai, N., Schraml, B.U., Segura, E., Tussiwand, R., and Yona, S. (2014). Dendritic cells, monocytes and macrophages: a unified nomenclature based on ontogeny. Nat. Rev. Immunol. *14*, 571–578.

Hamann, D., Baars, P.A., Rep, M.H., Hooibrink, B., Kerkhof-Garde, S.R., Klein, M.R., and van Lier, R.A. (1997). Phenotypic and functional separation of memory and effector human CD8+ T cells. J. Exp. Med. *186*, 1407–1418.

Helft, J., Böttcher, J., Chakravarty, P., Zelenay, S., Huotari, J., Schraml, B.U., Goubau, D., and Reis e Sousa, C. (2015). GM-CSF Mouse Bone Marrow Cultures Comprise a Heterogeneous Population of CD11c(+)MHCII(+) Macrophages and Dendritic Cells. Immunity *42*, 1197–1211.

Jaitin, D.A., Kenigsberg, E., Keren-Shaul, H., Elefant, N., Paul, F., Zaretsky, I., Mildner, A., Cohen, N., Jung, S., Tanay, A., and Amit, I. (2014). Massively parallel single-cell RNA-seq for marker-free decomposition of tissues into cell types. Science *343*, 776–779.

Klein, A.M., Mazutis, L., Akartuna, I., Tallapragada, N., Veres, A., Li, V., Peshkin, L., Weitz, D.A., and Kirschner, M.W. (2015). Droplet barcoding for single-cell transcriptomics applied to embryonic stem cells. Cell *161*, 1187–1201.

Levine, J.H., Simonds, E.F., Bendall, S.C., Davis, K.L., Amir, A.D., Tadmor, M.D., Litvin, O., Fienberg, H.G., Jager, A., Zunder, E.R., et al. (2015). Data-Driven Phenotypic Dissection of AML Reveals Progenitor-like Cells that Correlate with Prognosis. Cell *162*, 184–197.

Linderman, G.C., Rachh, M., Hoskins, J.G., Steinerberger, S., and Kluger, Y. (2019). Fast interpolation-based t-SNE for improved visualization of single-cell RNA-seq data. Nat. Methods *16*, 243–245.

Macosko, E.Z., Basu, A., Satija, R., Nemesh, J., Shekhar, K., Goldman, M., Tirosh, I., Bialas, A.R., Kamitaki, N., Martersteck, E.M., et al. (2015). Highly Parallel Genome-wide Expression Profiling of Individual Cells Using Nanoliter Droplets. Cell *161*, 1202–1214.

Mair, F., and Prlic, M. (2018). OMIP-044: 28-color immunophenotyping of the human dendritic cell compartment. Cytometry A *93*, 402–405.

McInnes, L., Healy, J., and Melville, J. (2018). Umap: Uniform manifold approximation and projection for dimension reduction. arXiv, arXiv:1802.03426v2. https://arxiv.org/abs/1802.03426.

Mori, L., Lepore, M., and De Libero, G. (2016). The Immunology of CD1- and MR1-Restricted T Cells. Annu. Rev. Immunol. *34*, 479–510.

Nagarsheth, N., Wicha, M.S., and Zou, W. (2017). Chemokines in the cancer microenvironment and their relevance in cancer immunotherapy. Nat. Rev. Immunol. *17*, 559–572.

Perfetto, S.P., Ambrozak, D., Nguyen, R., Chattopadhyay, P.K., and Roederer, M. (2012). Quality assurance for polychromatic flow cytometry using a suite of calibration beads. Nat. Protoc. *7*, 2067–2079.

Peterson, V.M., Zhang, K.X., Kumar, N., Wong, J., Li, L., Wilson, D.C., Moore, R., McClanahan, T.K., Sadekova, S., and Klappenbach, J.A. (2017). Multiplexed quantification of proteins and transcripts in single cells. Nat. Biotechnol. *35*, 936–939.

Phillips, J., and Eberwine, J.H. (1996). Antisense RNA Amplification: A Linear Amplification Method for Analyzing the mRNA Population from Single Living Cells. Methods *10*, 283–288.

Sallusto, F., Lenig, D., Förster, R., Lipp, M., and Lanzavecchia, A. (1999). Two subsets of memory T lymphocytes with distinct homing potentials and effector functions. Nature *401*, 708–712.

Sallusto, F., Geginat, J., and Lanzavecchia, A. (2004). Central memory and effector memory T cell subsets: function, generation, and maintenance. Annu. Rev. Immunol. *22*, 745–763.

Schwanhäusser, B., Busse, D., Li, N., Dittmar, G., Schuchhardt, J., Wolf, J., Chen, W., and Selbach, M. (2011). Global quantification of mammalian gene expression control. Nature *473*, 337–342.

See, P., Dutertre, C.-A., Chen, J., Günther, P., McGovern, N., Irac, S.E., Gunawan, M., Beyer, M., Händler, K., Duan, K., et al. (2017). Mapping the human DC lineage through the integration of high-dimensional techniques. Science *356*, eaag3009.

Slichter, C.K., McDavid, A., Miller, H.W., Finak, G., Seymour, B.J., McNevin, J.P., Diaz, G., Czartoski, J.L., McElrath, M.J., Gottardo, R., and Prlic, M. (2016). Distinct activation thresholds of human conventional and innate-like memory T cells. JCI Insight *1*, e86292.

Stoeckius, M., Hafemeister, C., Stephenson, W., Houck-Loomis, B., Chattopadhyay, P.K., Swerdlow, H., Satija, R., and Smibert, P. (2017). Simultaneous epitope and transcriptome measurement in single cells. Nat. Methods *14*, 865–868.

Stoeckius, M., Zheng, S., Houck-Loomis, B., Hao, S., Yeung, B.Z., Mauck, W.M., 3rd, Smibert, P., and Satija, R. (2018). Cell Hashing with barcoded antibodies enables multiplexing and doublet detection for single cell genomics. Genome Biol. *19*, 224.

Stuart, T., and Satija, R. (2019). Integrative single-cell analysis. Nat. Rev. Genet. *20*, 257–272.

van den Broek, T., Borghans, J.A.M., and van Wijk, F. (2018). The full spectrum of human naive T cells. Nat. Rev. Immunol. *18*, 363–373.

Villani, A.-C., Satija, R., Reynolds, G., Sarkizova, S., Shekhar, K., Fletcher, J., Griesbeck, M., Butler, A., Zheng, S., Lazo, S., et al. (2017). Single-cell RNA-seq reveals new types of human blood dendritic cells, monocytes, and progenitors. Science *356*, 1–12.

Zheng, G.X.Y., Terry, J.M., Belgrader, P., Ryvkin, P., Bent, Z.W., Wilson, R., Ziraldo, S.B., Wheeler, T.D., McDermott, G.P., Zhu, J., et al. (2017). Massively parallel digital transcriptional profiling of single cells. Nat. Commun. *8*, 14049.

Zhou, L., Chong, M.M.W., and Littman, D.R. (2009). Plasticity of CD4+ T cell lineage differentiation. Immunity *30*, 646–655.

STAR★METHODS

KEY RESOURCES TABLE

REAGENT or RESOURCE	SOURCE	IDENTIFIER
Antibodies		
CD183 (CXCR3)-BUV395 (clone 1C6)	BD Biosciences	Cat#565223; RRID:AB_2687488
CD3-BUV496 (clone UCHT1)	BD Biosciences	Cat#564809; RRID:AB_2744388
CD25-BUV563 (clone 2A3)	BD Biosciences	Cat#565699; RRID:AB_2744341
HLA-DR-BUV661 (cloneG46-6)	BD Biosciences	Cat#565073; RRID:AB_2722500
ICOS-BUV737 (clone DX29)	BD Biosciences	Cat#564778; RRID:AB_2738947
CD8-BUV805 (clone SK1)	BD Biosciences	Cat#564912; RRID:AB_2744465
CD196 (CCR6)-BV421 (clone 11A9)	BD Biosciences	Cat#562724; RRID:AB_2737747
TCRgd-BV480 (clone DX12)	BD Biosciences	Cat#566084; RRID:AB_2739495
CD14-BV570 (clone M5E2)	BioLegend	Cat#301832; RRID:AB_2563629
PD1-BV605 (clone EH12.1)	BD Biosciences	Cat#563245; RRID:AB_2738091
CD69-BV650 (clone FN50)	BD Biosciences	Cat#563835; RRID:AB_2738442
CD45RA-BV711 (clone UCHL1)	BD Biosciences	Cat#564675; RRID:AB_2738885
CD103-BV750 (clone Ber-ACT8)	BD Biosciences, custom	Cat#624380; no RRID
CD127-BV785 (HIL-7R-M21)	BD Biosciences	Cat#563324; RRID:AB_2738138
Tim3-BB515 (clone 7D3)	BD Biosciences	Cat#565569; RRID:AB_2744368
CD16-BB630 (clone 3G8	BD Biosciences, custom	Cat#624294; no RRID
CD27-BB660 (clone M-T271)	BD Biosciences, custom	Cat#624295; no RRID
CD161-BB700 (clone DX12)	BD Biosciences	Cat#745791; RRID:AB_2743247
CD38-BB790 (clone HIT2)	BD Biosciences, custom	Cat#624296; no RRID
CD194 (CCR4)-PE (clone 1G1)	BD Biosciences	Cat#551120; RRID:AB_394054
CD39-PECF594 (TU66)	BD Biosciences	Cat#563678; RRID:AB_2738367
CD137-PECy5 (clone 4B4-1)	BD Biosciences	Cat#551137; RRID:AB_394067
CD19-PE-Cy5.5 (clone SJ25-C1)	Thermo Fisher	Cat#MHCD1918; RRID:AB_10373840
CD197 (CCR7)-PECy7 (clone 3D12)	BD Biosciences	Cat#557648; RRID:AB_396765
EBV-Tetramer-APC	Fred Hutchinson Immune monitoring core	peptide YVLDHLIVV
CD45RO-AF700 (clone UCHL1)	BioLegend	Cat#304218; RRID:AB_493765
CD4-APCH7 (clone RPA-T4)	BD Biosciences	Cat#560158; RRID:AB_1645478
CD40-BUV395 (clone 5C3)	BD Biosciences	Cat#565202; RRID:AB_2739110
CD56-BUV563 (clone NCAM16.2)	BD Biosciences	Cat#565704; RRID:AB_2744431
CD86-BUV737 (clone FUN-1)	BD Biosciences	Cat#564428; RRID:AB_2738804
CX3CR1-BV421 (clone 2A9-1)	BD Biosciences	Cat#565800; no RRID
CD28-BV480 (clone CD28.2)	BD Biosciences	Cat#566110; RRID:AB_2739512
CD141-BV605 (clone 1A4)	BD Biosciences	Cat#740421; RRID:AB_2740151
Sirpa-BV650 (clone SE5A5)	BD Biosciences	Cat#743565; no RRID
OX40-BV711 (clone ACT-35)	BioLegend	Cat#350029; RRID:AB_2632863
CD11b-BV750 (clone ICRF44)	BD Biosciences, custom	Cat#624380; no RRID
CD123-BV786 (clone 7G3)	BD Biosciences	Cat#564196; RRID:AB_2738662
CD206-BB515 (clone 19.2)	BD Biosciences	Cat#564668; RRID:AB_2738882
CD32-BB700 (clone FLI8.26)	BD Biosciences	Cat#742216; no RRID
Lag3-PE (clone T47-530)	BD Biosciences	Cat#565617; no RRID
CD163-PECF594 (clone GHI/61)	BD Biosciences	Cat#562670; RRID:AB_2737711
CD80-PECy5 (clone L307.4)	BD Biosciences	Cat#559370; RRID:AB_397239
CD4-PECy7 (clone SK3)	BD Biosciences	Cat#557852; RRID:AB_396897

(*Continued on next page*)

Continued

REAGENT or RESOURCE	SOURCE	IDENTIFIER
CD1c-AF647 (clone F10/21A3)	BD Biosciences	Cat#565048; RRID:AB_2744318
CD11c-AF700 (clone B-ly6)	BD Biosciences	Cat#561352; RRID:AB_10612006
HLA-DR-APCH7 (clone L243)	BD Biosciences	Cat#561358; RRID:AB_10611876
UV Fixable Live-Dead	Thermo Fisher	Cat#L34961; no RRID
Human TruStain FcX (Fc-Block)	BioLegend	Cat#422302; no RRID
Cytofix/CytoPerm	BD Biosciences	Cat#554722; no RRID
CD3-Ab-O (clone SK7)	BD Biosciences	AHS0033; no RRID
CD4-Ab-O (clone SK3)	BD Biosciences	AHS0032; no RRID
CD8-Ab-O (clone RPA-T8)	BD Biosciences	AHS0027; no RRID
CD19-Ab-O (clone SJ25C1)	BD Biosciences	AHS0030; no RRID
CD14-Ab-O (clone MPHIP9)	BD Biosciences	AHS0037; no RRID
CD16-Ab-O (clone 3G8)	BD Biosciences	AHS0053; no RRID
CD56-Ab-O (clone NCAM16.2)	BD Biosciences	AHS0019; no RRID
CD11b-Ab-O (clone M1/70)	BD Biosciences	AHS0005; no RRID
CD25-Ab-O (clone 2A3)	BD Biosciences	AHS0026; no RRID
HLA-DR-Ab-O (cloneG46-6)	BD Biosciences	AHS0035; no RRID
CD45RA-Ab-O (clone HI100)	BD Biosciences	AHS0009; no RRID
CD127-Ab-O (clone HIL-7R-M21)	BD Biosciences	AHS0028; no RRID
CD38-Ab-O (clone HIT2)	BD Biosciences	AHS0022; no RRID
CD197-Ab-O (clone 3D12)	BD Biosciences	AHS0007; no RRID
CD279-Ab-O (clone EH12.1)	BD Biosciences	AHS0014; no RRID
CD28-Ab-O (clone CD28.2)	BD Biosciences	AHS0024; no RRID
CD27-Ab-O (clone M-T271)	BD Biosciences	AHS0025; no RRID
CD69-Ab-O (clone FN50)	BD Biosciences	AHS0010; no RRID
CD123-Ab-O (clone 7G3)	BD Biosciences	AHS0020; no RRID
CD45RO-Ab-O (clone UCHL1)	BD Biosciences	AHS0036; no RRID
CD11c-Ab-O (clone B-Ly6)	BD Biosciences	AHS0056; no RRID
CD86-Ab-O (clone FUN-1)	BD Biosciences	AHS0057; no RRID
CD183-Ab-O (clone 1C6)	BD Biosciences	AHS0031; no RRID
CD196-Ab-O (clone 11A9)	BD Biosciences	AHS0034; no RRID
CD80-Ab-O (clone L307.4)	BD Biosciences	AHS0046; no RRID
CD278-Ab-O (clone DX29)	BD Biosciences	AHS0012; no RRID
CD194-Ab-O (clone 1G1)	BD Biosciences	AHS0038; no RRID
CD40-Ab-O (clone 5C2)	BD Biosciences	AHS0117; no RRID
CD137-Ab-O (clone 4B4-1)	BD Biosciences	AHS0003; no RRID
TCRgd-Ab-O (clone B1)	BD Biosciences	AHS0015; no RRID
CD163-Ab-O (clone GH1/61)	BD Biosciences	AHS0062; no RRID
CD134-Ab-O (clone ACT35)	BD Biosciences	AHS0013; no RRID
Tim3-Ab-O (clone 7D3)	BD Biosciences	AHS0016; no RRID
CD103-Ab-O (clone Ber-ACT8)	BD Biosciences	AHS0016; no RRID
CD206-Ab-O (clone 19.2)	BD Biosciences	AHS0072; no RRID
CD32-Ab-O (clone FLI8.26)	BD Biosciences	AHS0073; no RRID
CD161-Ab-O (clone DX12)	BD Biosciences	AHS0002; no RRID
CD39-Ab-O (clone TU66)	BD Biosciences	AHS0006; no RRID
CD141-Ab-O (clone 1A4)	BD Biosciences	AHS0083; no RRID
Lag3-Ab-O (clone T47-530)	BD Biosciences	AHS0018; no RRID
CD1c-Ab-O (clone F10/21A3)	BD Biosciences	AHS0088; no RRID

(*Continued on next page*)

Continued

REAGENT or RESOURCE	SOURCE	IDENTIFIER
Biological Samples		
Cryopreserved peripheral blood mononuclear cells	HVTN, Fred Hutch	NA
Critical Commercial Assays		
Rhapsody AbSeq reagent pack (4 reactions)	BD Biosciences	Cat#633771
Rhapsody Human T cell expression panel	BD Biosciences	Cat#633751
Rhapsody Human Immune Response panel	BD Biosciences	Cat#633750
Rhapsody custom gene panel (see Table S1)	BD Biosciences	Cat#633743
Human Single cell multiplexing kit	BD Biosciences	Cat#633781
Deposited Data		
Flow Cytometry Data	http://www.flowrepository.org	FR-FCM-Z266
sc-RNaseq/AbSeq Data	https://www.ncbi.nlm.nih.gov/geo/	GEO: GSE135325
Software and Algorithms		
R Studio and R environment	The R project for Statistical Computing	https://rstudio.com/ and https://cran.r-project.org/
Seurat v2.3 and v3.0	Satija Lab, NYU, New York Genome Center	https://github.com/satijalab/seurat
Seven Bridges (standard pre-processing of Rhapsody raw sequencing data, i.e., FASTQ files)	BD Biosciences	https://www.sevenbridges.com
CellRanger (standard pre-processing of WTA raw sequencing data, i.e., FASTQ files)	10x genomics	https://www.10xgenomics.com/
FlowJo 10.5.x (analysis and visualization of flow cytometry and AbSeq data)	BD Biosciences	https://www.flowjo.com
Prism (plotting)	GraphPad	N/A
Illustrator (figure generation)	Adobe	N/A
Seurat workflow for all WTA and targeted trancriptomic single cell molecule count tables	Prlic Lab, FHCRC, Seattle	https://github.com/MairFlo/
One-SENSE (visualization of data protein-transcript)	Newell Lab, FHCRC, Seattle	https://github.com/MairFlo/
Other		
FACSymphony flow cytometer	BD Biosciences	N/A
Rhapsody Express instrument	BD Biosciences	N/A

LEAD CONTACT AND MATERIALS AVAILABILITY

Further information and requests for resources and reagents should be directed to and will be fulfilled by the Lead Contact, Martin Prlic, at mprlic@fredhutch.org. This study did not generate new unique reagents.

EXPERIMENTAL MODEL AND SUBJECT DETAILS

Primary Cells

Peripheral blood mononuclear cells (PBMCs) were obtained as cryopreserved samples from healthy controls (Seattle Area Control Cohort) via the HIV Vaccine Trial network (HVTN). Researchers authored on this manuscript did not have access to any patient information. Vials with cryopreserved cells were thawed at 37°C until a tiny ice crystal was left in the tube, and then carefully diluted in 1mL of pre-warmed complete RPMI (RPMI (GIBCO, #18875119) with 10% FBS (Nucleus Biologics, #AU FBS-500ml L1 HI) and 1% Penicillin-Streptomycin (GIBCO, #15140122) and transferred to a new tube. An additional 13 mL of pre-warmed complete RPMI were added drop by drop, followed by centrifugation for 5 minutes at 400 g and resuspension in 1 mL of complete RPMI.

METHOD DETAILS

T cell stimulation assay

Freshly thawed PBMCs (Seattle Area Control Cohort) were depleted of myeloid cells, B cells and IL3Rα-expressing cells using magnetic-activated cell sorting (MACS) with antibodies targeting CD33, CD20 and CD123 and anti-mouse IgG microbeads (Miltenyi #130-048-402). The purified cell fraction was washed and stimulated in RPMI with 10% FBS with PMA (10 ng/mL) and Ionomycin (1 μg/mL) for 60 minutes at 37°C. Unstimulated cells were incubated without PMA/Ionomycin for 60 minutes at 37°C. After that, cells were stained with AbSeq antibody-oligo-conjugates targeting CD3, TCRαβ, CD4, CD8, PD1, CD137, CD103, CD69, CD39, CCR7, CD45RA, CD45RO and Tim3, strictly following the manufacturers protocol (BD Biosciences) and subjected to the targeted transcriptomic workflow described below, using the BD Rhapsody T Cell Expression Panel Hs (BD Biosciences, #633751).

Flow Cytometry and Cell sorting

For flow cytometric analysis good practices were followed as outlined in the guidelines for use of flow cytometry (Cossarizza et al., 2019). Following thawing, PBMCs were incubated with Fc-blocking reagent (BioLegend Trustain FcX, #422302) and fixable UV Blue Live/Dead reagent (ThermoFisher, #L34961) in PBS (GIBCO, #14190250) for 15 minutes at room temperature. If required, cells were stained with an EBV-Tetramer reagent (peptide YVLDHLIVV; Fred Hutch Immune Monitoring Core) diluted in FACS buffer (PBS with 2% FBS, Nucleus Biologics, #AU FBS-500ml L1 HI) for 30 minutes at room temperature, followed by two washes. After this, cells were incubated for 20 minutes at room temperature with 100 μL total volume of antibody master mix freshly prepared in Brilliant staining buffer (BD Bioscience, #563794), followed by two washes. All antibodies were titrated and used at optimal dilution, and staining procedures were performed in 96-well round-bottom plates. A detailed list of panels used, including fluorochromes and final dilutions of all antibodies are listed in Table S4. Stained cells were fixed with 4% PFA for 20 minutes at room temperature, washed, resuspended in FACS buffer and stored at 4°C in the dark until acquisition.

All samples were acquired using a FACSymphony A5 (BD Biosciences), equipped with 30 detectors and 355nm, 405nm, 488nm, 532nm and 628nm lasers and FACSDiva (BD Biosciences). Detector gains were optimized using a modified voltage titration approach (Perfetto et al., 2012) and standardized from day to day using 6-peak Ultra Rainbow Beads (Spherotec, # URCP-38-2K). Single-stained controls were prepared with every experiment using antibody capture beads diluted in FACS buffer (BD Biosciences anti-mouse, #552843 and anti-rat, #552844), or cells for Live/Dead reagent. After acquisition, data was exported in FCS 3.1 format and analyzed using FlowJo (version 10.5.x, BD Biosciences). Doublets were excluded by FSC-A versus FSC-H gating. For some of the plots, the number of acquired cells was down-sampled using the appropriate FlowJo plugin to match the number of cells analyzed in the AbSeq workflow.

All cell sorting was performed on a FACSAria III (BD Biosciences), equipped with 20 detectors and 405nm, 488nm, 532nm and 628nm lasers. For all sorts, an 85 μm nozzle operated at 45 psi sheath pressure was used. Cells were sorted into chilled Eppendorf tubes containing 500 μL of complete RPMI, washed once in PBS and immediately used for subsequent processing.

Targeted Transcriptome and protein single-cell library preparation and Sequencing

For all targeted transcriptomics experiments, we utilized the BD Rhapsody Express system, which is based on Fan et al. (2015) with some adaptations prior to the commercial release by BD Biosciences. $CD45^+$ live PBMCs and EBV-tetramer$^+$ $CD8^+$ T cells were sequentially labeled using Single Cell Labeling with the BD Single-Cell Multiplexing Kit (BD Biosciences, #633781) and BD AbSeq Ab-Oligos reagents strictly following the manufacturers protocol (BD Biosciences). Briefly, cells from each donor or subtype of cells (after sorting) were labeled with sample tags (Stoeckius et al., 2018). Each sample was washed twice with FACS buffer before pooling all samples together. Pooled samples were washed one more time and then stained in a total volume of 200 μL of FACS buffer with AbSeq Ab-Oligos (BD Biosciences) diluted as listed in Table S4. The pooled sample was then washed twice, counted and resuspended in cold BD Sample Buffer (BD Biosciences) to achieve approximately 20,000 cells in 620 μL. Single cells from the pooled sample were isolated using Single Cell Capture and cDNA Synthesis with the BD Rhapsody Express Single-Cell Analysis System following the manufacturers protocol (BD Biosciences). After priming the nanowell cartridges, the pooled sample was loaded onto two BD Rhapsody cartridges and incubated at room temperature. Cell Capture Beads (BD Biosciences) were prepared and then loaded onto the cartridge and incubated prior to shaking at 1,000 rpm at room temperature for 15 s on a ThermoMixer C (Eppendorf). According to the manufacturers protocol, cartridges were washed, cells were lysed, and Cell Capture Beads were retrieved and washed prior to performing reverse transcription and treatment with Exonuclease I. cDNA Libraries were prepared using mRNA Targeted, Sample Tag, and BD AbSeq Library Preparation with the BD Rhapsody Targeted mRNA and AbSeq Amplification and BD Single-Cell Multiplexing Kits and protocol (BD Biosciences). In brief, cDNA underwent targeted amplification using the Human Immune Response Panel primers and a custom supplemental panel (all 492 targets are listed in Table S1) via PCR (10 cycles). PCR products were purified, and mRNA PCR products were separated from sample tag and AbSeq products with double-sided size selection using SPRIselect magnetic beads (Beckman Coulter, #B23318). mRNA and Sample Tag products were further amplified using PCR (10 cycles). PCR products were then purified using SPRIselect magnetic beads. Quality and quantity of PCR products were determined by using an Agilent 2200 TapeStation with High Sensitivity D5000 ScreenTape (Agilent) in the Fred Hutch Genomics Shared Resource laboratory. Targeted mRNA product was diluted to 2.5 ng/μL and sample tag and AbSeq PCR products were diluted to 1 ng/μL to prepare final libraries. Final libraries were indexed using PCR (6 cycles). Index PCR products were purified using SPRIselect magnetic beads. Qual-

ity of final libraries was assessed by using Agilent 2200 TapeStation with High Sensitivity D5000 ScreenTape and quantified using a Qubit Fluorometer using the Qubit dsDNA HS Kit (ThermoFisher, #Q32854). Final libraries were diluted to 2 nM and multiplexed for paired-end (150bp) sequencing on a HiSeq 2500 sequencer (Illumina). The final mean read depths for the three experiments that we sequenced were as follows: Experiment 1 (used in Figure S3): 18,445 reads/cell for AbSeq library (saturation 88.4%), 9,137 reads/cell for mRNA library (saturation 86.7%). Experiment 2 (main figures): 4,330 reads/cell for AbSeq library (saturation 53.3%), 2,165 reads/cell for mRNA library (saturation 89.9%). Experiment 3 (used for Figure 4; T cell stimulation assay): 19,236 reads/cell for the AbSeq library (saturation 94.7%), 14,042 reads/cell for the mRNA library (saturation 94.9%).

Whole Transcriptome single-cell library preparation and sequencing

cDNA libraries of $CD45^+$ Live PBMCs were generated using the Chromium Single Cell 3′ Reagent Kits v2 (10x Genomics) protocol targeting 5,000 cells in two separate wells. Briefly, single cells were isolated into oil emulsion droplets with barcoded gel beads and reverse transcriptase mix. cDNA was generated within these droplets, then the droplets were dissociated. cDNA was purified using DynaBeads MyOne Silane magnetic beads (ThermoFisher, #370002D). cDNA amplification was performed by PCR (10 cycles) using reagents within the Chromium Single Cell 3′ Reagent Kit v2 (10x Genomics). Amplified cDNA was purified using SPRIselect magnetic beads (Beckman Coulter). cDNA was enzymatically fragmented and size selected prior to library construction. Libraries were constructed by performing end repair, A-tailing, adaptor ligation, and PCR (12 cycles). Quality of the libraries was assessed by using Agilent 2200 TapeStation with High Sensitivity D5000 ScreenTape (Agilent). Quantity of libraries was assessed by performing digital droplet PCR (ddPCR) with Library Quantification Kit for Illumina TruSeq (BioRad, #1863040). Libraries were diluted to 2 nM and paired-end sequencing was performed on a HiSeq 2500 sequencer (Illumina). The final read depths for the two technical replicates that we sequenced were 77,049 reads/cell and 86,246 reads/cell, respectively.

QUANTIFICATION AND STATISTICAL ANALYSIS

Cell Ranger processing for WTA data

Raw base call (BCL) files were demultiplexed to generate Fastq files using the cellranger mkfastq pipeline within Cell Ranger 2.1.1 (10x Genomics). Targeted transcriptome Fastqs were further analyzed via Seven Bridges (BD Biosciences). Whole transcriptome Fastq files were processed using the standard cellranger pipeline (10x genomics) within Cell Ranger 2.1.1. Briefly, cellranger count performs alignment, filtering, barcode counting, and UMI counting. The cellranger count output was fed into the cellranger aggr pipeline to normalize sequencing depth between samples. The final output of cellranger (molecule per cell matrix) was then analyzed in R using the package Seurat (version 2.3 and 3.0) as described below.

Seven Bridges processing for targeted transcriptomics data

Targeted transcriptomics Fastq files were processed via the standard Rhapsody analysis pipeline (BD Biosciences) on Seven Bridges (https://www.sevenbridges.com) per the manufacturer's recommendations. First, R1 and R2 reads are filtered for high-quality reads, dropping reads that are too short (less than 64 bases for R2) or have a base quality score of less than 20. Then, R1 reads are annotated to identify cell label sequences and unique molecular identifiers (UMIs), and R2 reads are mapped to the respective reference sequences using Bowtie2. Finally, all valid R1 and R2 reads are combined and annotated to the respective molecules. For all of our analysis, we utilized recursive substation error correction (RSEC) as well as distribution-based error correction (DBEC), which are manufacturer-developed algorithms correcting for PCR and sequencing errors. For determining putative cells (which will contain many more reads than noise cell labels), a filtering algorithm takes the number of DBEC-corrected reads into account, calculating the minimum second derivative along the cumulative reads as the cut-off point. Final expression matrices contain DBEC-adjusted molecule counts in a CSV format. For sample tag assignment, a cell is called as a singlet if the minimum read count for a given sample tag is reached, and more than 75% of the sample tag reads are derived from a single sample tag antibody. In turn, if the count for two more sample tag antibodies exceeds the minimum thresholds, these cells are labeled as multiplets, and if a cell does not reach criteria for either a multiplet or singlet, it is labeled as undetermined. Both multiplets and undetermined cells were excluded from analysis as described below.

For further analysis, molecule count tables were read into the R package Seurat (version 2.3 and 3.0) using customized scripts and analyzed as described below.

Seurat workflow for targeted and WTA data

The R package Seurat (Butler et al., 2018) was utilized for all downstream analysis. For whole transcriptome data, based on commonly used cutoffs suggested by Butler et al., only cells that had at least 200 genes (with $\leq$ 20% being mitochondrial genes) were included in analysis (removing 182 out of a total of 5,416 cells). A natural log normalization using a scale factor of 10,000 was performed across the library for each cell. UMIs and mitochondrial genes (only for WTA data) were linearly scaled to remove these variables as unwanted sources of variation. For the targeted data, singlets were identified during pre-processing as described above. In our case, out of 29,033 total cells, 1,532 were called as doublets (and 243 events as undetermined), with 27,258 cells re-

maining (donor 1: 8,984 cells, donor 2: 8,956 cells, donor 3: 9,318 cells). For WTA data, doublets and low-quality cells were identified by their outlier UMI and gene counts (more than 15,000 UMIs and more than 3,000 genes), and their high percentage of mitochondrial genes (more than 20%), removing 640 cells.

For WTA, dimensionality reduction using UMAP and clustering was performed on a subset of variable genes. For targeted transcriptomics, no gene per cell cutoffs were imposed and the data was normalized with the same method (natural log normalization using a scale factor of 10,000). To make sure that the same normalization methods could be used, we compared the library size factor distribution (using the package scater) between the WTA and targeted transcriptomic data, which showed similar distributions irrespective of whether using all genes or only the targeted gene set (however, further comparative experiments might be required to ensure that the standard log normalization implemented in Seurat is an effective normalization method for different targeted transcriptomic experimental setups). When scaling data, UMI was the only regressed variable. Dimensionality reduction using UMAP and clustering was based on either all genes or all proteins. For differential gene expression analysis we utilized the Seurat implementation of MAST (model-based analysis of single-cell transcriptomes) with the number of UMIs included as a covariate (proxy for cellular detection rate (CDR)) in the model (Finak et al., 2015).

For generation of some FCS files the antibody molecule count tables were converted using the R packages premessa and flowCore. FCS-files with antibody molecule count signals were analyzed in FlowJo 10.5.x (BD Biosciences) using either an arcsinh transformation or biexponential transformation.

Data processing for One-SENSE and generation of FCS files

CSV files of raw counts were converted to FCS files using a script adapted from https://gist.github.com/yannabraham/c1f9de9b23fb94105ca5. Raw counts were normalized based on total counts per cell, then scaled to a value of 10,000 based on the Seurat normalization algorithm. A natural log transformation was applied to gene expression data, while protein expression data was randomized by adding a random uniform distribution from 0 to 1, followed by transformation with the function arcsinh(x/5). Dimensionality reduction using UMAP was performed separately on all genes and proteins to reduce them to one dimension before plotting. Cells were also split into 500 bins of equivalent width based on one-dimensional UMAP data, then used to generate heatmaps colored by median marker intensity per bin (low expression: blue, mid expression: green, high expression: red). Heatmaps corresponding to empty areas of the One-SENSE plot were colored in black. An example script for One-SENSE processing can be found at https://github.com/MairFlo/Targeted_transcriptomics.

DATA AND CODE AVAILABILITY

The sequencing data discussed in this publication have been deposited in the NCBI's Gene Expression Omnibus (Edgar et al., 2002) and are accessible through GEO series accession number GEO: GSE135325. (https://www.ncbi.nlm.nih.gov/geo/query/acc.cgi?acc=GSE135325). The accession number for all flow cytometry data reported in this paper is FlowRepository: FR-FCM-Z266 (http://flowrepository.org/id/FR-FCM-Z266). All scripts used for data processing and plot generation are available at https://github.com/MairFlo/Targeted_transcriptomics.

Cell Reports, Volume *31*

Supplemental Information

A Targeted Multi-omic Analysis Approach Measures Protein Expression and Low-Abundance Transcripts on the Single-Cell Level

Florian Mair, Jami R. Erickson, Valentin Voillet, Yannick Simoni, Timothy Bi, Aaron J. Tyznik, Jody Martin, Raphael Gottardo, Evan W. Newell, and Martin Prlic

Suppl. Figure 1

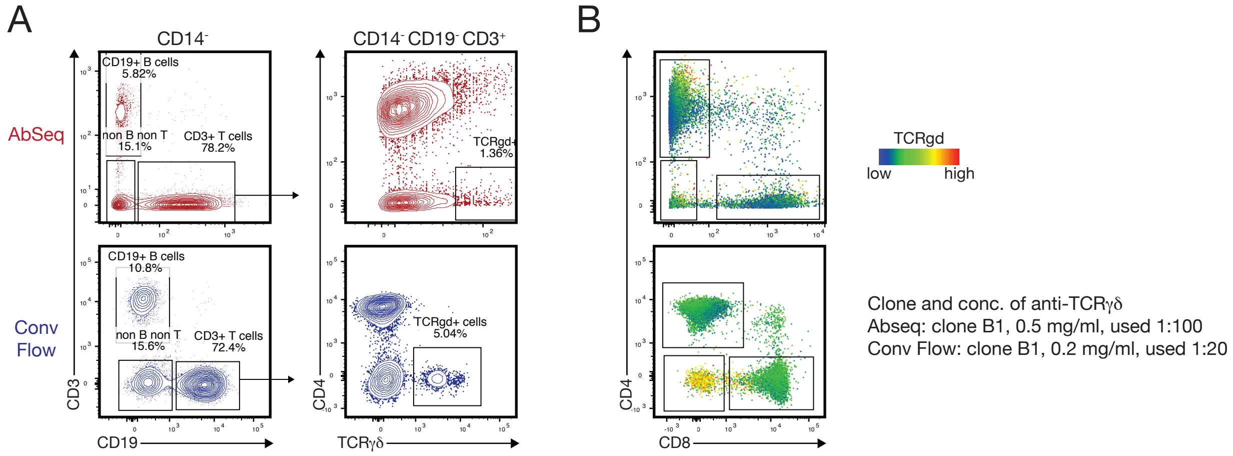

Supplementary figure 1 (related to figure 1): Example for a poorly performing reagent
(A) Manual gating of main immune subsets using the combined AbSeq data set (upper panel, red) and concatenated and downsampled events from the flow cytometry data set (lower panel, blue), highlighting the population of γδ T cells.
(B) Heatmap overlay of the TCRγδ signal on a CD4 vs CD8 plot for the AbSeq data set (upper panel) and flow cytometry data set (lower panel). Antibody stock concentrations and dilutions are listed.

Suppl. Figure 2

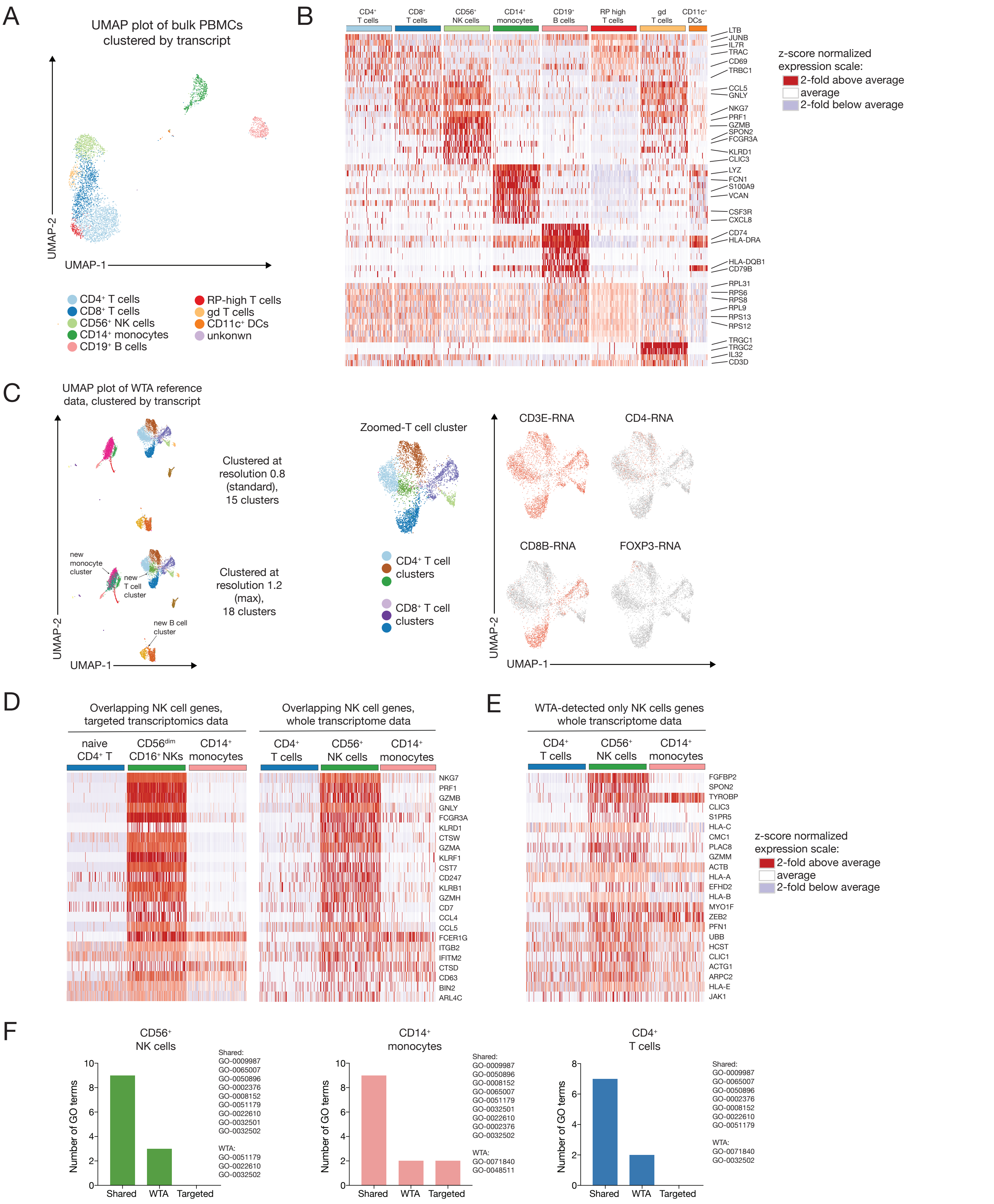

Supplementary figure 2 (related to figure 2): Comparison of targeted transcriptomic and WTA data
(A) Graph-based clustering of WTA data obtained from the same donor as in main Figure 2.
(B) The top 10-differentially expressed genes for each cluster were identified using the Seurat implementation of MAST and visualized on a heatmap after z-score normalization. Cluster names are shown in the same color scheme as in (A).
(C) Graph-based clustering of WTA data from a public whole transcriptome reference data set at two different clustering resolution (left) and the zoomed T cell-population with an heatmap overlay showing CD3E, CD4, CD8B and FOXP expression.
(D) Expression pattern of all genes that were detected as differentially expressed both in WTA and targeted transcriptomic data for the NK cell cluster relative to naive CD4+ T cells and CD14+ monocytes.
(E) Expression pattern of genes that were only detected in the NK cell cluster of the WTA data.
(F) Number of gene ontology (GO)-terms that were shared between both data sets, or only detected in either the WTA or targeted data. GO-Term accession numbers are listed next to the bar graphs.

Suppl. Figure 3

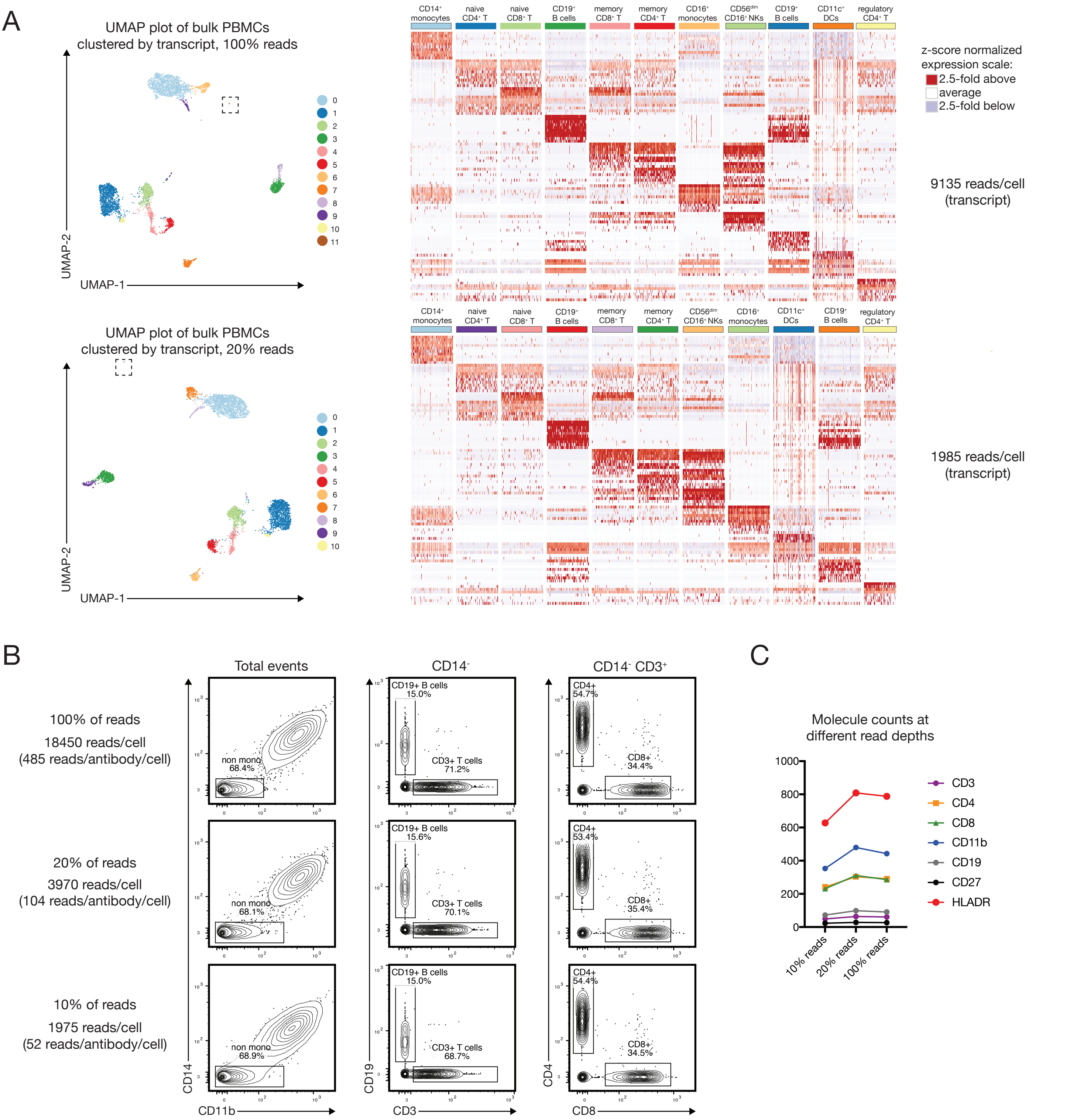

Supplementary figure 3 (related to figure 2): Effect of different read depths on the resolution of targeted transcriptomic and AbSeq data

(A) 5,400 cells from a different donor were sequenced at a total depth of approximately 30,000 reads/cell. Upper panel depicts UMAP plot after graph-based clustering (using transcript) and a heatmap of the top differentially expressed genes at full read depth, lower panel using only 20% of the reads. Read depth per cell for the transcript library is indicated on the right. Squared box on the UMAP plot indicates one cluster that is separated as cluster 11 at full read depth, but pooled with cluster 8 at lower read-depth.

(B) Representative protein signals at the indicated read depths.

(C) Positive cells for the indicated markers (only bimodal populations were selected) were gated, median molecule counts calculated and plotted across the different read depths.

Supplementary Table 1
List of genes for targeted transcriptomics assay, related to main figure 2

Human Immune Response Panel							
1	ADA	100	CEACAM8	200	IGHD_membrane	300	NCAM1
2	ADGRE1	101	CHI3L1	201	IGHE_secreted	301	NCR3
3	ADGRG3	102	CHI3L2	202	IGHG1_membrane	302	NINJ2
4	AIM2	103	CLC	203	IGHG1_secreted	303	NKG7
5	ALAS2	104	CLEC10A	204	IGHG2_secreted	304	NRP1
6	ANXA5	105	CLEC4D	205	IGHG3_secreted	305	NT5E
7	AOC3	106	CLEC4E	206	IGHG4_secreted	306	PASK
8	APOBEC3G	107	CMKLR1	207	IGHM_membrane	307	PAX5
9	APOE	108	CMTM2	208	IGHM_secreted	308	PCNA
10	AQP9	109	CNOT2	209	IGKC	309	PDCD1
11	ARG1	110	CNTNAP3	210	IGLC3	310	PDIA4
12	ARL4C	111	CPA3	211	IKZF1	311	PDIA6
13	ATF6B	112	CR2	212	IKZF2	312	PI3
14	AURKB	113	CSF2	213	IL12A	313	PIK3AP1
15	AZU1	114	CSF3	214	IL12RB1	314	PIK3IP1
16	B3GAT1	115	CST7	215	IL12RB2	315	PMCH
17	BACH2	116	CTLA4	216	IL13	316	POU2AF1
18	BAX	117	CTSD	217	IL15	317	PRDM1
19	BCL11B	118	CTSG	218	IL15RA	318	PRF1
20	BCL2	119	CTSW	219	IL17A	319	PSEN1
21	BCL2A1	120	CX3CR1	220	IL17F	320	PTGDR2
22	BCL6	121	CXCL1	221	IL18	321	PTPRC
23	BIN2	122	CXCL10	222	IL18R1	322	PTTG2
24	BIRC3	123	CXCL11	223	IL18RAP	323	QPCT
25	BLK	124	CXCL13	224	IL1B	324	RGS1
26	BLNK	125	CXCL16	225	IL1R2	325	RNASE2
27	BPI	126	CXCL2	226	IL1RL1	326	RNASE6
28	BTG1	127	CXCL3	227	IL1RN	327	RORA
29	BTLA	128	CXCL5	228	IL2	328	RORC
30	C10orf54	129	CXCL8	229	IL21	329	RPN2
31	C1QA	130	CXCL9	230	IL22	330	RUNX3
32	C1QB	131	CXCR1	231	IL23R	331	S100A10
33	CASP5	132	CXCR2	232	IL25	332	S100A12
34	CBLB	133	CXCR3	233	IL2RA	333	S100A9
35	CCL1	134	CXCR4	234	IL2RB	334	SELL
36	CCL13	135	CXCR5	235	IL3	335	SELPLG
37	CCL17	136	CXCR6	236	IL31	336	SLC25A37
38	CCL19	137	DEFA3	237	IL32	337	SLC7A7
39	CCL2	138	DEFA4	238	IL33	338	SNCA
40	CCL20	139	DOCK8	239	IL3RA	339	SPP1
41	CCL22	140	DPP4	240	IL4	340	STAT1
42	CCL3	141	DUSP1	241	IL4R	341	STAT3
43	CCL4	142	DUSP2	242	IL5	342	STAT4
44	CCL5	143	DUSP4	243	IL6	343	STAT5A
45	CCND2	144	EBF1	244	IL7R	344	STAT6
46	CCR1	145	EGR1	245	IL9	345	TARP_refseq
47	CCR10	146	EGR3	246	IRF4	346	TBX21
48	CCR2	147	ELANE	247	IRF8	347	TCF4
49	CCR3	148	ENTPD1	248	ITGA4	348	TCF7
50	CCR4	149	EOMES	249	ITGAE	349	TCL1A
51	CCR5	150	EPX	250	ITGAM	350	TGFB1
52	CCR7	151	F13A1	251	ITGAX	351	TGFB3
53	CCR8	152	F5	252	ITGB2	352	TGFBI
54	CCR9	153	FAM129C	253	JCHAIN	353	THBD
55	CD14	154	FAM65B	254	JUN	354	THBS1
56	CD160	155	FAS	255	JUNB	355	TIAF1
57	CD163	156	FASLG	256	KCNE3	356	TIGIT
58	CD1A	157	FCER1A	257	KDELR1	357	TLR2
59	CD1B	158	FCER1G	258	KIAA0101	358	TLR7
60	CD1C	159	FCER2	259	KIR2DL1	359	TLR8
61	CD2	160	FCGR3A	260	KIT	360	TLR9
62	CD200	161	FCN1	261	KLRB1	361	TMEM97
63	CD209	162	FLT3	262	KLRC1	362	TNF
64	CD22	163	FN1	263	KLRC3	363	TNFRSF13C
65	CD24	164	FOSB	264	KLRC4	364	TNFRSF17
66	CD244	165	FOSL1	265	KLRF1	365	TNFRSF25
67	CD247	166	FOXO1	266	KLRG1	366	TNFRSF4
68	CD27	167	FOXP1	267	KLRK1	367	TNFRSF8
69	CD274	168	FOXP3	268	LAG3	368	TNFRSF9
70	CD28	169	FTH1	269	LAIR2	369	TNFSF10
71	CD300A	170	FUT4	270	LAMP1	370	TNFSF13
72	CD33	171	FYB	271	LAMP3	371	TNFSF13B
73	CD34	172	FYN	272	LAP3	372	TNFSF14
74	CD36	173	GAB2	273	LAT	373	TNFSF8
75	CD37	174	GAPDH	274	LAT2	374	TOP2A
76	CD38	175	GIMAP2	275	LCK	375	TPSAB1
77	CD3D	176	GIMAP5	276	LEF1	376	TRAC
78	CD3E	177	GNAI2	277	LGALS1	377	TRAT1
79	CD3G	178	GNLY	278	LGALS3	378	TRBC2
80	CD4	179	GZMA	279	LGALS9	379	TRDC
81	CD40	180	GZMB	280	LIF	380	TREM1
82	CD44	181	GZMH	281	LILRB4	381	TRIB2
83	CD48	182	GZMK	282	LIPA	382	TSPAN32
84	CD5	183	HAVCR2	283	LRRC32	383	TXK
85	CD52	184	HLA-A	284	LTA	384	TYMS
86	CD6	185	HLA-DMA	285	LTB	385	UBE2C
87	CD63	186	HLA-DPA1	286	LY86	386	VEGFA
88	CD69	187	HLA-DQB1	287	LYN	387	VMO1
89	CD7	188	HLA-DRA	288	MCM2	388	VNN2
90	CD70	189	HMMR	289	MCM4	389	VPREB3
91	CD72	190	ICAM1	290	MGST1	390	VPS28
92	CD74	191	ICOS	291	MITF	391	VSIG4
93	CD79A	192	IER3	292	MKI67	392	XBP1
94	CD79B	193	IFITM2	293	MME	393	YBX3
95	CD80	194	IFITM3	294	MMP12	394	ZAP70
96	CD86	195	IFNA1	295	MMP9	395	ZBED2
97	CD8A	196	IFNG	296	MS4A1	396	ZBTB16
98	CD8B	197	IFNGR1	297	MYC	397	ZNF683
99	CD9	198	IGBP1	298	MZB1		
		199	IGHA1_sec	299	NAMPT		

Custom Gene Panel	
1	ADAM15
2	ADAM17
3	ADAM28
4	AHR
5	AIF1
6	APOC1
7	AXL
8	BATF
9	BIRC5
10	CA10
11	CAPG
12	CCL3L3
13	CCL4L2
14	CCR6
15	CD207
16	CD273
17	CD40LG
18	CD68
19	CD83
20	CD96
21	CDH1
22	CLU
23	CRTAM
24	CSF1
25	CTSK
26	CTSS
27	NSG1
28	DUSP6
29	FBLN2
30	FBN2
31	FCGR3B
32	FOS
33	FOXO3
34	GATA3
35	GZMM
36	HIF1A
37	HLA-DPB1
38	HLA-DQA1
39	HOPX
40	ICOSLG
41	ID3
42	IFITM1
43	IL10
44	IL22RA2
45	IL23A
46	IL6R
47	IRF7
48	ITGA1
49	ITGA5
50	KCNK5
51	KLF2
52	KLF3
53	KLF6
54	KLRC2
55	KLRD1
56	LTC4S
57	LYZ
58	MFAP2
59	MMP1
60	MMP2
61	MNDA
62	MX1
63	MX2
64	MXRA8
65	NLRP3
66	NPDC1
67	PTGDS
68	PTPN6
69	PXDN
70	RAP1GAP2
71	RhoA
72	S100A8
73	S1PR1
74	SBK1
75	SERPINA1
76	SOCS3
77	SOX13
78	STK38
79	TCF21
80	TIMP1
81	TIMP2
82	TIMP3
83	TIMP4
84	TMEM123
85	TNFRSF18
86	TNFRSF1B
87	TOX
88	TSPAN18
89	TTC16
90	TTYH2
91	VCAN
92	XCL1
93	XCL2
94	ZFP36
95	ZNF219

Supplementary Table 2, related to main figure 2
Relative detection ratio of genes in the targeted transcriptomic assay relative to WTA for one donor
Color coding and order of genes is the same as in main figure 2D

	Raw Counts			Normalized to cell#			
**Gene**	**AbSeq**	**10x**	**ratio**	**AbSeq**	**10x**	**ratio**	**Log Transform**
**LTC4S**	1	190	0.005263	0.000113	0.036315	0.003114	-2.506685491
**TGFB1**	91	3723	0.024443	0.010291	0.711583	0.014462	-1.839783534
**TNFRSF9**	2	72	0.027778	0.000226	0.013761	0.016435	-1.78423439
**S100A8**	452	13173	0.034313	0.051114	2.517775	0.020301	-1.692478147
**LTB**	1431	36656	0.039039	0.161823	7.006116	0.023097	-1.636437328
**XCL1**	91	1739	0.052329	0.010291	0.332378	0.030961	-1.509190079
**CD96**	234	3302	0.070866	0.026462	0.631116	0.041928	-1.377493101
**STAT3**	482	6598	0.073052	0.054506	1.261086	0.043222	-1.364297163
**FN1**	2	25	0.08	0.000226	0.004778	0.047332	-1.324841903
**GZMM**	716	7298	0.098109	0.080968	1.394878	0.058047	-1.236222726
**IFITM1**	886	7840	0.11301	0.100192	1.498471	0.066863	-1.17481423
**TNFRSF18**	84	653	0.128637	0.009499	0.124809	0.076109	-1.118565785
**BLK**	29	220	0.131818	0.003279	0.042049	0.077991	-1.107956573
**ZNF219**	13	95	0.136842	0.00147	0.018157	0.080963	-1.091712143
**ITGA1**	46	262	0.175573	0.005202	0.050076	0.103878	-0.983475349
**FOXP1**	1728	7714	0.224008	0.195409	1.474388	0.132535	-0.877667786
**NSG1**	376	1656	0.227053	0.04252	0.316514	0.134337	-0.871804377
**ADAM15**	95	416	0.228365	0.010743	0.079511	0.135113	-0.869301615
**FTH1**	14533	57656	0.252064	1.643447	11.01988	0.149135	-0.826421125
**ICOSLG**	57	200	0.285	0.006446	0.038226	0.168622	-0.77308703
**EOMES**	144	497	0.289738	0.016284	0.094992	0.171425	-0.765925786
**GATA3**	539	1842	0.292617	0.060952	0.352064	0.173128	-0.76163275
**KLF6**	8885	30044	0.295733	1.00475	5.742355	0.174972	-0.757032211
**KLRD1**	2669	9005	0.296391	0.301821	1.721139	0.175361	-0.756067033
**CD2**	4047	11140	0.363285	0.45765	2.129205	0.214939	-0.667683876
**CCR10**	37	98	0.377551	0.004184	0.018731	0.22338	-0.650956241
**CCR6**	91	215	0.423256	0.010291	0.041093	0.250421	-0.601328957
**ELANE**	3	7	0.428571	0.000339	0.001338	0.253566	-0.595908675
**PDCD1**	39	87	0.448276	0.00441	0.016628	0.265224	-0.576386535
**AZU1**	9	20	0.45	0.001018	0.003823	0.266244	-0.574719376
**TNFRSF25**	701	1460	0.480137	0.079272	0.279052	0.284075	-0.546566728
**KLRG1**	1872	3746	0.499733	0.211693	0.715979	0.295669	-0.529193818
**SPP1**	2	4	0.5	0.000226	0.000765	0.295827	-0.528961885
**CD3G**	6045	12089	0.500041	0.683592	2.310589	0.295852	-0.528925962
**NPDC1**	432	837	0.516129	0.048852	0.159977	0.30537	-0.515173601
**ENTPD1**	501	945	0.530159	0.056655	0.180619	0.313671	-0.503525972
**TCF7**	3723	7006	0.531402	0.421011	1.339067	0.314406	-0.502508986
**LAG3**	964	1799	0.535853	0.109013	0.343846	0.31704	-0.498886019
**TNFRSF4**	217	383	0.56658	0.024539	0.073203	0.335219	-0.47467093
**THBS1**	106	186	0.569892	0.011987	0.03555	0.337179	-0.472138969
**NCAM1**	60	105	0.571429	0.006785	0.020069	0.338088	-0.470969938
**JUN**	28082	45537	0.616685	3.175619	8.703555	0.364865	-0.43786827
**RORA**	4447	7132	0.623528	0.502884	1.36315	0.368913	-0.433076095
**FCGR3B**	9	14	0.642857	0.001018	0.002676	0.380349	-0.419817416
**FOS**	30993	47576	0.651442	3.504806	9.093272	0.385428	-0.414056199
**STAT4**	2825	4325	0.653179	0.319462	0.826644	0.386456	-0.412899549
**KLRC4**	217	316	0.686709	0.024539	0.060398	0.406294	-0.391159238
**ICOSLG**	141	200	0.705	0.015945	0.038226	0.417116	-0.379742773
**TIGIT**	659	917	0.718648	0.074522	0.175268	0.425191	-0.371415811
**CCL5**	64027	87628	0.730668	7.240416	16.74847	0.432303	-0.364211636
**CD7**	9127	12162	0.750452	1.032116	2.324541	0.444008	-0.352608838
**CD70**	261	345	0.756522	0.029515	0.06594	0.447599	-0.349110477
**IL1R2**	68	88	0.772727	0.00769	0.01682	0.457188	-0.339905649
**CNTNAP3**	4	5	0.8	0.000452	0.000956	0.473324	-0.324841903
**BATF**	480	575	0.834783	0.05428	0.109901	0.493903	-0.306358497
**KLRC1**	3488	4120	0.846602	0.394436	0.787462	0.500896	-0.300252629
**LAIR2**	348	403	0.863524	0.039353	0.077026	0.510908	-0.291657692
**GZMK**	11582	13098	0.884257	1.309737	2.50344	0.523175	-0.281353315
**TNFSF14**	199	224	0.888393	0.022504	0.042813	0.525622	-0.279326832
**PRDM1**	1608	1779	0.903879	0.181839	0.340023	0.534784	-0.271821793
**CD8B**	8578	9355	0.916943	0.970033	1.788035	0.542513	-0.26558964
**MKI67**	35	38	0.921053	0.003958	0.007263	0.544945	-0.263647442
**KLRC2**	1064	1078	0.987013	0.120321	0.20604	0.583971	-0.233609023
**GZMH**	16595	16177	1.025839	1.876626	3.091934	0.606942	-0.216852618
**GNLY**	102389	99026	1.033961	11.57854	18.92699	0.611747	-0.213427825
**BIRC5**	72	69	1.043478	0.008142	0.013188	0.617379	-0.209448484
**KIAA0101**	206	196	1.05102	0.023295	0.037462	0.621841	-0.206320741
**PTPRC**	27417	25635	1.069514	3.100418	4.899656	0.632783	-0.198745279
**CD44**	23465	21345	1.099321	2.653511	4.079702	0.650418	-0.18680749
**KLF3**	3971	3574	1.11108	0.449056	0.683104	0.657375	-0.182186551
**CD247**	6479	5403	1.199149	0.73267	1.032683	0.709482	-0.149058877
**PXDN**	10	8	1.25	0.001131	0.001529	0.739568	-0.131021877
**STAT1**	2917	2294	1.271578	0.329865	0.438456	0.752335	-0.123588874
**ATF6B**	5152	4016	1.282869	0.582608	0.767584	0.759015	-0.119749739
**TSPAN32**	3439	2651	1.297246	0.388895	0.50669	0.767522	-0.114909442
**THBD**	281	216	1.300926	0.031777	0.041284	0.769699	-0.113679321
**TNFRSF13C**	269	201	1.338308	0.03042	0.038417	0.791816	-0.101375667
**GZMA**	27871	20568	1.355066	3.151758	3.931193	0.801731	-0.095971402
**FCGR3B**	19	14	1.357143	0.002149	0.002676	0.80296	-0.095306324
**CD3D**	44895	32787	1.369293	5.076897	6.266628	0.810148	-0.091435594
**ZNF683**	2669	1922	1.388658	0.301821	0.367355	0.821605	-0.085336699
**TTC16**	829	585	1.417094	0.093746	0.111812	0.83843	-0.076533225
**ADAM17**	1951	1326	1.471342	0.220626	0.25344	0.870526	-0.060218144
**CXCR3**	1848	1232	1.5	0.208979	0.235474	0.887482	-0.051840631
**TCF4**	333	222	1.5	0.037657	0.042431	0.887482	-0.051840631
**BACH2**	1137	740	1.536486	0.128576	0.141437	0.909069	-0.041403145
**CD40LG**	457	294	1.554422	0.051679	0.056193	0.919681	-0.03636302
**IL12RB1**	1508	961	1.569199	0.17053	0.183677	0.928423	-0.032253936
**FAM65B**	14072	8786	1.601639	1.591315	1.679281	0.947617	-0.023367262
**CTSD**	6783	4220	1.607346	0.767047	0.806575	0.950993	-0.021822524
**JUNB**	97948	60365	1.622596	11.07633	11.53765	0.960016	-0.017721522
**ZAP70**	5975	3630	1.646006	0.675676	0.693807	0.973866	-0.011500605
**CD2**	18574	11140	1.667325	2.100418	2.129205	0.98648	-0.005911639
**STAT6**	2134	1248	1.709936	0.241321	0.238532	1.011691	0.00504794
**PSEN1**	1281	748	1.712567	0.14486	0.142966	1.013248	0.005715642
**PRDM1**	3114	1779	1.750422	0.352143	0.340023	1.035645	0.01521077
**CTSW**	30029	16711	1.79696	3.395793	3.193998	1.063179	0.026606541
**HLA-DQA1**	3100	1692	1.832151	0.35056	0.323394	1.084	0.035029445
**SBK1**	926	504	1.837302	0.104716	0.09633	1.087048	0.036248561
**NCR3**	3992	2171	1.838784	0.451431	0.414946	1.087925	0.036598819
**KCNK5**	24	13	1.846154	0.002714	0.002485	1.092285	0.038336
**CRTAM**	943	503	1.874751	0.106638	0.096139	1.109205	0.045011818
**CD9**	325	173	1.878613	0.036752	0.033066	1.11149	0.045905368
**IL7R**	32561	17238	1.888908	3.682121	3.294725	1.117581	0.048278968
**CD8A**	17171	9056	1.896091	1.941762	1.730887	1.121831	0.049927285
**CBLB**	4766	2472	1.927994	0.538957	0.472477	1.140706	0.057173682
**DOCK8**	6857	3522	1.946905	0.775416	0.673165	1.151895	0.061412908
**BCL11B**	8157	4188	1.947708	0.922425	0.800459	1.15237	0.0615919
**AIM2**	143	71	2.014085	0.016171	0.01357	1.191642	0.076145799
**NKG7**	108579	53730	2.020826	12.27853	10.2695	1.195631	0.077597107

	Raw Counts			Normalized to cell#			
**LYZ**	107433	21087	5.09475	12.14893	4.03039	3.014332	0.479191
**MGST1**	1018	199	5.115578	0.115119	0.038035	3.026654	0.480963
**CCL4**	22872	4458	5.130552	2.586453	0.852064	3.035514	0.482232
**CXCL5**	36	7	5.142857	0.004071	0.001338	3.042794	0.483273
**STK38**	7312	1420	5.149296	0.826869	0.271407	3.046604	0.483816
**AHR**	3205	621	5.161031	0.362434	0.118693	3.053547	0.484805
**CD274**	262	50	5.24	0.029628	0.009557	3.100269	0.491399
**CD80**	21	4	5.25	0.002375	0.000765	3.106186	0.492227
**BIN2**	33630	6386	5.266207	3.803008	1.220566	3.115775	0.493566
**FCGR3A**	25844	4868	5.308956	2.922538	0.930428	3.141068	0.497077
**RUNX3**	31623	5918	5.343528	3.576049	1.131116	3.161522	0.499896
**CD40**	1010	189	5.343915	0.114215	0.036124	3.161751	0.499928
**CD69**	82006	15340	5.345893	9.27355	2.931957	3.162921	0.500088
**FBLN2**	211	39	5.410256	0.023861	0.007454	3.201002	0.505286
**CCR2**	542	100	5.42	0.061291	0.019113	3.206767	0.506067
**RPN2**	14494	2653	5.463249	1.639037	0.507072	3.232355	0.509519
**CHI3L2**	1697	310	5.474194	0.191903	0.059251	3.238831	0.510388
**NT5E**	952	173	5.50289	0.107656	0.033066	3.255809	0.512659
**IL18R1**	1099	199	5.522613	0.124279	0.038035	3.267478	0.514213
**VCAN**	11662	2105	5.540143	1.318783	0.402332	3.27785	0.515589
**PTGDS**	3870	693	5.584416	0.437634	0.132454	3.304044	0.519046
**LAMP1**	20186	3604	5.600999	2.282709	0.688838	3.313856	0.520334
**FYN**	27226	4820	5.648548	3.078819	0.921254	3.341988	0.524005
**IL12A**	231	40	5.775	0.026122	0.007645	3.416804	0.53362
**ICOS**	2463	426	5.78169	0.278525	0.081422	3.420763	0.534123
**TNFSF13B**	3073	524	5.864504	0.347507	0.100153	3.46976	0.540299
**ADA**	5259	895	5.875978	0.594708	0.171063	3.476548	0.541148
**CD244**	2678	455	5.885714	0.302838	0.086965	3.482309	0.541867
**GIMAP2**	11943	2025	5.897778	1.35056	0.387041	3.489446	0.542757
**CD24**	427	72	5.930556	0.048287	0.013761	3.508839	0.545163
**CMTM2**	107	18	5.944444	0.0121	0.00344	3.517057	0.546179
**CD28**	4235	708	5.981638	0.47891	0.135321	3.539063	0.548888
**IL3RA**	565	94	6.010638	0.063892	0.017966	3.556221	0.550989
**CD48**	86413	14277	6.052602	9.77191	2.728784	3.581049	0.55401
**IFNGR1**	6866	1122	6.11943	0.776433	0.21445	3.620588	0.558779
**C1QB**	374	61	6.131148	0.042293	0.011659	3.627521	0.55961
**FCER1A**	3685	599	6.15192	0.416714	0.114488	3.639811	0.561079
**XCL2**	6559	1058	6.199433	0.741717	0.202217	3.667922	0.56442
**KLRF1**	9438	1515	6.229703	1.067285	0.289564	3.685831	0.566535
**IRF7**	4474	710	6.301408	0.505937	0.135703	3.728256	0.571506
**FUT4**	354	56	6.321429	0.040032	0.010703	3.740101	0.572883
**CCL22**	19	3	6.333333	0.002149	0.000573	3.747145	0.5737
**CCR3**	19	3	6.333333	0.002149	0.000573	3.747145	0.5737
**ITGAM**	6309	988	6.385628	0.713446	0.188838	3.778085	0.577272
**HLA-DPA1**	49085	7672	6.397941	5.550718	1.466361	3.78537	0.578108
**TLR2**	1785	278	6.420863	0.201855	0.053135	3.798932	0.579662
**S100A9**	99413	15416	6.44869	11.242	2.946483	3.815396	0.58154
**ZBTB16**	1880	291	6.460481	0.212598	0.055619	3.822372	0.582333
**MXRA8**	182	28	6.5	0.020581	0.005352	3.845754	0.584981
**SELPLG**	21267	3250	6.543692	2.404953	0.621177	3.871604	0.587891
**ANXA5**	13505	2023	6.675729	1.527197	0.386659	3.949725	0.596567
**IL2**	87	13	6.692308	0.009838	0.002485	3.959533	0.597644
**PCNA**	3715	552	6.730072	0.420106	0.105505	3.981877	0.600088
**VPREB3**	1515	225	6.733333	0.171322	0.043005	3.983806	0.600298
**CXCR4**	212805	31249	6.809978	24.0648	5.972668	4.029154	0.605214
**DPP4**	3983	584	6.820205	0.450413	0.111621	4.035205	0.605866
**LTA**	1769	255	6.937255	0.200045	0.048739	4.104457	0.613256
**AURKB**	174	25	6.96	0.019677	0.004778	4.117915	0.614677
**FOSB**	136646	19532	6.996007	15.45245	3.73318	4.139218	0.616918
**CLEC10A**	1699	242	7.020661	0.192129	0.046254	4.153805	0.618446
**LILRB4**	1500	213	7.042254	0.169626	0.040711	4.16658	0.61978
**TYMS**	361	51	7.078431	0.040823	0.009748	4.187985	0.622005
**TNF**	8171	1138	7.180141	0.924008	0.217508	4.248162	0.628201
**TNFRSF8**	216	30	7.2	0.024426	0.005734	4.259912	0.629401
**SELL**	46325	6315	7.335709	5.238607	1.206995	4.340204	0.63751
**LIPA**	8299	1130	7.344248	0.938482	0.215979	4.345257	0.638015
**ITGAX**	5106	695	7.346763	0.577406	0.132836	4.346745	0.638164
**CX3CR1**	3042	413	7.365617	0.344001	0.078937	4.3579	0.639277
**KCNE3**	914	124	7.370968	0.103359	0.0237	4.361066	0.639593
**TOX**	1894	255	7.427451	0.214181	0.048739	4.394484	0.642908
**CCR6**	1601	215	7.446512	0.181047	0.041093	4.405761	0.644021
**FCER1G**	31374	4177	7.511132	3.547891	0.798356	4.443995	0.647774
**VEGFA**	1533	204	7.514706	0.173357	0.038991	4.446109	0.64798
**CSF1**	279	37	7.540541	0.03155	0.007072	4.461394	0.649471
**TGFB3**	276	36	7.666667	0.031211	0.006881	4.536017	0.656675
**MZB1**	1741	227	7.669604	0.196879	0.043387	4.537755	0.656841
**ITGB2**	75666	9726	7.779766	8.556598	1.858945	4.602933	0.663035
**TRAT1**	7378	946	7.799154	0.834332	0.18081	4.614404	0.664116
**IER3**	13020	1638	7.948718	1.472351	0.313073	4.702894	0.672365
**B3GAT1**	916	115	7.965217	0.103585	0.02198	4.712656	0.673266
**HLA-DMA**	16420	2056	7.986381	1.856836	0.392966	4.725178	0.674418
**MYC**	6857	856	8.010514	0.775416	0.163609	4.739456	0.675728
**IL10**	163	20	8.15	0.018433	0.003823	4.821983	0.683226
**CLEC4D**	432	53	8.150943	0.048852	0.01013	4.822542	0.683276
**BCL2A1**	11911	1447	8.231513	1.346941	0.276567	4.870211	0.687548
**TBX21**	6745	819	8.235653	0.76275	0.156537	4.872661	0.687766
**TNFRSF1B**	18711	2262	8.271883	2.115911	0.432339	4.894096	0.689673
**CCL4L2**	4038	481	8.39501	0.456632	0.091934	4.966945	0.696089
**EGR3**	361	43	8.395349	0.040823	0.008219	4.967145	0.696107
**RNASE6**	3444	406	8.482759	0.389461	0.077599	5.018862	0.700605
**PIK3IP1**	48078	5661	8.492846	5.436843	1.081995	5.02483	0.701121
**TMEM123**	56255	6621	8.496451	6.361529	1.265482	5.026963	0.701306
**RNASE2**	1271	149	8.530201	0.14373	0.028479	5.046931	0.703027
**CCR7**	10834	1265	8.564427	1.22515	0.241781	5.067181	0.704766
**IRF4**	1234	140	8.814286	0.139545	0.026758	5.215011	0.717255
**LAMP3**	287	32	8.96875	0.032455	0.006116	5.306401	0.7248
**JCHAIN**	6644	739	8.990528	0.751329	0.141246	5.319285	0.725853
**CD207**	54	6	9	0.006107	0.001147	5.32489	0.726311
**HLA-DRA**	113741	12396	9.175621	12.86226	2.369266	5.428797	0.734704
**CXCR1**	83	9	9.222222	0.009386	0.00172	5.456369	0.736904
**LAT2**	5318	568	9.362676	0.60138	0.108563	5.539469	0.743468
**IFITM3**	26465	2796	9.465308	2.992763	0.534404	5.600191	0.748203
**ADGRE1**	853	90	9.477778	0.09646	0.017202	5.607569	0.748775
**FLT3**	418	44	9.5	0.047269	0.00841	5.620717	0.749792
**ITGA5**	5034	527	9.552182	0.569264	0.100726	5.651591	0.752171
**BTLA**	643	67	9.597015	0.072713	0.012806	5.678116	0.754204
**EGR1**	7898	822	9.608273	0.893136	0.15711	5.684777	0.754713
**STAT5A**	6542	668	9.793413	0.739794	0.127676	5.794316	0.763002
**PTPN6**	24415	2483	9.832863	2.760941	0.47458	5.817657	0.764748
**FCER2**	1191	120	9.925	0.134683	0.022936	5.87217	0.768799
**LIF**	50	5	10	0.005654	0.000956	5.916544	0.772068

**VPS28**	9035	4417	2.045506	1.021712	0.844228	1.210233	0.082868868
**VMO1**	198	96	2.0625	0.022391	0.018349	1.220287	0.086462068
**IFITM2**	33211	16071	2.066517	3.755626	3.071674	1.222664	0.087307162
**KDELR1**	2400	1156	2.076125	0.271401	0.220948	1.228348	0.089321518
**TXK**	5293	2484	2.130837	0.598553	0.474771	1.260719	0.100618413
**PDCD1LG2**	30	14	2.142857	0.003393	0.002676	1.267831	0.103061329
**FOXO3**	1971	915	2.154098	0.222888	0.174885	1.274482	0.105333641
**CLU**	446	204	2.186275	0.050435	0.038991	1.293519	0.111772802
**ALAS2**	20	9	2.222222	0.002262	0.00172	1.314788	0.118855597
**ZBED2**	9	4	2.25	0.001018	0.000765	1.331222	0.124250628
**IKZF1**	9403	4146	2.267969	1.063327	0.792431	1.341854	0.127705249
**RAP1GAP2**	1608	708	2.271186	0.181839	0.135321	1.343757	0.128320897
**CD27**	14646	6431	2.277406	1.656225	1.229167	1.347438	0.129508631
**TMEM97**	258	112	2.303571	0.029176	0.021407	1.362918	0.134469794
**TIMP1**	12975	5616	2.310363	1.467262	1.073394	1.366937	0.135748378
**AIF1**	12935	5575	2.320179	1.462739	1.065558	1.372744	0.137589672
**IL15**	419	179	2.340782	0.047382	0.034213	1.384934	0.141429102
**HAVCR2**	1493	634	2.35489	0.168834	0.121177	1.393281	0.14403866
**GNAI2**	8341	3509	2.37703	0.943232	0.67068	1.406381	0.148102863
**DUSP4**	859	357	2.406162	0.097139	0.068234	1.423617	0.153393058
**TLR7**	85	35	2.428571	0.009612	0.00669	1.436875	0.157418992
**BIRC3**	4154	1674	2.481481	0.46975	0.319954	1.468179	0.166779149
**CMKLR1**	354	142	2.492958	0.040032	0.027141	1.474969	0.168783028
**HOPX**	15668	6259	2.503275	1.771797	1.196292	1.481074	0.170576722
**SLC25A37**	3300	1307	2.524866	0.373177	0.249809	1.493848	0.174306463
**TREM1**	1645	646	2.54644	0.186023	0.123471	1.506612	0.178001495
**EBF1**	161	63	2.555556	0.018206	0.012041	1.512006	0.179553437
**SOCS3**	5137	2006	2.560818	0.580911	0.38341	1.515119	0.180446747
**APOBEC3G**	8256	3215	2.567963	0.93362	0.614488	1.519346	0.181656817
**IL32**	127779	48976	2.609013	14.44973	9.360856	1.543634	0.188544283
**CCR1**	444	170	2.611765	0.050209	0.032492	1.545262	0.189002159
**CD160**	2000	764	2.617801	0.226168	0.146024	1.548834	0.190004747
**LGALS1**	30948	11401	2.714499	3.499717	2.17909	1.606045	0.205757753
**CD52**	185215	67835	2.730375	20.94482	12.96541	1.615439	0.208290437
**PRF1**	17485	6395	2.734167	1.97727	1.222286	1.617682	0.208893198
**MMP9**	22	8	2.75	0.002488	0.001529	1.62705	0.211400804
**PDIA4**	4171	1506	2.769588	0.471673	0.287844	1.638639	0.214483328
**GZMB**	18568	6691	2.775071	2.09974	1.278861	1.641883	0.215342208
**FAM129C**	345	124	2.782258	0.039014	0.0237	1.646135	0.21646552
**IL23A**	1429	508	2.812992	0.161597	0.097095	1.664319	0.221236627
**CST7**	29835	10522	2.835488	3.373855	2.011086	1.677629	0.224695855
**IL6R**	1817	629	2.888712	0.205473	0.120222	1.709119	0.232772392
**TOP2A**	203	70	2.9	0.022956	0.013379	1.715798	0.234466108
**BCL2**	5023	1696	2.961675	0.56802	0.324159	1.752288	0.243605441
**MCM4**	409	138	2.963768	0.046251	0.026376	1.753526	0.243912332
**LAT**	19297	6405	3.012802	2.182178	1.224197	1.782538	0.251038773
**BTG1**	224167	72851	3.077061	25.34966	13.92412	1.820557	0.260204272
**CXCR6**	133	43	3.093023	0.01504	0.008219	1.830001	0.262451296
**NRP1**	19	6	3.166667	0.002149	0.001147	1.873572	0.272670461
**CD38**	606	187	3.240642	0.068529	0.035742	1.91734	0.282699128
**DUSP2**	63937	19729	3.240762	7.230239	3.770833	1.917411	0.282715292
**IKZF2**	869	266	3.266917	0.09827	0.050841	1.932886	0.28620625
**FYB**	33389	10174	3.281797	3.775755	1.944572	1.94169	0.288179789
**PDIA6**	9147	2772	3.299784	1.034377	0.529817	1.952332	0.290553563
**CTSS**	30120	9084	3.31572	3.406084	1.736239	1.96176	0.292645952
**ADAM28**	959	286	3.353147	0.108447	0.054664	1.983904	0.297520684
**NINJ2**	1106	325	3.403077	0.125071	0.062118	2.013445	0.303939876
**AXL**	82	24	3.416667	0.009273	0.004587	2.021486	0.305670721
**MX2**	3497	1021	3.425073	0.395454	0.195145	2.02646	0.306738001
**YBX3**	7574	2211	3.425599	0.856497	0.422592	2.026771	0.306804669
**GAPDH**	84429	24641	3.426363	9.547552	4.709671	2.027223	0.306901427
**NAMPT**	14697	4277	3.436287	1.661993	0.817469	2.033094	0.308157554
**HLA-DPB1**	36490	10609	3.439532	4.126428	2.027714	2.035015	0.308567524
**IFNG**	3881	1120	3.465179	0.438878	0.214067	2.050188	0.31179373
**XBP1**	18546	5304	3.496606	2.097252	1.013761	2.068783	0.31571485
**CD36**	1941	553	3.509946	0.219496	0.105696	2.076675	0.317368514
**CD63**	23331	6618	3.525385	2.638358	1.264908	2.08581	0.319274701
**CD79B**	5205	1466	3.550477	0.588601	0.280199	2.100656	0.322354874
**ID3**	1059	291	3.639175	0.119756	0.055619	2.153134	0.333071081
**MITF**	73	20	3.65	0.008255	0.003823	2.159539	0.334360975
**CCND2**	5268	1425	3.696842	0.595725	0.272362	2.187253	0.339899012
**C1QA**	584	155	3.767742	0.066041	0.029625	2.229201	0.348149259
**KLRB1**	39182	10383	3.773668	4.430849	1.984518	2.232708	0.348831857
**CD34**	38	10	3.8	0.004297	0.001911	2.248287	0.351851707
**SOX13**	478	125	3.824	0.054054	0.023891	2.262486	0.354585994
**MNDA**	4667	1204	3.876246	0.527762	0.230122	2.293398	0.360479424
**IGBP1**	8275	2122	3.899623	0.935768	0.405581	2.307229	0.363090733
**CNOT2**	6596	1667	3.956809	0.745901	0.318616	2.341063	0.369413157
**HLA-A**	452441	113704	3.979112	51.16375	21.73242	2.354259	0.371854321
**BAX**	12273	3068	4.000326	1.387877	0.586391	2.366811	0.374163489
**KLF2**	37252	9229	4.036407	4.212598	1.763953	2.388158	0.378063059
**MX1**	3174	784	4.048469	0.358928	0.149847	2.395295	0.37935897
**LCK**	35362	8707	4.06133	3.998869	1.664182	2.402904	0.380736386
**ARL4C**	48347	11865	4.074758	5.467262	2.267775	2.410848	0.382169898
**DUSP6**	4376	1064	4.112782	0.494855	0.203364	2.433346	0.386203796
**LAP3**	4002	967	4.138573	0.452561	0.184824	2.448605	0.388918721
**ITGAE**	4388	1060	4.139623	0.496212	0.202599	2.449226	0.389028864
**IL2RA**	820	197	4.162437	0.092729	0.037653	2.462724	0.391415737
**HIF1A**	10460	2506	4.173982	1.182856	0.478976	2.469555	0.392618728
**UBE2C**	88	21	4.190476	0.009951	0.004014	2.479314	0.394331488
**LGALS3**	8537	2008	4.251494	0.965396	0.383792	2.515415	0.400609683
**S100A10**	64095	14993	4.274995	7.248106	2.865635	2.52932	0.403003721
**CCR5**	352	79	4.455696	0.039805	0.015099	2.636232	0.420983682
**CD300A**	6625	1462	4.531464	0.74918	0.279434	2.681061	0.42830662
**ITGA4**	12828	2805	4.573262	1.450639	0.536124	2.705791	0.432294196
**CD3E**	62665	13622	4.600279	7.086396	2.603593	2.721775	0.434852278
**FAS**	2328	506	4.600791	0.263259	0.096713	2.722078	0.434900569
**IL15RA**	817	176	4.642045	0.092389	0.033639	2.746487	0.438777499
**FASLG**	288	62	4.645161	0.032568	0.01185	2.74833	0.439068909
**HLA-DQB1**	21662	4637	4.671555	2.449621	0.886277	2.763946	0.441529566
**CDH1**	38	8	4.75	0.004297	0.001529	2.810358	0.44876172
**TRAC**	103183	21659	4.763978	11.66833	4.139717	2.818629	0.45003786
**CCR4**	106	22	4.818182	0.011987	0.004205	2.850699	0.454951295
**SNCA**	323	67	4.820896	0.036526	0.012806	2.852304	0.45519583
**C10orf54**	25072	5148	4.870241	2.835237	0.983945	2.8815	0.459618551
**CD37**	59955	12251	4.893886	6.779939	2.341552	2.895489	0.461721978
**FBN2**	55	11	5	0.00622	0.002102	2.958272	0.471038115
**MME**	40	8	5	0.004523	0.001529	2.958272	0.471038115
**HMMR**	35	7	5	0.003958	0.001338	2.958272	0.471038115
**BCL6**	2847	569	5.003515	0.32195	0.108754	2.960352	0.471343311
**TNFSF10**	3903	779	5.01027	0.441366	0.148891	2.964348	0.471929204
**FOXO1**	2900	575	5.043478	0.327943	0.109901	2.983996	0.474798264
**CD72**	772	153	5.045752	0.087301	0.029243	2.985341	0.47499398

**CD86**	2138	213	10.03756	0.241773	0.040711	5.938766	0.773696
**RHOA**	71167	7090	10.03766	8.047834	1.355122	5.938825	0.773701
**F5**	784	78	10.05128	0.088658	0.014908	5.946885	0.77429
**CD5**	11114	1090	10.19633	1.256813	0.208333	6.032704	0.780512
**CLC**	31	3	10.33333	0.003506	0.000573	6.113762	0.786309
**MS4A1**	8859	854	10.37354	1.001809	0.163226	6.137549	0.787995
**LY86**	5891	562	10.48221	0.666177	0.107416	6.201844	0.792521
**CD209**	42	4	10.5	0.00475	0.000765	6.212371	0.793257
**TIMP2**	2725	258	10.56202	0.308153	0.049312	6.249063	0.795815
**CXCL10**	74	7	10.57143	0.008368	0.001338	6.254632	0.796202
**IL4R**	12609	1189	10.60471	1.425874	0.227255	6.274323	0.797567
**CD74**	261924	24617	10.63996	29.61936	4.705084	6.295182	0.799008
**TNFSF8**	4195	392	10.70153	0.474387	0.074924	6.331608	0.801514
**IL18**	516	48	10.75	0.058351	0.009174	6.360285	0.803477
**LYN**	10189	932	10.9324	1.152211	0.178135	6.468205	0.810784
**CD6**	32129	2929	10.96927	3.633269	0.559824	6.490019	0.812246
**QPCT**	1067	96	11.11458	0.12066	0.018349	6.575992	0.817961
**VNN2**	3607	323	11.16718	0.407893	0.061735	6.607113	0.820012
**TSPAN18**	2308	203	11.36946	0.260997	0.0388	6.72679	0.827808
**VSIG4**	205	18	11.38889	0.023182	0.00344	6.738286	0.828549
**TRBC2**	149823	13083	11.45173	16.94255	2.500573	6.775467	0.830939
**CD33**	2294	200	11.47	0.259414	0.038226	6.786276	0.831632
**IGKC**	55156	4770	11.5631	6.23725	0.911697	6.841361	0.835142
**CD79A**	17291	1494	11.57363	1.955332	0.28555	6.847588	0.835538
**ICAM1**	2634	226	11.65487	0.297863	0.043196	6.895654	0.838575
**IL12RB2**	317	27	11.74074	0.035848	0.005161	6.946461	0.841764
**F13A1**	1549	131	11.82443	0.175167	0.025038	6.995975	0.844848
**CCL3L3**	15572	1283	12.13718	1.760941	0.245222	7.181015	0.856186
**SLC7A7**	3434	280	12.26429	0.38833	0.053517	7.256219	0.86071
**FOS**	585716	47576	12.31117	66.23499	9.093272	7.283955	0.862367
**LGALS9**	7372	593	12.4317	0.833654	0.113341	7.355272	0.866599
**CXCR2**	411	33	12.45455	0.046477	0.006307	7.368787	0.867396
**GAB2**	1244	99	12.56566	0.140676	0.018922	7.434526	0.871253
**TRDC**	11861	926	12.80886	1.341287	0.176988	7.578416	0.879578
**CD200**	334	26	12.84615	0.03777	0.004969	7.600484	0.880841
**CD1A**	65	5	13	0.00735	0.000956	7.691507	0.886011
**TLR8**	496	38	13.05263	0.05609	0.007263	7.722647	0.887766
**PIK3AP1**	5677	434	13.08065	0.641977	0.082951	7.739221	0.888697
**CD14**	16036	1222	13.12275	1.813412	0.233563	7.764133	0.890093
**PTTG2**	132	10	13.2	0.014927	0.001911	7.809838	0.892642
**TTYH2**	2231	168	13.27976	0.25229	0.03211	7.85703	0.895258
**FCN1**	50958	3809	13.37831	5.762524	0.72802	7.915339	0.89847
**CAPG**	9453	705	13.40851	1.068981	0.134748	7.933205	0.899449
**CLEC4E**	2507	186	13.47849	0.283501	0.03555	7.974611	0.90171
**CR2**	445	33	13.48485	0.050322	0.006307	7.97837	0.901914
**LEF1**	28736	2128	13.50376	3.249576	0.406728	7.989559	0.902523
**AQP9**	907	67	13.53731	0.102567	0.012806	8.009411	0.903601
**CCR9**	122	9	13.55556	0.013796	0.00172	8.020204	0.904185
**IL2RB**	27617	1986	13.90584	3.123035	0.379587	8.227452	0.915265
**BLNK**	1032	74	13.94595	0.116702	0.014144	8.251181	0.916516
**PTGDR2**	224	16	14	0.025331	0.003058	8.283162	0.918196
**PASK**	10390	737	14.09769	1.174941	0.140864	8.340963	0.921216
**SERPINA1**	22155	1552	14.27513	2.505371	0.296636	8.445943	0.926648
**DUSP1**	361619	24942	14.4984	40.89325	4.767202	8.57804	0.933388
**CD1C**	2921	201	14.53234	0.330318	0.038417	8.598122	0.934404
**IRF8**	9205	632	14.56487	1.040936	0.120795	8.617372	0.935375
**S100A12**	18492	1258	14.69952	2.091146	0.240443	8.697038	0.939371
**CCL3**	28499	1835	15.53079	3.222775	0.350726	9.188861	0.963262
**CD163**	1679	103	16.30097	0.189868	0.019687	9.644541	0.984282
**CXCL16**	6149	374	16.44118	0.695352	0.071483	9.727495	0.988001
**IL18RAP**	3289	199	16.52764	0.371933	0.038035	9.77865	0.990279
**TRIB2**	7065	418	16.90191	0.798937	0.079893	10.00009	1.000004
**TCL1A**	1785	105	17	0.201855	0.020069	10.05813	1.002517
**MCM2**	1011	59	17.13559	0.114328	0.011277	10.13835	1.005967
**CTSK**	1377	79	17.43038	0.155716	0.015099	10.31276	1.013375
**RORC**	598	33	18.12121	0.067624	0.006307	10.7215	1.030255
**TGFBI**	9661	511	18.90607	1.092503	0.097668	11.18586	1.048669
**LRRC32**	95	5	19	0.010743	0.000956	11.24143	1.050822
**RGS1**	52303	2656	19.69239	5.914622	0.507645	11.65109	1.066367
**S1PR1**	18459	933	19.78457	2.087414	0.178326	11.70563	1.068395
**KLF2**	183528	9229	19.88601	20.75404	1.763953	11.76565	1.070616
**ZFP36**	444245	22249	19.96696	50.23691	4.252485	11.81354	1.07238
**CD68**	16656	797	20.89837	1.883524	0.152332	12.36461	1.092181
**CD83**	22925	1090	21.03211	2.592446	0.208333	12.44374	1.094951
**CD22**	4808	219	21.95434	0.543707	0.041858	12.98938	1.113588
**PAX5**	1647	75	21.96	0.186249	0.014335	12.99273	1.1137
**NLRP3**	8643	371	23.2965	0.977383	0.07091	13.78347	1.139359
**FOSL1**	573	23	24.91304	0.064797	0.004396	14.73991	1.168495
**CD4**	18434	717	25.7099	2.084587	0.137041	15.21138	1.182169
**TNFRSF17**	182	7	26	0.020581	0.001338	15.38301	1.187041
**CXCL2**	24769	940	26.35	2.800973	0.179664	15.59009	1.192849
**IL1B**	64692	2356	27.4584	7.315617	0.450306	16.24589	1.210743
**CTLA4**	2023	70	28.9	0.228769	0.013379	17.09881	1.232966
**KLRC3**	1537	53	29	0.17381	0.01013	17.15798	1.234466
**CXCL8**	139272	4538	30.69017	15.74941	0.867355	18.15798	1.259067
**IL1RN**	3121	101	30.90099	0.352935	0.019304	18.28271	1.262041
**CXCR5**	1780	56	31.78571	0.201289	0.010703	18.80616	1.2743
**CXCL3**	10693	325	32.90154	1.209205	0.062118	19.46634	1.289284
**GIMAP5**	4978	148	33.63514	0.562931	0.028287	19.90038	1.298861
**KLRK1**	15327	444	34.52027	1.733235	0.084862	20.42407	1.310142
**BPI**	312	9	34.66667	0.035282	0.00172	20.51069	1.31198
**ADGRG3**	178	5	35.6	0.020129	0.000956	21.0629	1.323518
**IGLC3**	64901	1387	46.79236	7.339251	0.265099	27.68491	1.442243
**KIR2DL1**	764	14	54.57143	0.086396	0.002676	32.28743	1.509033
**POU2AF1**	2240	41	54.63415	0.253308	0.007836	32.32453	1.509532
**IL23R**	3123	48	65.0625	0.353161	0.009174	38.49452	1.585399
**FOXP3**	1363	19	71.73684	0.154133	0.003631	42.44342	1.62781
**CXCL1**	1868	22	84.90909	0.211241	0.004205	50.23684	1.701022
**IL6**	2503	21	119.1905	0.283049	0.004014	70.51957	1.84831
**CCL20**	2024	13	155.6923	0.228882	0.002485	92.11604	1.964335
**TNFSF13**	3565	7	509.2857	0.403144	0.001338	301.3211	2.47903
**TIAF1**	5517	8	689.625	0.623883	0.001529	408.0197	2.610681
**CASP5**	9095	4	2273.75	1.028497	0.000765	1345.274	3.128811

Supplementary Table 3, related to main figure 2
List of genes overlapping/only detect in one assay between targeted transcriptomics and WTA in the indicated cluste

CD4 cluster

Gene overlap	Targeted only	WTA only
JUNB	LEF1	LTB
IL7R	TMEM123	MALAT1
TRAC	FYB	ZFP36L2
CD69	FOS	RPS21
CD3D	PIK3IP1	SARAF
BTG1	CD4	AAK1
CXCR4	KLF2	PABPC1
CD44	CD6	GSTK1
	CD48	ACAP1
	CD27	FXYD5
	TRBC2	RP11-347P5.1
	SELL	TRBC1
	CD3E	ITGB1
	KLF2	SLC2A3
	TRAT1	SRSF7
	CD52	TSC22D3
	LAT	SF1
	FOS	CCNL1
	CD5	PRRC2C
	CCR7	N4BP2L2
	FOSB	
	IL32	
	S1PR1	
	FAM65B	
	BCL11B	
	ITGA4	
	GIMAP2	
	TRIB2	
	RGS1	
	CD2	
	LCK	
	XBP1	
	IKZF1	
	RPN2	
	IL4R	
	VPS28	
	STK38	
	CD3G	
	ARL4C	
	FYN	
	PTPRC	
	KLF6	
	TIMP1	

NK cluster

Gene overlap	Targeted only	WTA only	
NKG7	TRDC	FGFBP2	FAM49B
PRF1	IL2RB	SPON2	PDIA3
GZMB	TBX21	TYROBP	CALR
GNLY	KLRK1	CLIC3	IL2RG
FCGR3A	HOPX	S1PR5	RP11-160E2.6
KLRD1	KLRC1	HLA-C	EVL
CTSW	RUNX3	CMC1	LCP1
GZMA	ITGAM	PLAC8	H3F3A
KLRF1	CX3CR1	GZMM	HNRNPA2B1
CST7	ITGAX	ACTB	SRP14
CD247	CD160	HLA-A	PSME1
KLRB1	CD300A	EFHD2	TAPBP
GZMH	CD244	HLA-B	DDX6
CD7	CCL3	MYO1F	CD53
CCL4	XCL2	ZEB2	LY6E
CCL5	NCR3	PFN1	PPP2R5C
FCER1G	LAT2	UBB	AES
ITGB2	IL18RAP	HCST	MYL12B
IFITM2	TARP	CLIC1	HNRNPA3
CTSD	APOBEC3G	ACTG1	ID2
CD63	PIK3AP1	ARPC2	SERF2
BIN2	LYN	HLA-E	MYL6
ARL4C	IFNG	JAK1	CCDC12
	TXK	SPN	SH3BGRL3
	IFITM3	FLNA	COX6C
	ZAP70	MYL12A	SSR2
	SELPLG	CFL1	RSRP1
	LAMP1	SUN2	PPIA
	STK38	CALM1	ZFP36
		CCND3	HNRNPK
		CYBA	
		ANXA6	
		PSMB9	
		CD99	
		RAC2	
		UCP2	
		43715	
		LSP1	
		RARRES3	
		LITAF	
		RNF213	
		IRF1	
		CORO1A	

Monocyte cluster

Gene overlap	Targeted only	WTA only		
LYZ	CLEC4E	SAT1	PTPRE	ATP6V0B
FCN1	TREM1	NEAT1	MT-ND1	PTP4A2
FTH1	SLC7A7	FTL	PHACTR1	CMTM6
NAMPT	NLRP3	CLEC7A	HLA-DRB5	ARRB2
S100A9	TGFBI	S100A8	GPX1	POU2F2
VCAN	TLR2	CSF3R	S100A11	VIM
CTSS	CXCL16	SLC11A1	CEBPD	KLF6
FOS	CXCL2	TYMP	RP11-386I14.4	TALDO1
FOS	RNASE2	PLAUR	AP1S2	RP5-1171I10.5
SERPINA1	KCNE3	RP11-1143G9.	JUND	FMNL1
CXCL8	LGALS3	CST3	ZEB2	ANXA1
CD14	CCL3L3	PSAP	RHOB	FKBP1A
MNDA	CD163	KLF4	HLA-DRB1	SDCBP
IL1B	MGST1	LINC00936	GABARAPL1	AP2S1
AIF1	VNN2	SPI1	PTCH2	WSB1
DUSP1	CD33	SGK1	RNF130	JMJD1C
HIF1A	QPCT	CFD	TKT	ACTR2
S100A12	BCL6	MT-CO1	NPC2	LCP1
CD36	TIMP2	IFI30	CTSB	GLIPR1
CD68	VEGFA	RP11-160E2.6	FCGRT	ANXA2
DUSP6	GAB2	G0S2	LYST	LAMTOR4
FCER1G	LAP3	FGL2	MXD1	C4orf48
BCL2A1	CD86	LST1	GPCPD1	LRRFIP1
HLA-DRA	CCL3	MAFB	APLP2	TCOF1
IER3	F13A1	CYBB	SOD2	TLE4
HLA-DQB1	ITGAX	SRGN	FOSL2	NR4A2
TNFRSF1B	TNFSF10	CEBPB	STXBP2	MYO1F
FOSB	YBX3	MCL1	TMEM107	LAPTM4A
LYN	IFNGR1	RGS2	VMP1	DDX21
CD74	ANXA5	CD300E	H2AFY	STK17B
IFITM3	CD83	S100A6	S100A4	SAT2
TIMP1	SLC25A37	CLEC12A	PYCARD	RTN4
ZFP36	RNASE6	MPEG1	ZFAND5	TUBA1A
LGALS1	LAT2	CH17-373J23.	JAML	UBC
CTSD	CAPG	GLUL	CTNNB1	VASP
HLA-DMA	SOCS3	C5AR1	MEF2C	EVI2B
C10orf54	AHR	TYROBP	WDR74	IRF2BP2
HLA-DPA1	LIPA	TNFAIP2	FGR	IQGAP1
HLA-DPB1	LY86	NFKBIA	ZFP36L1	CD44
	LGALS9	LILRB2	MYADM	NOP10
	IRF8	CSTA	ATP2B1	RNH1
	TNFSF13B	RAB31	MIDN	CAPZA1
	PIK3AP1	MS4A6A	PLEK	DAZAP2
	CD63	GRN	RNU12	HNRNPU
	TNFSF13	COTL1	GNAI2	DDX3X
	LILRB4	LGALS2	RAC1	VAPA
	PTPN6	CFP	C1orf162	EFHD2
	FCGR3A	ASAH1	GSTP1	SAMHD1
		SLC43A2	HLA-DMB	IL10RA
		NFKBIZ	NCF1	ATF4

Supplementary Table 4, related to STAR methods
List of staining dilutions and fluorochrome-conjugates used

FACSymphony **Panel 1 - T cell centric**

		Flourophore	Antigen	Dilution
1	355nm	BUV395	CD183 (CXCR3)	1: 20
2		UV Blue L/D	N.A	1: 500
3		BUV496	CD3	1: 40
4		BUV563	CD25	1: 40
5		BUV661	HLA-DR	1: 80
6		BUV737	ICOS	1: 20
7		BUV805	CD8	1: 80
8	405nm	BV421	CD196 (CCR6)	1: 20
9		BV480	TCRgd	1: 20
10		BV570	CD14	1: 20
11		BV605	PD1	1: 20
12		BV650	CD69	1: 20
13		BV711	CD45RA	1: 320
14		BV750	CD103	1: 160
15		BV785	CD127	1: 10
16	488nm	BB515	Tim3	1: 80
17		BB630	CD16	1: 1280
18		BB660	CD27	1: 160
19		BB700	CD161	1: 20
20		BB790	CD38	1: 80
21	532nm	PE	CD194 (CCR4)	1: 20
22		PE-CF594	CD39	1: 80
23		PE-Cy5	CD137	1: 20
24		PE-Cy5.5	CD19	1: 160
25		PE-Cy7	CD197 (CCR7)	1: 40
26	628nm	APC	EBV-TET	1: 200
27		AF700	CD45RO	1: 20
28		APC-H7	CD4	1: 40

FACSymphony **Panel 2 - APC centric**

		Flourophore	Antigen	Dilution
1	355nm	BUV395	CD40	1: 40
2		UV Blue L/D	N.A	1: 500
3		BUV496	CD3	1: 40
4		BUV563	CD56	1: 160
5		BUV661	EMPTY	
6		BUV737	CD86	1: 20
7		BUV805	CD8	1: 40
8	405nm	BV421	CX3CR1	1: 20
9		BV480	CD28	1: 40
10		BV570	CD14	1: 20
11		BV605	CD141	1: 320
12		BV650	Sirpa	1: 160
13		BV711	OX40	1: 40
14		BV750	CD11b	1: 160
15		BV785	CD123	1: 40
16	488nm	BB515	CD206	1: 20
17		BB630	CD16	1: 1280
18		BB660	CD27	1: 160
19		BB700	CD32	1: 160
20		BB790	CD38	1: 80
21	532nm	PE	Lag3	1: 20
22		PE-CF594	CD163	1: 80
23		PE-Cy5	CD80	1: 10
24		PE-Cy5.5	CD19	1: 160
25		PE-Cy7	CD4	1: 80
26	628nm	AF647	CD1c	1: 160
27		AF700	CD11c	1: 320
28		APC-H7	HLA-DR	1: 40

Transcriptomics - Oligonucleotide-Antibodies

AbSeq-AbO		Stock mg/ml	µg used per stain
1	CD3	0.125	0.25
2	CD4	0.03	0.06
3	CD8	0.125	0.25
4	CD19	0.03	0.06
5	CD14	0.125	0.25
6	CD16	0.25	0.5
7	CD56	0.0625	0.125
8	CD11b	0.03	0.06
9	CD25	0.125	0.25
10	HLA-DR	0.5	1
11	CD45RA	0.0625	0.125
12	CD127	0.125	0.25
13	CD38	0.25	0.5
14	CD197	0.25	0.5
15	CD279	0.25	0.5
16	CD28	0.125	0.25
17	CD279	0.125	0.25
18	CD69	0.125	0.25
19	CD123	0.25	0.5
20	CD45RO	0.25	0.5
21	CD11c	0.25	0.5
22	CD86	0.0625	0.125
23	CD183	0.5	1
24	CD196	0.25	0.5
25	CD80	0.125	0.25
26	CD278	0.03	0.06
27	CD194	0.25	0.5
28	CD40	0.015	0.03
29	CD137	0.5	1
30	TCRgd	0.5	1
31	CD163	0.5	1
32	CD134	0.5	1
33	Tim3	0.25	0.5
34	CD103	0.0625	0.125
35	CD206	0.25	0.5
36	CD32	0.5	1
37	CD161	0.25	0.5
38	CD39	0.25	0.5
39	CD141	0.125	0.25
40	Lag3	0.5	1
41	CD1c	0.25	0.5
